# Verbal Fluency Selectively Predicts Survival in Old and Very Old Age

**DOI:** 10.1177/09567976241311923

**Published:** 2025-02-24

**Authors:** Paolo Ghisletta, Stephen Aichele, Denis Gerstorf, Angela Carollo, Ulman Lindenberger

**Affiliations:** 1 Faculty of Psychology and Educational Sciences, University of Geneva; 2 Department of Human Development and Family Studies, Colorado State University; 3 Faculty of Epidemiology, Colorado School of Public Health; 4 Department of Psychology, Humboldt University; 5 German Socio-Economic Panel Study, German Institute for Economic Research, Berlin, Germany; 6 Laboratory of Fertility and Well-Being, Max Planck Institute for Demographic Research, Rostock, Germany; 7 Center for Lifespan Psychology, Max Planck Institute for Human Development, Berlin, Germany; 8 Max Planck UCL Centre for Computational Psychiatry and Ageing Research, Berlin, Germany

**Keywords:** cognition, aging, verbal fluency, joint multivariate longitudinal survival model, mixed-effects model, Cox survival model

## Abstract

Intelligence is known to predict survival, but it remains unclear whether cognitive abilities differ in their relationship to survival in old age. We analyzed longitudinal data of 516 healthy adults (age: *M* = 84.92 years, *SD* = 8.66 years at Wave 1) from the Berlin Aging Study (Germany) on nine tasks of perceptual speed, episodic memory, verbal fluency, and verbal knowledge, and a general composite intelligence score. There were eight waves, with up to 18 years of follow-up; all participants were deceased by the time of analysis. We used a joint multivariate longitudinal survival model to estimate the unique contribution of each cognitive ability in terms of true (i.e., error-free) current value and current rate of change when predicting survival. Additional survival covariates included age at first occasion, sex, sociobiographical status, and suspected dementia. Only the two verbal-fluency measures were uniquely predictive of mortality risk. Thus, verbal fluency showed more salient associations with mortality risk than did measures of perceptual speed, episodic memory, and verbal knowledge.

## Introduction

In humans, intelligence predicts survival. This has been corroborated by many independent studies and literature reviews, which were based on varying assessments of cognitive performance across different segments of the life span, and which used different analytical procedures (e.g., Aichele et al., 2016; Anstey et al., 2006; Bäckman & MacDonald, 2006; Batterham et al., 2012; Deary et al., 2004; Ghisletta, 2008). This literature is predominantly based on a single-occasion assessment of cognitive performance, which allows testing associations between level of cognition and mortality but precludes any inferences about the effects of cognitive change on survival. Some studies have leveraged longitudinal measurements of intellectual functioning, but their findings are inconsistent, mainly because of two methodological aspects. First, they vary in the cognitive abilities examined (general intellectual score vs. ability-specific scores). Second, they adopt different statistical approaches to operationalize cognitive change. Such variations have hindered attempts at summarizing the effects of cognitive change on survival (Aichele et al., 2021; Bäckman & MacDonald, 2006).

### Cognition-survival relations toward the end of the life span

In old and very old age, cognition becomes particularly salient for daily life functioning, and higher general cognitive abilities have been shown to predict longer survival times (e.g., Bäckman & MacDonald, 2006; Bosworth & Siegler, 2002). Two hypotheses have received particular attention in this context, that of *terminal decline*, which emphasizes gradual, linear losses in cognitive performance toward the end of life (Palmore & Cleveland, 1976), and *terminal drop*, which indicates precipitous, curvilinear decline in intelligence shortly before death (Kleemeier, 1962; Riegel & Riegel, 1972). Although terminal decline and terminal drop are not always explicitly distinguished, there are theoretical and clinical reasons to differentiate them (Berg, 1996). Currently, empirical evidence appears stronger in favor of terminal decline, rather than drop (MacDonald et al., 2011).

However, whether specific cognitive abilities (and changes therein) differentially relate to mortality remains an open question. Broad crystallized-pragmatic abilities (Gc; e.g., vocabulary) and broad fluid-mechanic abilities (Gf; e.g., perceptual speed) have been differentiated and frequently examined in this context. Gc is typically more age-resistant than Gf, and its decline may reflect disease progression during the period of terminal drop, when pathological processes become manifest (Birren, 1965; White & Cunningham, 1988). In contrast, Gf is more sensitive to normal cognitive aging, and its decline may reflect longer-term changes in the integrity of the central nervous system (Birren & Fisher, 1995), such as increasing cerebral white-matter lesion burden (Hillary et al., 2021), during terminal decline. The evidence in favor of survival associations with Gc appears quite weak, whereas it is mixed for Gf (Bäckman & MacDonald, 2006). However, individual differences in changes in Gf and in Gc covary markedly, indicating that overlapping (biological or cognitive) processes and sets of risks hampering brain maintenance may affect both (Ghisletta et al., 2012; Nyberg & Lindenberger, 2020; Tucker-Drob et al., 2022). Thus, analyzing these processes concurrently can help distinguish their unique predictive contributions. In addition, studies are often inconsistent in their definitions and assessments of cognitive abilities, relying on different, at times noncomparable, indicators. Single intelligence scores indicating overall cognitive performance may vary widely in their measurements, depending on the type of assessment used (i.e., ranging from a large, psychometric cognitive battery to a neuropsychological screening test such as the Mini-Mental State Examination, or MMSE; Folstein et al., 1983).

### Methodologies to study cognition-survival relations

Extant literature reviews on cognition-survival relations have summarized methodological considerations at length (e.g., Aichele et al., 2021; Bäckman & MacDonald, 2006; Ghisletta et al., 2006). The limitations of using cross-sectional (single-assessment) designs rather than repeated-measures designs are well established. It is relevant to note that only the latter allow researchers to test how changes, and not just baseline performance, in cognitive ability relate to mortality. Even when researchers possess repeated-measures data, the specific analytical approach taken can make a notable difference when estimating longitudinal-survival associations, for which nonrandom attrition due to death cannot be ignored.

One consideration in longitudinal-survival modeling is whether to use a two-stage estimation process or a simultaneous (i.e., joint) estimation process (Aichele et al., 2021). The former first applies a longitudinal model to the repeated cognitive assessments to estimate individual characteristics (e.g., level and change scores). These scores are subsequently included in a survival model to test their relation to the risk of death. In contrast, joint longitudinal-survival models (JLSMs) allow for the simultaneous estimation of cognitive change and cognition-mortality associations.

Although this distinction may appear as a technical triviality, the consequences in terms of statistical precision and bias may be considerable. Separate estimation of the longitudinal and the survival models artificially implies the independence of the two processes, though the two are mechanistically related, insofar as early decedents likely show less advantageous cognitive trajectories prior to death than longer-term survivors. Thus, the cognitive trajectories of older adults not conditioned on differences in survival status are positively biased. Similarly, information about cognitive decline can inform the survival process, given that individuals with steeper declines are more likely to die sooner (also conflating mortality risk with attrition). Joint estimation addresses the inherent dependency between longitudinal and survival processes and thereby provides a more realistic account of their association. In particular, whereas traditional longitudinal models rely on the missing-at-random assumption, joint modeling more reasonably assumes that data are missing partially because of (imminent) death, thereby reducing estimation bias.

At present, few studies have used joint estimation to investigate longitudinal cognition-mortality associations. Of these, Muniz Terrera et al. (2011) found that lower memory performance and increased change in rate of memory decline predicted shorter survival time. Likewise, Muniz-Terrera et al. (2018) found that higher visuospatial ability values forecast longer survival. Sensitivity analyses in both studies showed comparatively less bias using joint estimation, owing to its reduced reliance on the missing-at-random assumption. Batterham et al. (2012) found that level in speed and in verbal fluency and change in the MMSE predicted survival, whereas memory and vocabulary did not.

Several studies have directly compared joint estimation and two-stage estimation procedures to assess potential differences in bias and efficiency. Ghisletta et al. (2006) found that under joint estimation, level of speed and verbal fluency predicted survival, whereas two-stage estimation failed to detect cognition-survival associations. Aichele et al. (2021) found more salient predictive effects using a joint approach, notably for Gc. In a different application, McArdle et al. (2005) found that, for both joint and two-stage procedures, level of (but not change in) memory predicted onset of Alzheimer’s disease in a sample of older adults. They, too, found the joint procedure to be more efficient (i.e., smaller standard errors) than two-stage estimation. Finally, only Ghisletta (2008) used a multivariate joint model to investigate speed and fluency as predictive of survival. Only level of fluency was found to forecast mortality.

### Purpose of the present study

Here, we examined performance trajectories for nine tasks targeting four cognitive abilities, and a composite measure of general intelligence, as predictors of mortality risk. We analyzed data from a sample of old and very old individuals from the Berlin Aging Study (Baltes & Mayer, 1999), who were assessed repeatedly from four to 14 times (across eight waves), all deceased by the time of analysis. Cognitive trajectories and their associations to mortality were estimated simultaneously using a joint multivariate longitudinal and survival model (JMLSM; Rizopoulos, 2012). The cognitive trajectories were characterized dynamically at any given time point so that an individual’s current true (latent, error-free) score and slope (i.e., first derivative) were used for mortality prediction. In brief, we used state-of-the-art longitudinal-survival modeling to compare cognition-mortality associations across multiple domain-specific abilities.

## Research Transparency Statement

### General disclosures

**Conflicts of interest**: All authors declare no conflicts of interest. **Funding**: Financial support came from the Max Planck Society; the Free University of Berlin; the German Federal Ministry for Research and Technology (1989–1991, Grant Nos. 13 TA 011 and 13 TA 011/A); the German Federal Ministry for Family, Senior Citizens, Women, and Youth (1992–1998, Grant Nos. 314-1722-102/9 and 314-1722-102/9a); and the Berlin-Brandenburg Academy of Sciences’ Research Group on Aging and Societal Development (1994–1999). **Artificial intelligence**: No artificial-intelligence-assisted technologies were used in this research or in the creation of this article. **Ethics**: Ethics approval for the Berlin Aging Study was granted by the Berlin Medical Association.

### Study disclosures

**Preregistration**: No aspects of the study were preregistered. **Materials**: The materials (cognitive tasks) are not publicly available because of a restrictive license and technological obsolescence. Paper versions of the tasks are available upon request from the Berlin Aging Study (https://www.base-berlin.mpg.de/en). Further details for accessing the materials are available at https://osf.io/g23vu. **Data**: The primary data are not publicly available because of participant privacy and consent concerns; however, the data are available upon request from the Berlin Aging Study (https://www.base-berlin.mpg.de/en). Further details for accessing the data are available at https://osf.io/e5ucq. **Analysis scripts**: All analysis scripts are publicly available (https://osf.io/u57gr/files/osfstorage). **Computational reproducibility**: The computational reproducibility of the results has not been independently confirmed by the journal’s STAR team because the data are not available.

## Method

### Sample

The Berlin Aging Study (Baltes & Mayer, 1999) is a comprehensive and intensive interdisciplinary aging study involving psychology; sociology and social policy; psychiatry; and internal and geriatric medicine. Ethical approval for the Berlin Aging Study was granted by the Berlin Medical Association. Data collection started in 1990, in the former West Germany, with a register-based sample of West Berlin. Eight waves were collected up to 2008–2009. Mean study duration across all participants was 4.04 years (maximum duration = 18.36 years). By now, all participants have passed away, and their exact dates of death are known. For design reasons (equal statistical power), the original sample was stratified by age (70–74, 75–79, 80–84, 85–89, 90–94, and 95 years and older; *n =* 86 in each stratum) and sex (258 males and 258 females; each of the 12 cells contained *n =* 43 participants). All analyses considered age as a continuous variable.

Each wave of assessment but the second (for funding reasons) comprised an initial assessment (IA) and an intensive protocol (IPr). The IA was interdisciplinary and included only a few indicators from each study domain. The IPr sessions were discipline specific and were composed of more comprehensive testing protocols, such as a comprehensive cognitive battery (Lindenberger et al., 1993). Thus, at each wave most tasks were assessed once (at IPr), but a few tasks were assessed twice (at IA and IPr). The longitudinal Berlin Aging Study cognitive battery included tasks of perceptual speed, episodic memory, verbal fluency, and verbal knowledge.

### Instruments

The analyses presented here included data from all Berlin Aging Study cognitive tasks that were administered longitudinally and repeatedly between four and 13 times. Table 1 summarizes the cognitive variables across Berlin Aging Study Waves 1 to 8, accompanied by sample summary information. Average distance between waves was approximately 2 years and 5 months; within any wave (but the second), average distance between IA and IPr was about 53 days. Participants were assessed individually in their homes, in a quiet setting, by trained research assistants. Each test was preceded by practice sessions. Testing time was either limited (e.g., 90 s) or ended upon three consecutive failures. All cognitive tests are described in detail in Lindenberger et al. (1993). For all tests, the scores analyzed were the total correct answers, transformed to T metric (*M =* 50, *SD =* 10; anchored at the first intensive protocol, IPr_1_) for consistent scaling across measures.

**Table 1.** Berlin Aging Study Design Limited to Variables Analyzed

Years of testing	1990–1993		1993–1994		1995–1996		1997–1998		2000		2004		2005		2008–2009	
Testing occasion	1		2		3		4		5		6		7		8	
Testing session	IA	IPr	IA	IPr	IA	IPr	IA	IPr	IA	IPr	IA	IPr	IA	IPr	IA	IPr
Perceptual speed	DL	DS, DL, IP	DL	—	DL	DL, IP	DL	DL, IP	DL	DL, IP	DL	DL, IP	DS, DL	DS, DL	DS	—
Episodic memory	—	PA, MT	—	—	—	PA, MT	—	PA, MT	—	PA, MT	—	PA, MT	—	—	—	—
Verbal fluency	—	CA, WB	CA	—	CA	CA, WB	CA	CA, WB	CA	CA, WB	CA	CA, WB	CA, WB	CA, WB	—	—
Verbal knowledge	—	VO, SW	—	—	—	VO, SW	—	VO, SW	—	VO, SW	—	VO, SW	—	—	VO	—
G	—	yes	—	—	—	yes	—	yes	—	yes	—	yes	—	—	—	—
Dementia	yes	—	yes	—	—	yes	—	yes	—	yes	—	yes	—	—	—	—
*N*	516	516	361	—	244	208	164	132	88	82	48	47	38	38	22	22
Time in study	0.00 (0.00)	0.13 (0.09)	1.95 (0.71)	—	3.76 (0.66)	3.99 (0.69)	5.53 (0.79)	6.03 (0.80)	8.94 (0.84)	9.00 (0.86)	13.00 (0.87)	13.04 (0.88)	13.87 (0.91)	13.88 (0.91)	16.93 (0.87)	16.98 (0.88)
Chronological age	84.92 (8.66)	85.04 (8.68)	85.26 (8.41)	—	84.34 (7.30)	83.87 (6.91)	84.07 (6.33)	84.30 (5.90)	85.87 (4.36)	85.86 (4.48)	89.36 (4.58)	89.47 (4.60)	90.08 (4.72)	90.10 (4.72)	92.24 (4.49)	92.29 (4.51)

Note: Time in study is in years (*M* and *SD*); chronological age is in years (*M* and *SD*). IA = initial assessment; IPr = intensive protocol; Dementia = occasion–specific assessment of suspected dementia (0 = *no*, 1 = *yes*); DS = digit symbol; DL = digit letter; IP = identical pictures; PA = paired associates; MT = memory for text; CA = categories; WB = word beginnings; VO = vocabulary; SW = spot-a-word; G = general intelligence score.

#### Perceptual speed tasks

##### Digit symbol

This test comes from the Wechsler battery (Wechsler, 1955) and presented participants with a template of digits from 1 to 9, each associated with a symbol, followed by 90 digits, which participants had to pair with the correct symbol within 90 s. The test was introduced on a computer screen, but actual testing took place using the usual paper-and-pencil format, with the test sheet enlarged by 100% to reduce perceptual and motor problems.

##### Digit letter

This test consisted of 21 sheets, on top of which was a template with digits from 1 to 9, each associated with a letter, printed in Times Roman Bold size 48. Each sheet contained six digits, and the research assistant presented the next sheet as soon as participants finished a sheet. Participants were asked to name as many corresponding letters as possible within 180 s.

##### Identical pictures

This test was a computerized version of the homonymous test from the Educational Testing Service (Ekstrom et al., 1976). Thirty-two items were presented, each of which consisted of a target figure in the upper half of the screen and five possible responses in the lower half. Participants were asked to choose an answer as quickly as possible within 80 s.

#### Episodic memory tasks

##### Paired associates

A list of eight pairs of real nouns was shown twice, each pair for 5 s. After the presentation, only the first noun of each pair was presented, in a different order. Participants had to recall the second noun, and their answers were subsequently rated for correctness by two independent research assistants.

##### Memory for text

This test was adapted from Engel and Satzger (1990). Participants were presented with a short story, both visually and auditorily, lasting about 38 s. Immediately thereafter, six questions about the story were asked, and the answers were rated for correctness by two independent research assistants.

#### Verbal-fluency tasks

##### Categories

Participants had to name as many different animals as possible within 90 s. Their answers were subsequently rated for correctness by two independent research assistants, to assure that noticed or unnoticed repetitions, wrong categories, and morphological variants were not coded as correct.

##### Word beginnings

Participants were asked to name as many different real words starting with the letter *s* as possible within 90 s. The named words were rated for correctness by two independent research assistants to avoid considering repetitions, morphological variants, and wrong words as correct.

#### Verbal knowledge tasks

##### Vocabulary

Twenty words selected from the Vocabulary test of the German version of the Wechsler Adult Intelligence Scale (WAIS; Wechsler, 1982) were presented one at a time on the screen. Participants had to define each word, and their answers were coded for correctness (total, partial, wrong) by two independent research assistants. There was no time limit.

##### Spot-a-word

Participants were presented with 20 series of four pronounceable nonwords mixed with a single real word from a German vocabulary test (Lehrl, 1977). Participants were asked to select the real word from each series, without time limits.

#### General intelligence score G

For comparison purposes, we also calculated a composite score of all cognitive tasks, when administered, to estimate a score of general intelligence, G. This was calculated as the average of all task scores in their T metric and expressed in the same metric (the mean score correlated *r* = .98 with an estimated factor scores from a one-factor model; cf. Appendix 1 in the Supplemental Material available online). For replicability purposes, we chose to compute G as the mean score, which would be computed equivalently in a different sample, rather than as an estimated factor score, which would be computed differently in a different sample (i.e., because of different factor loadings; cf. Widaman & Revelle, 2023).

#### Sociobiographical status

This variable combined (as a unit-weighted composite of standardized scores) the net income in Deutsche Marks on a 5-point scale (1 = less than DM1,000, 2 = DM1,000–1,399, 3 = DM1,400–1,799, 4 = DM1,800–2,100, 5 = DM2,200 and above), occupational prestige (following a standard scale in Germany, ranging from 22.7 to 186.8, *M* = 79.6, *SD* = 32.2), social class on a 4-point scale (with 7% considered lower class, 20% lower middle class, 31% middle class, and 30% upper middle class), and number of years of formal education (counting elementary and high school, but also professional and academic training; *M* = 10.8 years, *SD* = 2.3 years), all based on standard German scales (Mayer et al., 1999). We transformed the composite to T metric, with mean centering (*M =* 0, *SD =* 10, min = −21.55, max = 30.24). For further details, see Lindenberger and Baltes (1997).

#### Suspected dementia

Clinical diagnoses of dementia at IPr_1_ and IPr_3_ were used to validate the application of age-cohort specific cutoff scores based on the Short MMSE (Klein et al., 1985; less than 12 points for 70–84 years of age, less than 11 points for 85 years of age and older) with sufficient specificity (72%–98%) and sensitivity (63%–88%; Gerstorf et al., 2006). This indicator is available in the Berlin Aging Study up to the sixth wave (included).

### Statistical analyses

The JMLSM is defined by its two submodels, the multivariate longitudinal component and the survival component (Henderson et al., 2000). The main feature consists in the association elements that are defined in the former submodel and entered as covariates in the latter. Because the overall joint model estimates simultaneously all elements from both submodels, the longitudinal and survival parameters are statistically conditioned upon each other. Of relevance here, the cognitive trajectories are corrected for individual differences in timing and risk of death, and the risk of death is expressed as a function of the past cognitive trajectories. This overcomes the limits of the missing-at-random assumption.

#### Multivariate longitudinal submodel

We used the multivariate mixed-effects model (MMEM) for the multivariate longitudinal submodel of the JMLSM. The MMEM adds fixed and random effects to errors to characterize participants’ repeated assessments (Laird & Ware, 1982). We relied on the notation of Rizopoulos (2012) and represented the MMEM as:

(1) $$y_{iq}(t)=m_{iq}(t)+\varepsilon_{iq}(t)\;and\;m_{iq}(t)={x^{⊤}}_{iq}(t)\beta +{z^{⊤}}_{iq}(t)b_{iq},$$

where ***y**_iq_* (*t*) is the vector of observed values of the *q*^th^ outcome (the nine cognitive tests) of participant *i* at time *t* and is assumed to be the sum of the time-specific true, unobserved trajectory ***m**_iq_* (*t*) and the error component **ε**_*iq*_(*t*) at time *t*. Thus, ***y**_iq_* (*t*) differs from ***m**_iq_* (*t*) in that the former is contaminated by the measurement error **ε**_*iq*_(*t*). The true trajectory is specified as the sum of the sample fixed effects **β**, weighted by the participants’ values on the covariates ***x*** relevant for the *q*^th^ outcome, and the participant-specific random effects ***b*** for that *q*th outcome, weighted by the covariates ***z*** (which may or not differ from ***x***). The random effects ***b**_iq_* ~ *N* (0, *D*), that is, are assumed to be normally distributed, centered at zero, with covariance matrix D , which is often specified as unstructured. This means that the random effects for a given outcome *q* can correlate with those of another outcome *q′*, which is not possible in a univariate setup (i.e., when a single outcome *q* is analyzed by itself). The errors **ε**_*iq*_(*t*) ~ *N* (0, σ_*q*_^2^), that is, are assumed to be normally distributed, centered at zero, with variance σ_*q*_^2^. The errors are assumed uncorrelated with the random effects.

A technical prerequisite of the JMLSM is that the timing variable *t* be the same in both longitudinal and survival submodels (Rizopoulos, 2012). We thus defined the cognitive trajectories over time in the study—that is, years of participation within the Berlin Aging Study (difference between age at testing and age at the first wave), measured in years and rounded to two decimals (as in Lindenberger & Ghisletta, 2009). We defined time squared as quadratic terms of time residualized on linear time effects, to avoid collinearity between the two (as in Lindenberger & Ghisletta).

We first tested each cognitive outcome separately, in a series of univariate mixed-effects models, to provide the specification that best fitted each outcome to the subsequent multivariate model. For each cognitive outcome, we tested occasion-specific retest effects, defined as dummy variables with values of 1 or 0 when an individual was or was not assessed at that occasion, respectively. These retest predictors thus marked the occasion at which a score was obtained and are included in the model together with time in study. Whereas time in study assessed the effect of aging on a cognitive score (as a continuous variable counting years passed since the first IA), the retest predictors assessed the separate effect, due to previous test exposure, of taking the cognitive task at a given occasion. Testing both time in study and occasion-specific retest effects was possible because they correlated weakly (on average *r* = .15, ranging from *r* = −.40 to *r* = .31). Whenever retest estimates were negative, we omitted them from the model because they mimicked time rather than retest effects (for further details, see Ghisletta et al., 2006, 2014). We compared different specifications of the random effects (matrix D of ***b**_i_* in Equation 1), testing separately for variance of time and time squared. We also tested the interaction between initial age (analyzed continuously at IA_1_) and time and, whenever different from zero, we retained it in the subsequent multivariate model. For each cognitive outcome, we performed likelihood ratio tests to select the model specification that best described the data.

#### Survival submodel

The focal event was death, which marks the unequivocal and nonrecoverable exit from the previous state of being alive (i.e., a single-spell event). We defined the time at risk as starting at the beginning of the Berlin Aging Study, so that we used time in study, measured in years and rounded to two decimals, as our timescale (Kalbfleisch & Prentice, 2002). We used the Cox regression model (Cox, 1972), which is extremely popular because it allows testing the relative effect of predictors on the (log of) the hazard of the event and because it approximates rather well estimates from various known parametric models (e.g., exponential, Weibull, Gompertz) in many empirical situations (Yamaguchi, 1991). The Cox model postulates a completely general baseline hazard function *h_0_*(*t*), which does not need to be estimated to assess and interpret the effects of the predictors.

By borrowing the notation of Rizopoulos (2012) we represent the Cox regression model as

(2) $$h_{i}(t|M_{iq}(t),w_{i})=h_{0}(t)exp\{\gamma^{⊤}w_{i}+\sum_{q}\alpha_{q}m_{iq}(t)\}$$

—where the relative risk *h* of an event (here death) for individual *i* at time *t* is expressed as a function of $M_{iq}(t)$ , the history of the true unobserved longitudinal processes *q* (here cognitive performance on the nine Berlin Aging Study tests and on G) up to time *t*, and of additional covariates ***w**_i_* (here initial age [analyzed continuously], sex, sociobiographical status, suspected dementia). The baseline risk function is *h_0_*(*t*), ***γ*** is the vector of survival regression weights of the covariates ***w**_i_*, and **α**_*q*_ expresses the effect of features of the true longitudinal trajectories ***m**_iq_* (*t*) on the risk of death. Thus, the previous cognitive trajectories enter the survival submodel free of their error of measurement, which would not be possible if the cognitive scores were entered as time-varying predictors in an extended Cox model. The effects of the *q*^th^ outcome are additive, so that their true longitudinal trajectories influence the risk of the event of interest while also controlling for each other’s effect.

#### JMLSM

The final JMLSM explicitly defines which features of the longitudinal trajectories enter as covariates in the survival submodel. The parameters **α**_*q*_ in the survival submodel represent here the association between the error-free component of the longitudinal cognitive process assessed for each cognitive test ( $M_{iq}(t)$ ) and the risk of death (*h_i_*(*t*)), as follows—

(3) $$h_{i}(t|M_{iq}(t),w_{i})=h_{0}(t)exp\{\gamma^{⊤}w_{i}+\;\sum_{q}\left[\alpha_{1q}m_{iq}(t)+\alpha_{2q}m\prime_{iq}(t)\right]\}$$

—where ***m*** ′_*iq*_(*t*) = (*d **m**_iq_*(*t*)) / *dt* (that is, the first derivative with respect to *t* of the true, unobserved longitudinal trajectory of the *q*^th^ outcome; **α**_2_ is its associated survival regression weight). Whereas ***m**_iq_* (*t*) represents the current value of subject *i* at time *t* on their true trajectory of the *q*^th^ cognitive outcome, its first derivative (***m*** ′_*iq*_(*t*)) defines the current (instantaneous) rate of change of that subject at that same time on that outcome. Thus, both the current value and the current rate of change of that value for any subject, at any time, for all cognitive outcomes can independently influence the hazard of dying. In the end, two association parameters per cognitive outcome *q* are estimated: **α**_1_, which expresses the effect of the current value of that cognitive outcome at time *t* on the risk of death, and **α**_2_, which expresses the effect of the current rate of change of that value at time *t* on the risk of death.

By considering both current value and current rate of change, the model distinguishes, for instance, two individuals, *i* and *i*′, who at a given time *t* have the same value on the same outcome *q* (***m**_iq_* (*t*) = ***m**_i′q_* (*t*)), but who are on different trajectories, in which one may be declining and the other increasing (***m**′_iq_*(*t*) < ***m**′_i′q_*(*t*)). We hypothesized that, for those cognitive outcomes associated with the hazard of death, a greater current value and a greater rate of change (i.e., a less steep rate of decline) ought to decrease the hazard of death. Thus, we hypothesized that **α**_1*q*_ < 0 and **α**_2*q*_ < 0.

To estimate the JMLSM, we used the R language and environment, version 4.2.3 (R Core Team, 2023) and its package *JMbayes2*, version 0.4-0, which uses Markov chain Monte Carlo algorithms implemented in C++ under the Bayesian approach (Rizopoulos et al., 2023). To obtain stable Bayesian estimates, we specified three chains, each with 100,000 iterations, with 20,000 burn-in iterations and a thinning of 5, thus yielding 16,000 estimated parameter values per chain (cf. https://osf.io/u57gr/files/osfstorage for the R syntax). On a powerful computer (32 GB RAM memory and 24 parallel batches), the final model took over 30 hrs of running time. The *JMbayes2* package fits a JMLSM by starting from multiple univariate mixed-effects models fitted with the *nlme* package (Pinheiro & Bates, 2023), for which we used version 3.1-162, and a survival model fitted with the *survival* package (Therneau, 2023), for which we used version 3.5-3. Because our mixed-effects models were linear in their parameters, both univariate longitudinal and survival models were first estimated with maximum likelihood, and their solutions were fed to the Bayesian estimation procedure of the JMLSM.

#### Additional sensitivity analyses

##### Joint univariate versus multivariate longitudinal survival models

Because much of the extant literature on cognition-survival relationships is based on independent analyses of single cognitive tasks, we tested 10 univariate JLSMs, one per cognitive task, plus one for the general intelligence score, G. These analyses allow comparing shared predictive effects (from the JLSMs) to unique predictive effects (from the JMLSM). That is, if the longitudinal trajectory of a cognitive task predicts survival in the multivariate model, its effect emerges above and beyond the effects of all other tasks.

##### Joint bivariate longitudinal survival models with G

To ascertain the unique predictive potential of the single cognitive tasks against G, we tested each in a series of joint bivariate longitudinal survival models (JBLSMs), in which the second longitudinal predictor of survival was G. This set of analyses provides a strong test of the specificity of each cognitive task vis-à-vis a general intelligence score G (composed by each task, in equal parts).

##### Joint versus two-stage estimation

Extant longitudinal investigations of cognition-survival relationships have often used a two-stage approach, in which individuals’ cognitive trajectory scores are first estimated in longitudinal models and then included as predictors in subsequent survival models. We therefore conducted supplemental two-stage analyses for both comparative and confirmatory purposes. For each cognitive task, we first computed a mixed-effects longitudinal model (ignoring survival status), from which we estimated current values and current rate of change values across all individuals (estimated ***m**_i_* (*t*) and ***m*** ′_*i*_(*t*) scores; see the detailed description and results in Appendix 8 in the Supplemental Material). We then added these two sets of scores for all cognitive tasks in a multivariate Cox survival regression model (controlling for initial age, sex, sociobiographical status, and suspected dementia). We also applied the two-stage estimation procedure in a series of 10 univariate survival models, where each of the nine cognitive tasks and G were separately tested (controlling for initial age, sex, sociobiographical status, and suspected dementia). This analysis compared directly the statistical efficiency of a joint compared to a two-stage estimation procedure.

##### Sociobiographical status

Because crystallized abilities can be fostered by higher sociobiographical status, we ran a series of Cox survival regression analyses specifically addressing the potential predictive effects of the four variables (net income, occupational prestige, social class, and number of years of formal education) composing the sociobiographical composite score. These are presented in Appendix 9 in the Supplemental Material.

## Results

For brevity, we here limit our focus to the results of primary importance, namely, those from the survival submodel of the JMLSM and from the relevant follow-up sensitivity analyses. Results from the preliminary longitudinal analyses of the univariate cognitive scores and from the preliminary survival analysis (which ignores cognitive trajectories) are available in the Supplemental Material (see Tables S1 and S2 and Appendix 2 for detailed residual analyses). Results from the multivariate longitudinal submodel of the JMLSM are presented in Supplemental Table S3 and discussed in Appendix 3, also in the Supplemental Material. The JMLSM Bayesian estimation algorithm converged very well, as reported in Appendix 4 and in Supplemental Figures S1, S2, and S3 (Markov chain Monte Carlo diagnostics). Thus, we interpret results based on 16,000 quantiles with confidence.

### Survival submodel from the JMLSM

The parameter estimates from the survival submodel of the JMLSM are presented in Table 2 in terms of the means of their Bayesian posterior distributions and 95% credible intervals (CIs; defined between the 2.5^th^ and 97.5^th^ percentiles). Initial age was positively associated with mortality risk: an additional year in age was associated with a 7.82% increase, 95% CI = [5.33%, 10.48%]. Women had a 31.51% decreased mortality risk (95% CI = [14.55%, 44.25%]). Sociobiographical status and suspected dementia did not reliably influence survival. Compared to the stand-alone survival results (cf. Table S2 in the Supplemental Material), these parameter estimates were confirmed, except for suspected dementia, which, when previous cognitive trajectories were not included in the survival model, increased the hazard of death by 40.39% (95% CI = [15.57%, 71.23%]). The JMLSM included cognitive trajectories, and the variance in these trajectories can likely be partly explained by suspected dementia, which in turn clouds the effect of the latter on survival (as in Muniz-Terrera et al., 2018).

**Table 2.** Parameter Estimates and 95% Credible Intervals from the Survival Submodel of the Final Joint Multivariate Longitudinal Survival Model (*N* = 516)

Cognitive domain	Predictor	Longitudinal characteristic	Estimate	Hazard ratio
	Initial age		0.08 [0.05, 0.10]	1.08 [1.05, 1.10]
	Sex		–0.36 [–0.58, –0.15]	0.69 [0.56, 0.86]
	Socio-biographical status		0.01 [–0.01, 0.02]	1.01 [1.00, 1.02]
	Dementia		0.07 [–0.17, 0.31]	1.07 [0.84, 1.36]
Perceptual speed	Digit symbol	α_1_	–0.01 [–0.03, 0.02]	1.00 [0.97, 1.03]
		α_2_	–0.01 [–0.01, 0.01]	1.00 [1.00, 1.00]
	Digit letter	α_1_	–0.01 [–0.01, 0.01]	0.99 [0.96, 1.02]
		α_2_	0.01 [–0.01, 0.01]	1.00 [1.00, 1.01]
	Identical pictures	α_1_	0.01 [–0.01, 0.01]	1.00 [0.97, 1.03]
		α_2_	0.01 [–0.01, 0.01]	1.00 [1.00, 1.00]
Episodic memory	Paired associates	α_1_	–0.01 [–0.04, 0.01]	0.98 [0.96, 1.01]
		α_2_	0.01 [–0.02, 0.03]	1.01 [0.98, 1.03]
	Memory for text	α_1_	0.03 [–0.01, 0.06]	1.03 [0.99, 1.07]
		α_2_	–0.01 [–0.03, 0.02]	0.99 [0.97, 1.02]
Verbal fluency	Categories	α_1_	–0.05 [–0.08, –0.03]	0.95 [0.92, 0.97]
		α_2_	0.01 [–0.01, 0.01]	1.00 [1.00, 1.00]
	Word beginnings	α_1_	–0.03 [–0.05, –0.01]	0.97 [0.95, 0.99]
		α_2_	0.01 [–0.01, 0.01]	1.00 [1.00, 1.01]
Verbal knowledge	Vocabulary	α_1_	0.03 [–0.01, 0.06]	1.03 [1.00, 1.06]
		α_2_	0.01 [–0.01, 0.01]	1.00 [1.00, 1.01]
	Spot-a-word	α_1_	–0.01 [–0.04, 0.03]	1.00 [0.97, 1.03]
		α_2_	–0.01 [–0.01, 0.01]	1.00 [0.99, 1.01]

Note: Initial age is in years (at study inception); sex is coded 0 = men, 1 = women; dementia = occasion-specific assessment of suspected dementia (0 = no, 1 = yes); α_1_ = survival effect of current value; α_2_ = survival effect of current rate-of-change value; Underlined numbers correspond to effects with a 95% credible interval that does not include 0 (non-null effects). All 516 participants died.

Of the cognitive markers, only the current values (α_1_) of both verbal-fluency markers (category and word beginning) were related to the risk of death. A one-unit increase in the current value of category fluency predicted a 5% (95% CI = [3%, 8%]) reduction in mortality risk. A one-unit increase in the current value of word-beginning fluency predicted a 3% (95% CI = [1%, 5%]) reduction in mortality risk. These effects translate to a reduction in risk of dying of 5.6% for an additional animal (category) and of 3.7% for an additional word starting with “s” (word beginning) named by a participant.

Sensitivity analyses of misspecified JMLSM—omitting either category or word-beginning features ***m**_i_* (*t*), ***m*** ′_*i*_(*t*), or both—showed that (a) the current value and current rate-of-change parameters were not collinear and (b) the current value effects of category and word beginnings on survival did not overlap substantially (cf. Appendix 5 in the Supplemental Material).

**Fig. 1.**
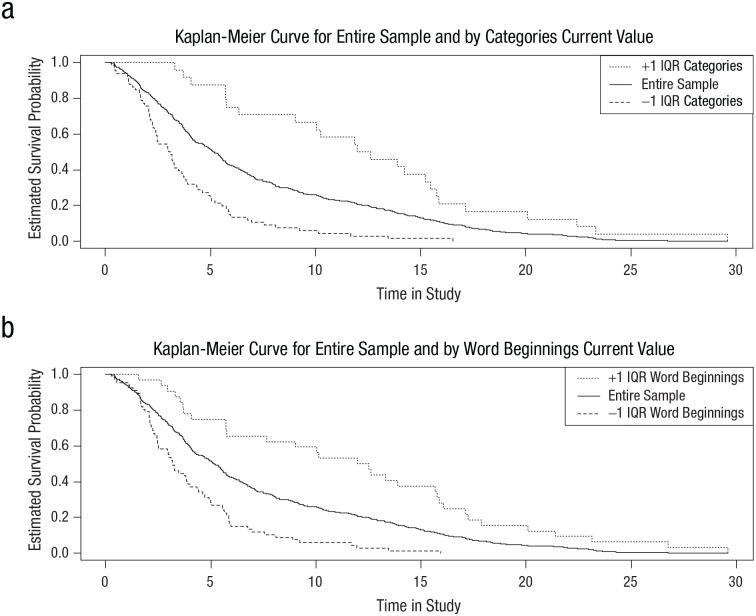
Estimated Survival Probabilities (Kaplan-Meier Method) for the Entire Sample and by Categories and Word Beginnings. Estimated survival probabilities (Kaplan-Meier method) for individuals with average current value on (a) categories or (b) word beginnings. The upper dotted line represents 1 interquartile range above the median, and the lower dashed line represents 1 interquartile range below the median; the middle, continuous line is for the entire sample. IQR = interquartile range.

To further aid interpretation, we categorized participants as having high or low (±1 interquartile range, or IQR, from the median) average current values for both category and word beginnings—estimated ***m**_iCA_* (*t*) and ***m**_iWB_* (*t*)—across the study window. For both groups and for the entire sample we plotted the estimated survival probabilities via the Kaplan-Meier method (Kaplan & Meier, 1958) in Figure 1. The commonly used reference *median survival time*, equivalent to the time value with 0.5 estimated survival probability, was 5.17 years for the entire sample (see the middle continuous line in both panels of Fig. 1), 3.01 years for those with low category values (about 11 animals), and 11.99 years for those with high category values (about 33 animals; see lower dashed line and upper dotted line, respectively, in Fig. 1a). For word beginnings, the low and high median survival times (for about 7 and 22 words starting with “s”) were estimated at 3.18 and 11.99 years (Fig. 1b), respectively.^1^ This suggests that there is an almost 9-year predicted difference in median survival time between Berlin Aging Study participants with high versus low values on verbal fluency.

### Joint univariate versus multivariate longitudinal survival models

The predictive effects for current values (α_1_) and current rate-of-change values (α_2_) of the nine cognitive tasks and of G are reported and described in Appendix 6 in the Supplemental Material. In short, current values for G and for all tasks, except for spot-a-word (assessing verbal knowledge), predicted survival. Rates of change were, however, not predictive. The strongest estimate was for category (α_1_ = −0.053, 95% CI = [–0.068, –0.038]), followed by G (α_1_ = −0.048, 95% CI = [–0.064, –0.033]) and word beginnings (α_1_ = −0.046, 95% CI = [–0.063, –0.030]), confirming the importance of the fluency tasks in predicting mortality. Moreover, these outcomes make it very clear that although nearly all tasks were predictive of survival when considered alone, when analyzed together, only fluency tasks had predictive influence above and beyond all other tasks.

### Joint bivariate longitudinal survival models with G

The effects for current values (α_1_) and current rate of change values (α_2_) for each task (in the columns, across rows 1 and 2) and for G (on rows 3 and 4) estimated from nine joint bivariate longitudinal survival models (each with G) are reported and further described in the Supplemental Material (Appendix 7). Again, in no case were rate-of-change values predictive of survival. Across analyses, current values of G were consistently predictive, except when tested alongside the two fluency tasks—category and word beginnings. The bivariate model with category and G obtained that only the former predicted death (α_1_ for category = −0.042, 95% CI = [–0.071, –0.014]), but not the latter (α_1_ for G = −0.014, 95% CI = [–0.044, 0.016]). The bivariate model with word beginnings and G obtained that neither predicted death.

### Joint versus two-stage estimation

Appendix 8 in the Supplemental Material details the two-stage estimation procedure to analyze cognitive change and survival in a multivariate model (i.e., all cognitive variables together, controlling for each other) and in 10 univariate models (i.e., each cognitive variable analyzed separately). Estimation precision for the multivariate model was notably worse in the two-stage vs. the joint procedure (the mean and median ranges of 95% CIs were 0.039 and 0.051 for the joint estimation vs. 0.378 and 0.163 for the two-stage estimation). Yet the predictive effect of current category values again emerged in the two-stage procedure, α_1_ = −0.043, 95% CI = [–0.063, –0.023], confirming the robustness of this result.

Estimation precision was similarly worse when using two-stage estimation for the univariate models (cf. Appendix 6 for estimates from the corresponding joint models). Mean and median ranges of credible intervals increased from 0.039 and 0.051 to 0.269 and 0.281, respectively. Furthermore, the two episodic memory tasks (paired associates and memory for text) did not predict survival in the two-stage analyses. The strongest effect was again found for category (α_1_ = −0.049, 95% CI = [–0.061, –0.033]), followed by G (α_1_ = −0.039, 95% CI = [–0.052, –0.024]) and word beginnings (α_1_ = −0.035, 95% CI = [–0.050, –0.022]).

### Sociobiographical status

Results from models focusing on the effects of the sociobiographical covariates are presented and discussed in detail in Appendix 9 in the Supplemental Material. First, we tested the preliminary survival submodel (cf. Table S2), in the absence of the sociobiographical status composite score, to ascertain whether the effects of the remaining covariates would be altered. We obtained practically no differences in predictive effects for the remaining covariates: Each additional year, being a man and being diagnosed with suspected dementia increased the hazard of dying by 9.84%, 37.77%, and 42.24%, respectively. Second, we replaced the composite sociobiographical status variable in the preliminary survival submodel by its four constituent variables (net income, occupational prestige, social class, and number of years of formal education). We found that no single sociobiographical variable was predictive of survival, whereas the effects of age, sex, and suspected dementia remained similar to those obtained in the model with the sociobiographical status composite score (a 9.83%, 49.61%, and 45.74% increase in risk of dying, respectively, for an additional year in age at baseline, being male, and suspected dementia). Third, we tested a survival model with initial age (omitting it would be a gross misspecification) and the four sociobiographical variables and found that net income and occupational prestige do predict survival (once sex and suspected dementia are not part of the model). We concluded that no aspect of sociobiographical status contributed to predicting the hazard of dying above and beyond initial age, sex, and suspected dementia (although some did in the absence of sex and suspected dementia).

## Discussion

### The relevance of verbal fluency

Of the nine cognitive tasks we analyzed, we found that only the two verbal-fluency tasks predicted survival, and their estimated effects were sizeable: Participants with low (vs. high) current fluency values had a median survival time shortened by 9 years. These results appear quite robust. First, when concurrently tested against a composite measure of general intelligence, G, the fluency category (animals) task predicted survival, whereas G did not. Second, even when tested with a less-efficient two-stage estimation procedure, category fluency showed the strongest predictive effect, followed by G and the second fluency task (words beginning with the letter “s”). This suggests that once verbal-fluency scores of older persons are known, performance information related to speed, memory, and verbal knowledge, as well as general intelligence, does not enhance survival prediction.

Bäckman and MacDonald (2006) posited that verbal fluency may be the most relevant ability for investigating intelligence-survival relationships because of its hybrid nature (Lindenberger & Baltes, 1997; Shao et al., 2014). Fluency tasks require broad fluid abilities (i.e., fast information retrieval) and crystallized abilities (structure of semantic knowledge), and consequently they may be of intermediate difficulty: simple enough for survivors and late decedents, yet difficult enough for early decedents. Aside from their psychometric properties (i.e., wide range and lack of floor and ceiling effects), fluency tasks span search, word retrieval, and working memory skills that rely on efficient interactions of intact prefrontal areas with limbic areas and the cerebellum (Wagner et al., 2014). Fluency tasks have been shown to be especially sensitive to prefrontal and frontal-subcortical deficits (Cosentino et al., 2006), dementia diagnosis and progression (Karr et al., 2018), mild cognitive impairment (Belleville et al., 2017), and Parkinson’s disease (Tröster et al., 1998). The prognostic validity of fluency tasks and their sensitivity to disease progression may explain why, in the final model, suspected dementia was no longer predictive of survival, whereas it was in the stand-alone survival model when fluency scores were not controlled for.

### The benefits of joint versus two-stage estimation

This study confirms that joint longitudinal-survival estimation bolsters efficiency (i.e., smaller standard errors) and also reduces bias (i.e., estimation of conjoint and mutually conditioned predictive effects). These important advantages have previously been recognized both for the longitudinal submodel (Muniz Terrera et al. 2011; Muniz-Terrera et al., 2018) and the survival submodel (Aichele et al., 2021; Ghisletta et al., 2006) within joint analytical frameworks. Whereas two-stage estimation assumes longitudinal data to be missing at random, the joint procedure more realistically assumes that dropout may be due to imminent death. Simultaneously, survival predictors tested in the joint procedure included error-free and time-point-specific values (current and instantaneous change) of cognitive trajectories, with reduced bias and greater precision than when estimating trajectories separately using two-stage procedures.

The decision to apply joint (vs. two-stage) models may affect substantive interpretation given that some cognition-mortality associations may only emerge under joint models, which better account for potential conflation of attrition (missingness) both with cognitive decline and elevated mortality risk. A clearer picture about the survival process may emerge from a joint estimation. Predictors that appeared associated with mortality in a stand-alone survival model may no longer be deemed to impact survival directly when analyzed in a joint model where they reveal themselves to be part of the longitudinal process (a dementia diagnosis exacerbating cognitive decline, which shortens survival time).

### Study limitations

Our sample was stratified by age and sex: Men and very old individuals were overrepresented relative to the broader German population. Presently, joint models do not accommodate sampling weights that could adjust estimates for such discrepancies, although some progress has been made (e.g., Yoon et al., 2024). Nevertheless, although our sample cannot faithfully represent the German population of those birth cohorts (1887–1922), it does not differ notably from the population of individuals who were still alive when the study began (Baltes & Mayer, 1999).

Given the high general propensity of dying in old and very old age, we cannot exclude the possibility that our results were partially driven by the advanced sample average age (compared with that of some extant studies). Yet, in their review of 17 studies on cognitive decline prior to death, Karr et al. (2018) found no clear link between age and the rate of terminal decline. Nonetheless, our results warrant confirmation in population-based, and possibly also younger, samples.

## Conclusion

To our knowledge, this study is the first to utilize joint multivariate longitudinal survival modeling to examine trajectories of a broad range of cognitive abilities as predictive of mortality risk in old and very old age. Our results indicate robust and unique associations between verbal fluency and mortality risk, over and above other cognitive domains (perceptual speed, episodic memory, verbal knowledge) and general intelligence.

## Supplemental Material

sj-pdf-1-pss-10.1177_09567976241311923 – Supplemental material for Verbal Fluency Selectively Predicts Survival in Old and Very Old Age Supplemental material, sj-pdf-1-pss-10.1177_09567976241311923 for Verbal Fluency Selectively Predicts Survival in Old and Very Old Age by Paolo Ghisletta, Stephen Aichele, Denis Gerstorf, Angela Carollo and Ulman Lindenberger in Psychological Science

## Transparency

*Action Editor:* Julia Stern

*Editor:* Simine Vazire

*Author Contributions*

**Paolo Ghisletta:** Conceptualization; Data curation; Formal analysis; Investigation; Methodology; Visualization; Writing – original draft; Writing – review & editing.

**Stephen Aichele:** Conceptualization; Formal analysis; Investigation; Methodology; Validation; Visualization; Writing – review & editing.

**Denis Gerstorf:** Conceptualization; Funding acquisition; Investigation; Methodology; Project administration; Validation; Visualization; Writing – review & editing.

**Angela Carollo:** Conceptualization; Formal analysis; Investigation; Methodology; Validation; Visualization; Writing – review & editing.

**Ulman Lindenberger:** Conceptualization; Funding acquisition; Investigation; Methodology; Project administration; Validation; Visualization; Writing – review & editing.

**Artificial Intelligence:** No artificial-intelligence-assisted technologies were used in this research or in the creation of this article.

**Ethics:** Ethics approval for the Berlin Aging Study was granted by the Berlin Medical Association.

**Open Practices:** Study disclosures. Preregistration: No aspects of the study were preregistered. Materials: The materials (cognitive tasks) are not publicly available because of a restrictive license and technological obsolescence. Paper versions of the tasks are available upon request from the Berlin Aging Study (https://www.base-berlin.mpg.de/en). Further details for accessing the materials are available at https://osf.io/g23vu. Data: The primary data are not publicly available because of participant privacy and consent concerns; however, the data are available upon request from the Berlin Aging Study (https://www.base-berlin.mpg.de/en). Further details for accessing the data are available at https://osf.io/e5ucq. Analysis scripts: All analysis scripts are publicly available (https://osf.io/u57gr/files/osfstorage). Computational reproducibility: The computational reproducibility of the results has not been independently confirmed by the journal’s STAR team because the data are not available.

## Notes

1. Estimates for ±1 *SD* were extremely similar (2.97 vs. 11.85 years and 3.17 vs. 11.49 years for low vs. high category and word-beginning values, respectively; low vs. high values corresponded to about 14 vs. 30 animals for category and 9 vs. 20 words starting with “s” for word beginnings).
