# Supplementary material for: Verbal Fluency Selectively Predicts Survival in Old and Very Old Age
Source: Psychol Sci. 2025 Feb 24;36(2):87–101. doi: 10.1177/09567976241311923 (PMC13428923; doi:10.1177/09567976241311923)

Supplementary Appendix 1

Comparison of mean scores and estimated factor scores for G (cf. `1_G.R` *on* https://osf.io/u57gr/files/osfstorage*)*

We compared a composite score for G obtained as the mean across the 9 cognitive tasks at IPr$_1$ with an estimated factor score obtained from a one-factor model.

```
> BASEcogn.T1 <- BASEcogn[,c('dsip1', 'dlip1', 'ipip1', 'paip1', 'mtip1', 'caip1',
'wbip1', 'beip1', 'swip1')]
> # calculate G as the average of all cognitive tasks at IP1
> BASEcogn.T1 <- BASEcogn.T1 %>%
+   mutate(G = rowMeans(across(c(1:9)), na.rm = TRUE))
> ##########################################################################
> # estimate 1-factor model for G and estimate factor scores
> modelG <- ' FG =~ dsip1 + dlip1 + ipip1 + paip1 + mtip1 + caip1 + wbip1 + beip1 +
swip1'
> fitG <- cfa(modelG, data = BASEcogn.T1, missing = "FIML")
> BASEcogn.T1$EFSG <- lavPredict(fitG, method="regression")
```

```
> plot(BASEcogn.T1$G, BASEcogn.T1$EFSG)
```

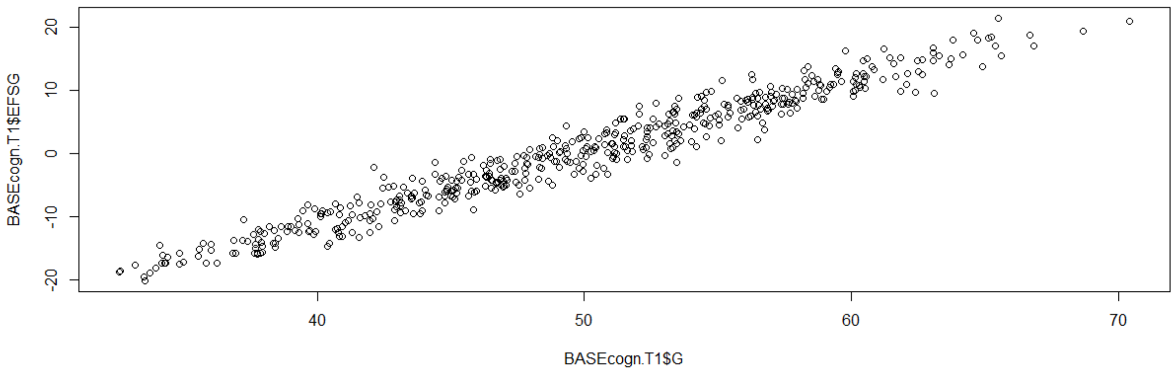


```
> cor(BASEcogn.T1$G, BASEcogn.T1$EFSG)
             FG
[1,] 0.9758784
```

Thus, the two scores for G correlated .98. For replicability purposes, we favored using the mean score (the estimated factor score would be based on loadings that will certainly differ when estimated in a different sample, so that the estimated factor score would not be fully replicable; cf. Widaman & Revelle, 2023).

Supplementary Appendix 2

Detailed description and residual analyses of the survival model

We apply a Cox regression (CR; Cox, 1972) model to estimate the hazard of death from 3 time-independent covariates: initial age (age1C; on a continuous scale, centered on its mean), socio-biographical status (sesC; on a continuous scale, centered on its mean), sex (women; 0=males, 1=females) and 1 time-dependent covariate: probable diagnosis of dementia (dementia; assessed across 6 occasions). Testing these 4 covariates requires setting up the data with the `tmerge` function of the `survival` package in `R` (Therneau, Crowson, & Atkinson, 2023) and using the same time scale for the event and for probable diagnosis of dementia: years since the study beginning, where everybody starts at 0. Hence, the time scale for the hazard of death is time in study, and not age (Kalbfleisch & Prentice, 2002: Yamaguchi, 1991). Initial age is a time-independent covariate in the model.

Estimated survival probabilities curve (Kaplan-Meier method)

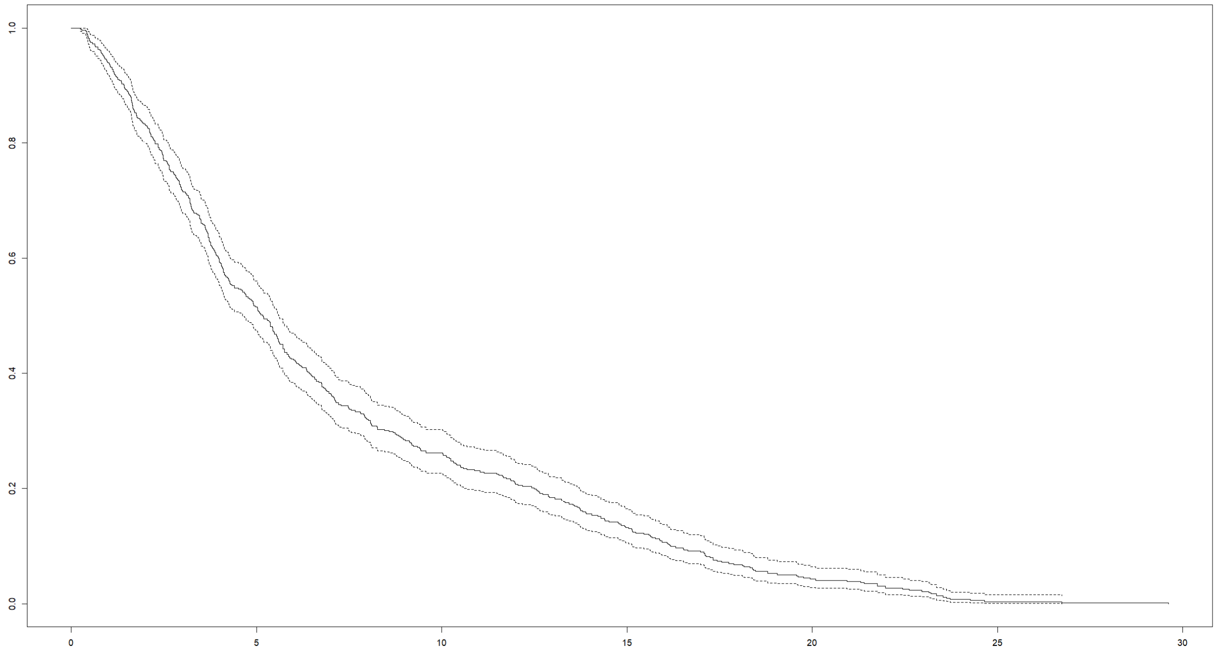

As suggested by Allison (2014) and Singer and Willett (2003), it is useful to estimate the empty model (without any covariate) and then plot its Martingale residuals vs. the continuous covariates of the full model (this is not useful for categorical covariates).

For age1C:

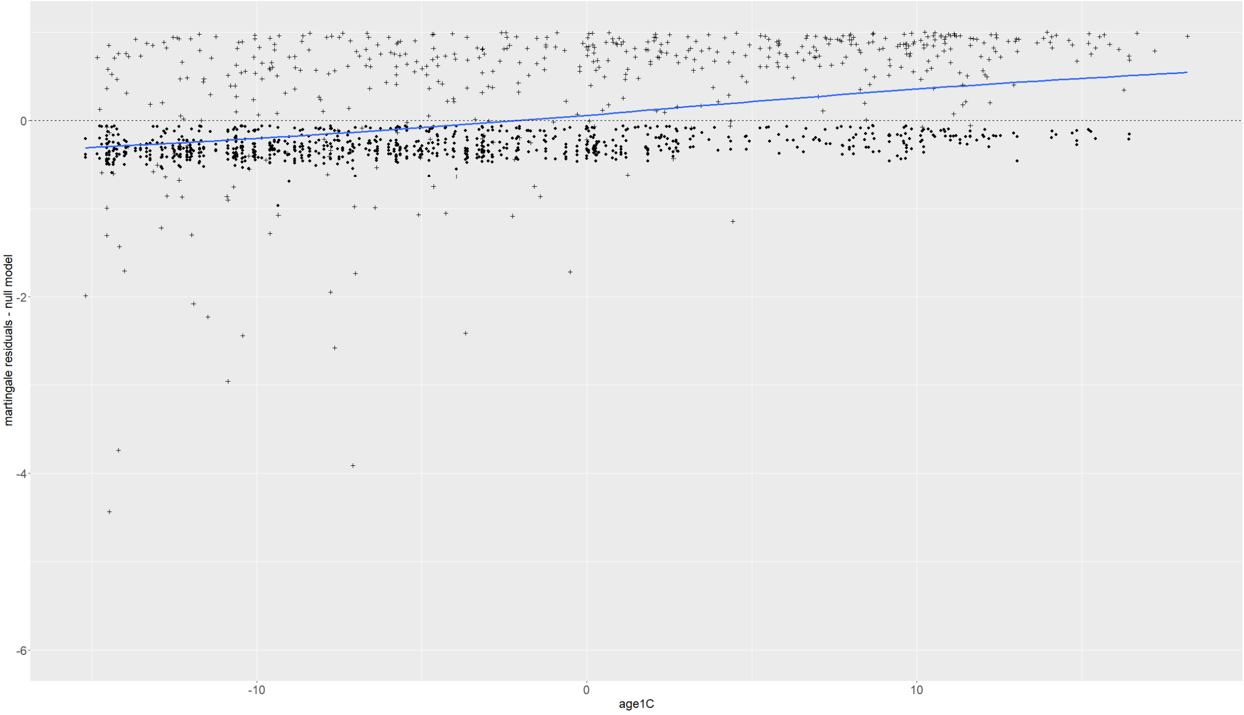

For initial age the relation appears linear, which means we can include this covariate as is in the full model.

For sesC:

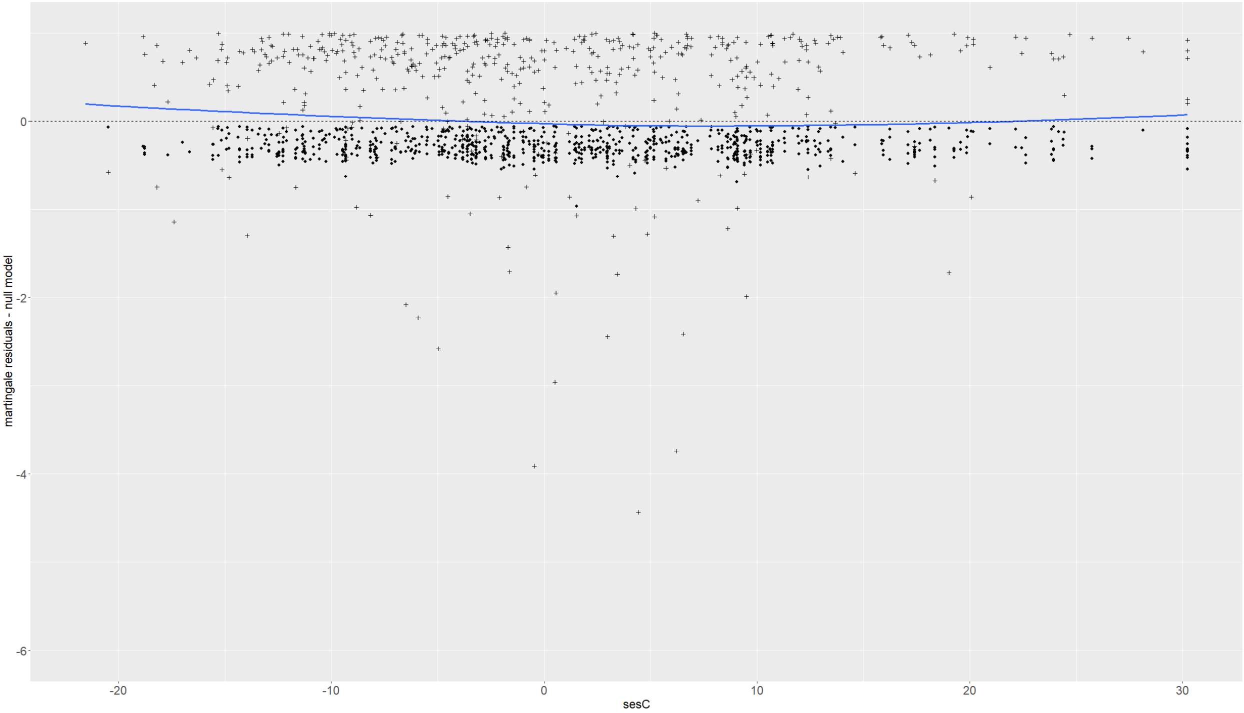

For socio-biographical status, the relation appears null, which means its presence in the full model will not jeopardize the quality of the model.

Estimates from the full model (cf. Supplementary Table 3):

```
> summary(surv2.fit)
Call:
coxph(formula = Surv(tstart, tstop, zmdead) ~ age1C + women +
    sesC + dementia, data = ssBASE)

  n= 1342, number of events= 516

              coef exp(coef)  se(coef)      z Pr(>|z|)
age1C     0.093904  1.098454  0.006555 14.326  < 2e-16 ***
women    -0.328128  0.720271  0.090837 -3.612 0.000304 ***
sesC     -0.002505  0.997498  0.004888 -0.512 0.608319
dementia  0.339231  1.403868  0.099269  3.417 0.000632 ***
---
Signif. codes:  0 ‘***’ 0.001 ‘**’ 0.01 ‘*’ 0.05 ‘.’ 0.1 ‘ ’ 1

         exp(coef) exp(-coef) lower .95 upper .95
age1C       1.0985     0.9104    1.0844    1.1127
women       0.7203     1.3884    0.6028    0.8606
sesC        0.9975     1.0025    0.9880    1.0071
dementia    1.4039     0.7123    1.1557    1.7054

Concordance= 0.727  (se = 0.012 )
Likelihood ratio test= 276.6  on 4 df,   p=<2e-16
Wald test            = 269.2  on 4 df,   p=<2e-16
Score (logrank) test = 291.4  on 4 df,   p=<2e-16
```

The proportionality of hazards assumption (PHA) appears met according to the Schoenfeld residuals (SR):

```
> zSchoen
          chisq df     p
age1C    3.0787  1 0.079
women    0.0335  1 0.855
sesC     0.0411  1 0.839
dementia 1.6733  1 0.196
GLOBAL   4.0693  4 0.397
```

The results are virtually the same when the rank transformation is applied to obtain the SR:

```
> zSchoen2
           chisq df    p
age1C    2.67809  1 0.10
women    0.00176  1 0.97
sesC     0.07879  1 0.78
dementia 2.08133  1 0.15
GLOBAL   4.16449  4 0.38
```

Residual analysis of full model

First, Schoenfeld residuals (SR).

SR for initial age (age1C)

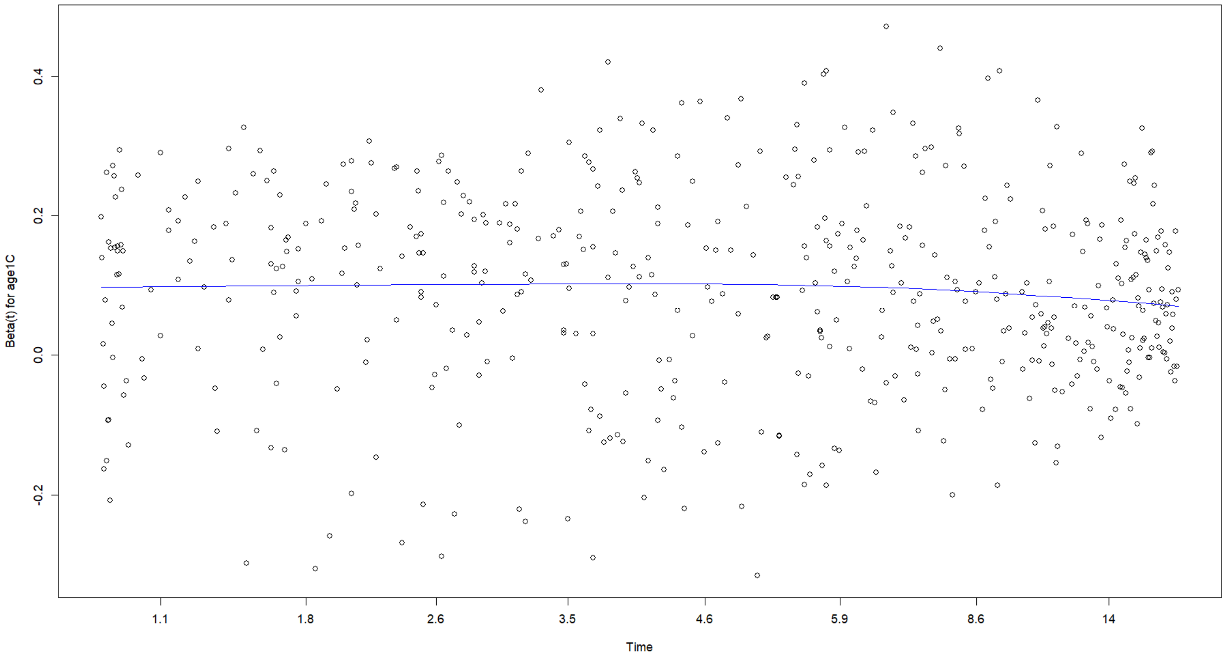

No trend appears, which tells us that the PHA is met for age1C in the full model.

SR for sex (women)

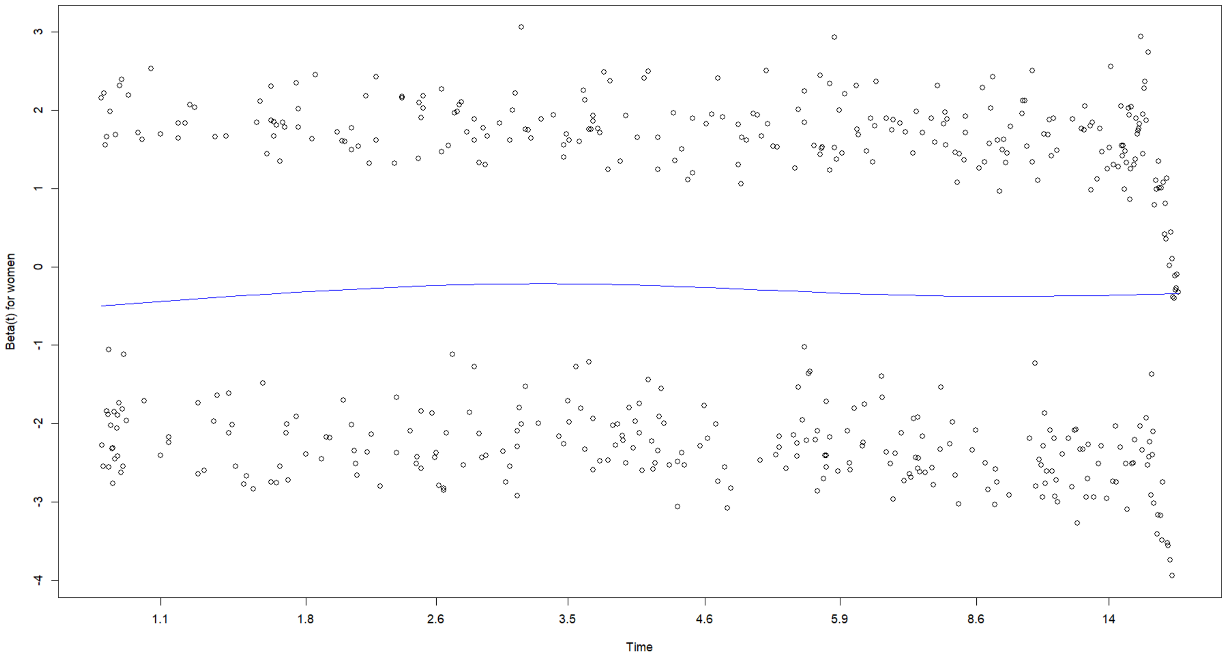

No trend appears, which tells us that the PHA is met for sex in the full model.

SR for socio-economic status, centered (sesC)

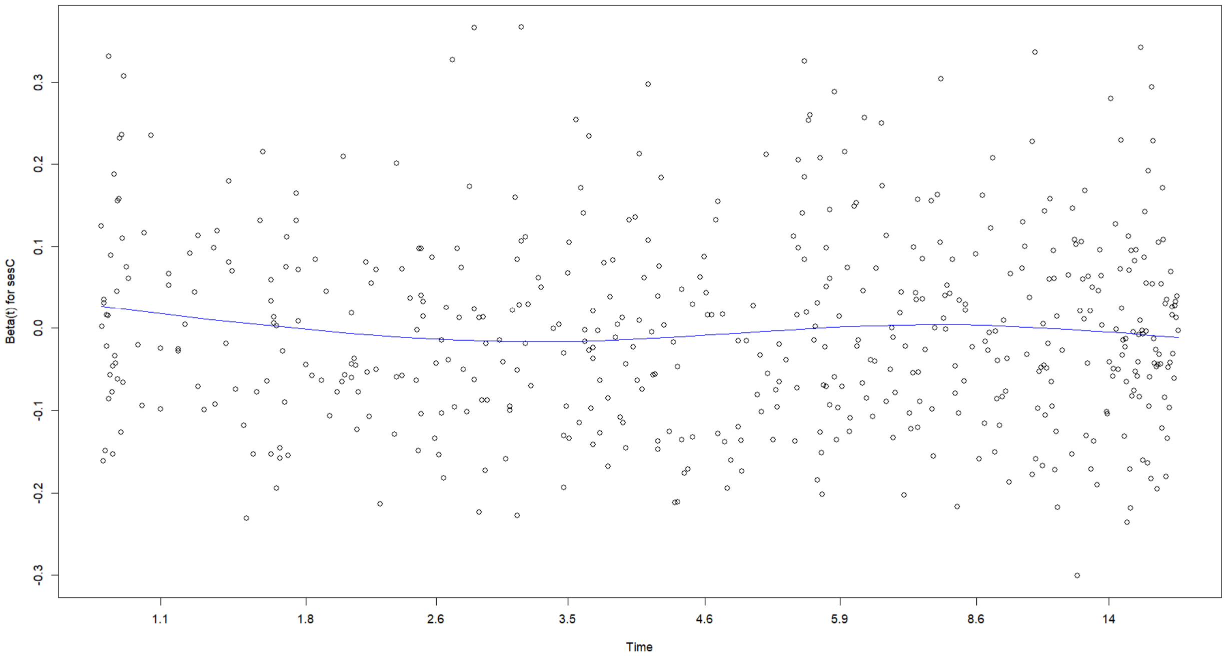

No trend appears, which tells us that the PHA is met for sesC in the full model.

SR for probable dementia diagnosis (dementia)

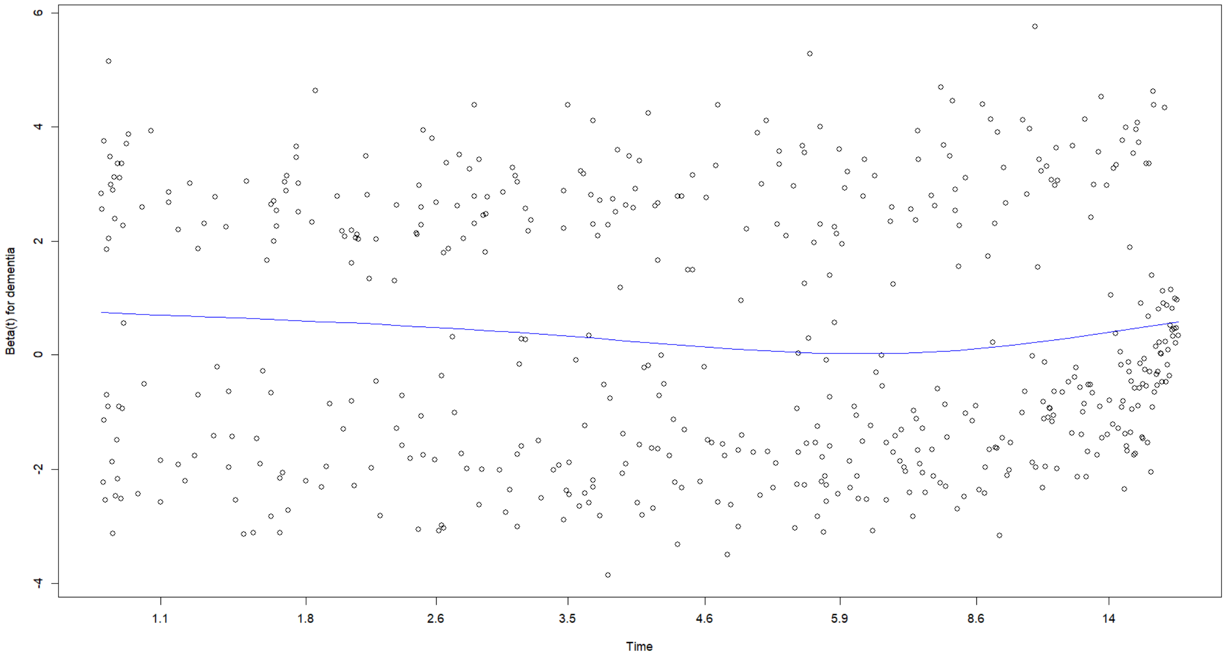

No trend appears, which tells us that the PHA is met for dementia in the full model.

Schoenfeld residuals vs. raking in time of death for age1c

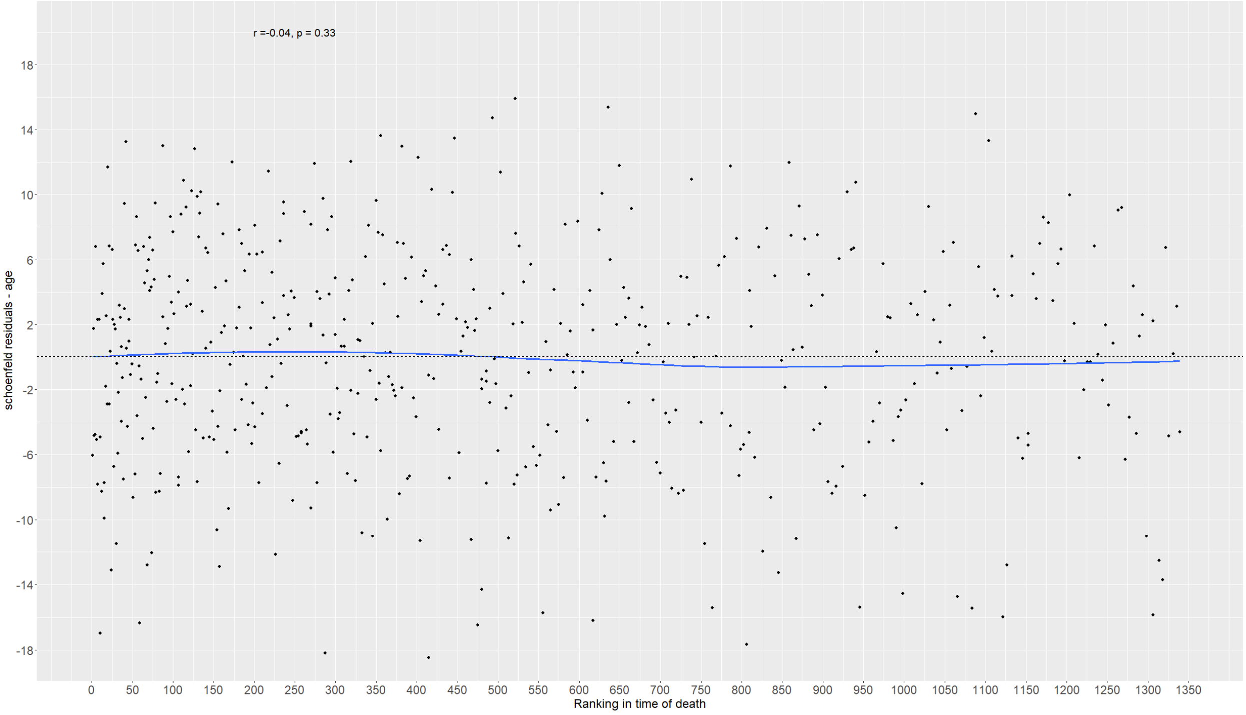

No correlation, no pattern confirms PHA is met.

Schoenfeld residuals vs. raking in time of death for women

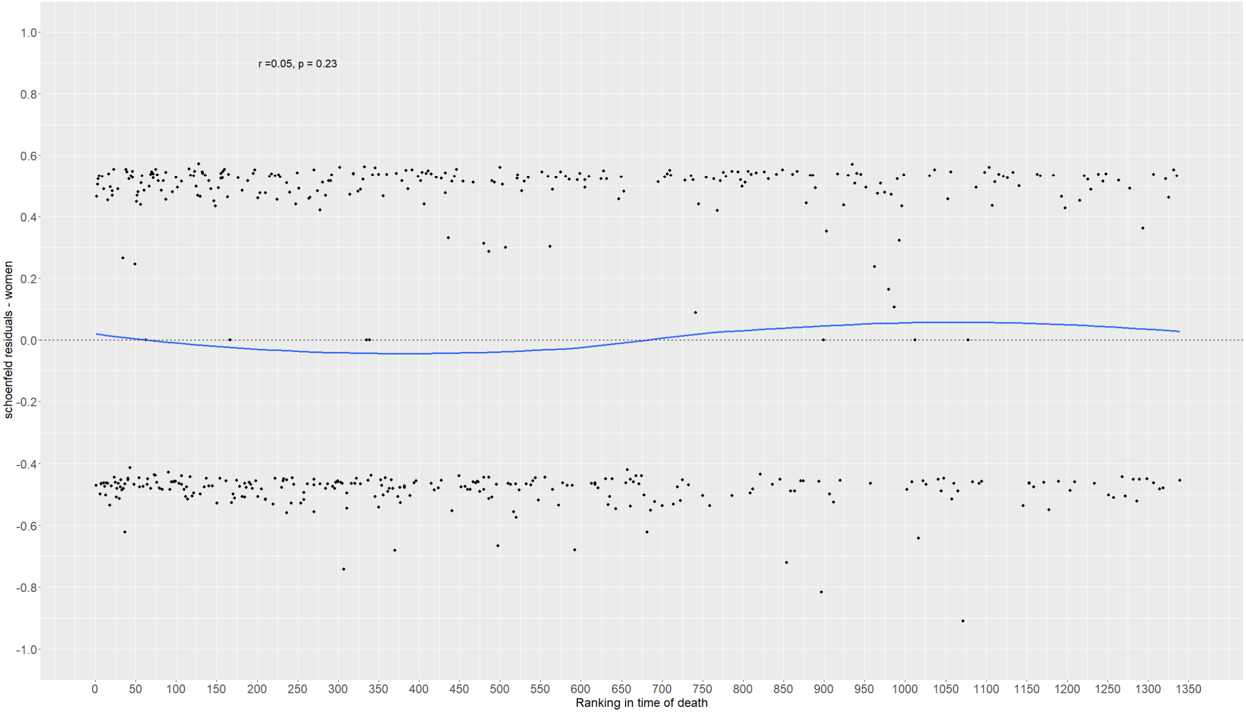

No correlation, no pattern confirms PHA is met.

Schoenfeld residuals vs. raking in time of death for sesC

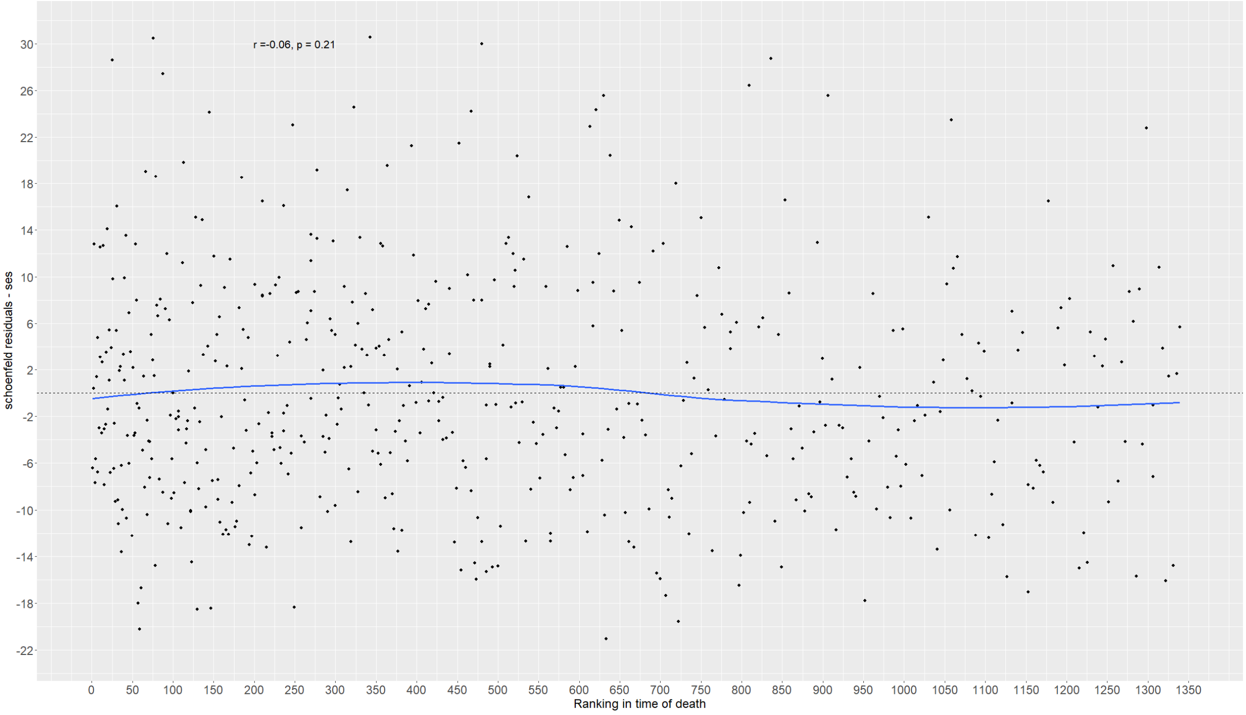

No correlation, no pattern confirms PHA is met.

Schoenfeld residuals vs. raking in time of death for dementia

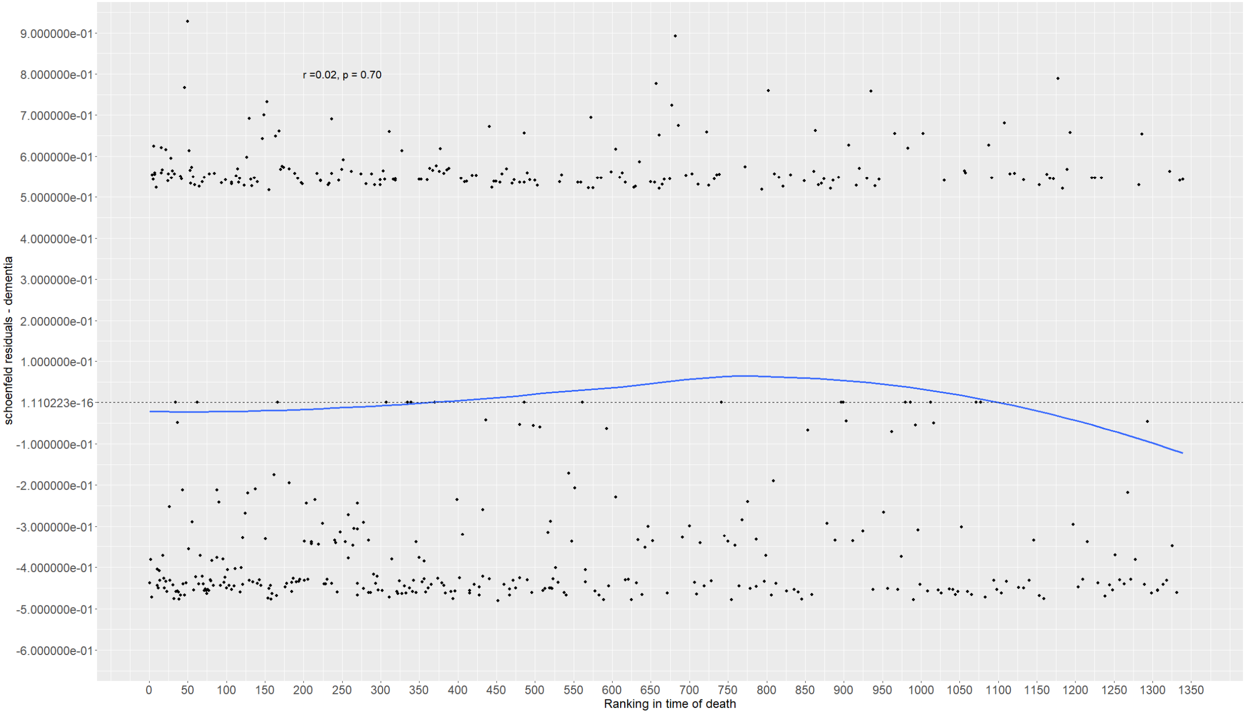

No correlation, no pattern confirms PHA is met.

Second, Martingale residuals.

Martingale residuals vs. age1c

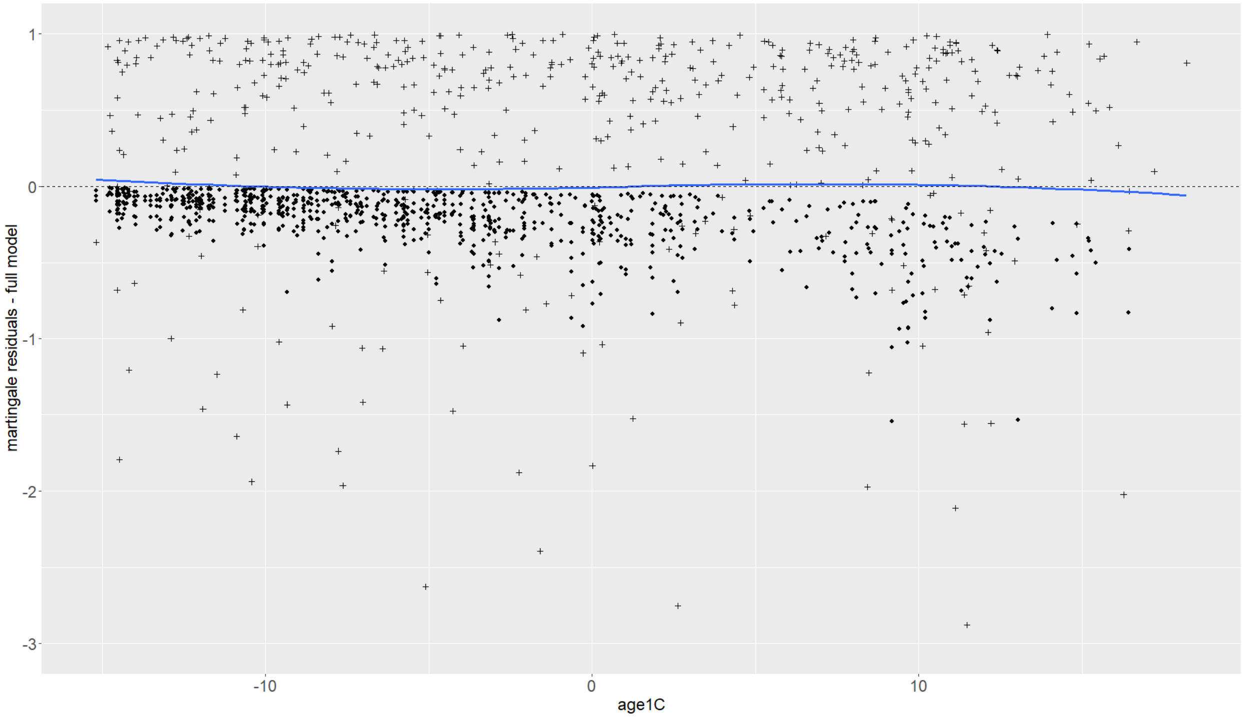

The positive linear trend visible in plot from empty model disappeared, which confirms the appropriateness of including linear age1c in the full model.

Martingale residuals vs. sesC

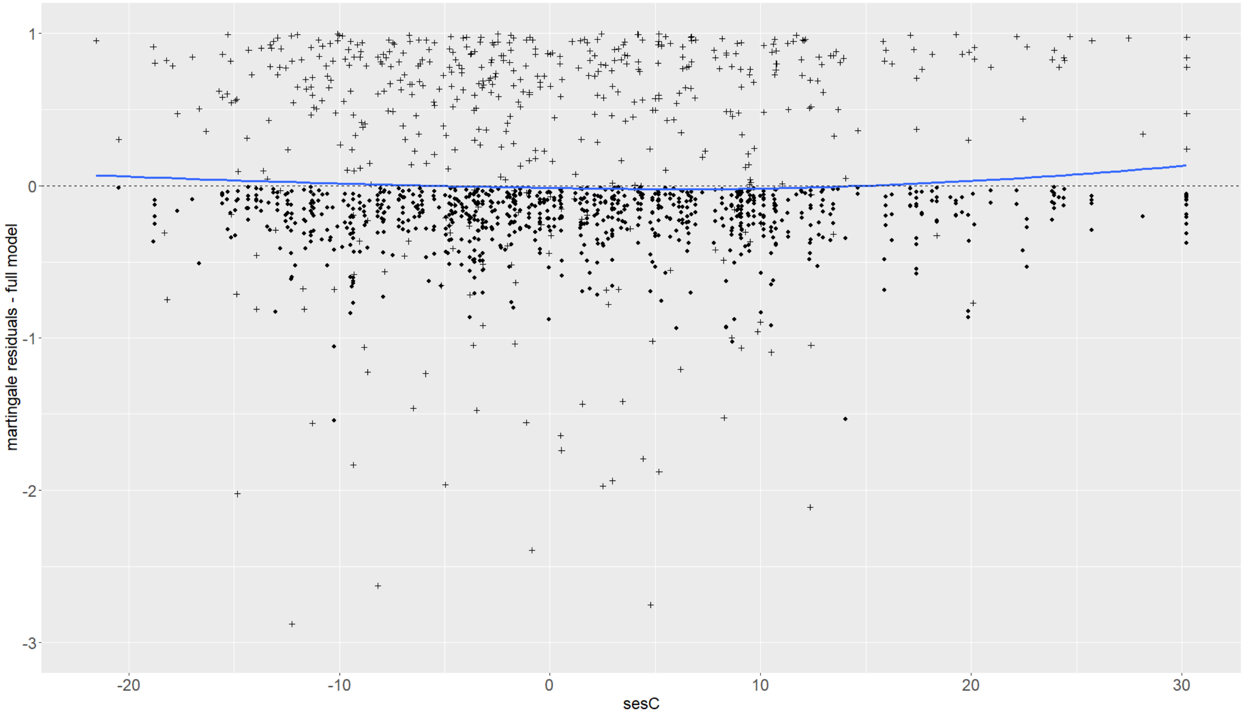

No trend appears, which confirms the appropriateness of including sesC in the full model.

Again, plots of Martingale residuals vs. categorical covariates are not useful.

Third, deviance residuals.

Risk scores vs. deviance residuals

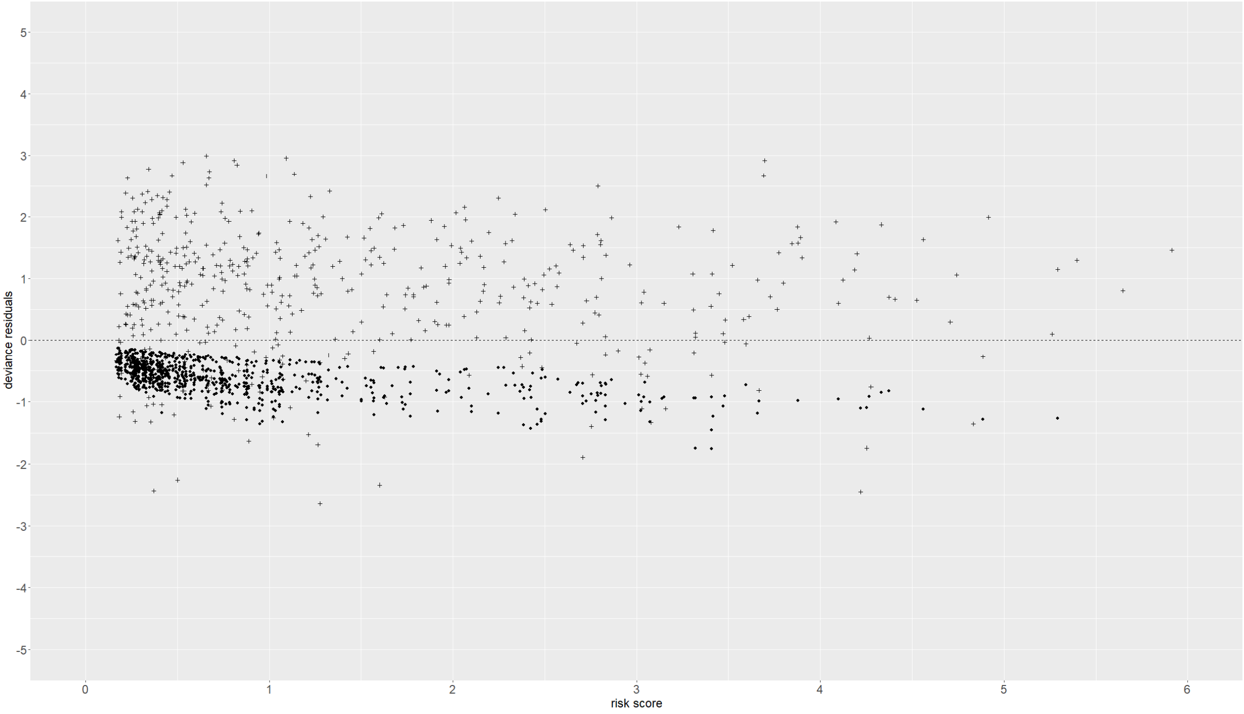


```
> cor(ssBASE$residDev, ssBASE$riskScore)
[1] 0.08128136
```

Deviance residuals appear unrelated to risk scores.

Fourth, score residuals.

Score residuals vs. age1c

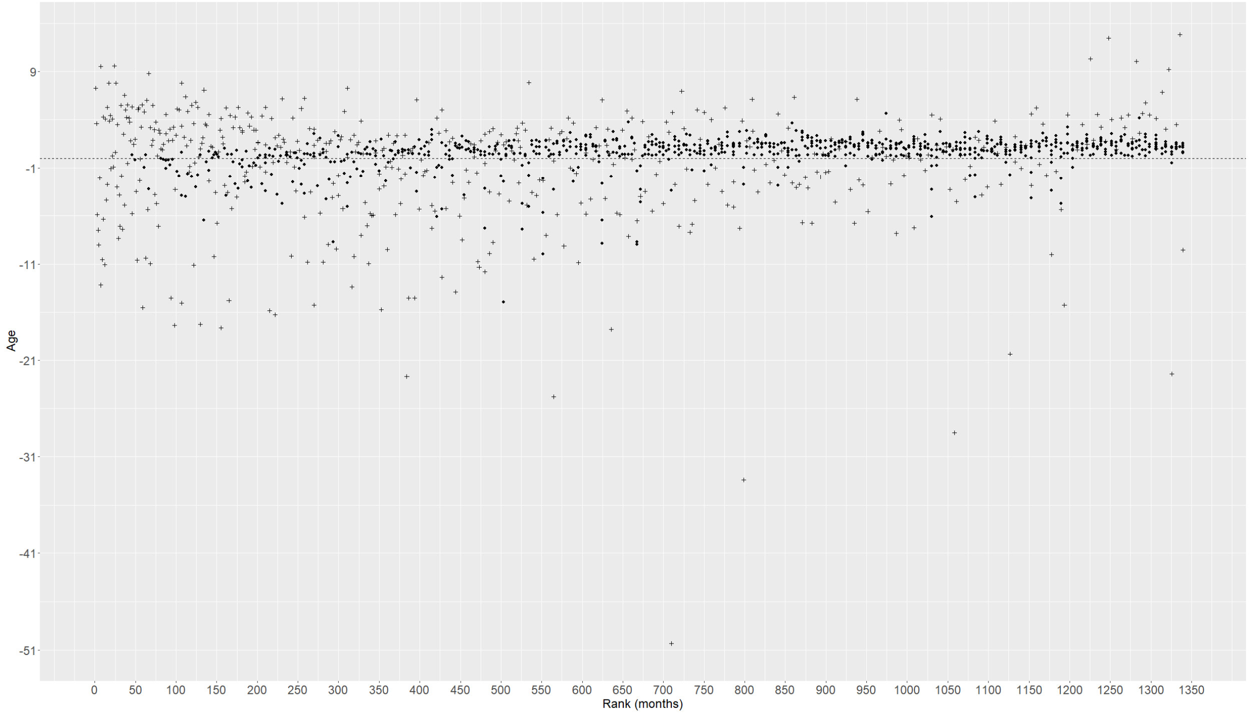

Very few extreme scores and all dispersed, so no reason to believe that a few, extreme scores are driving the estimation procedure.

Score residuals vs. women

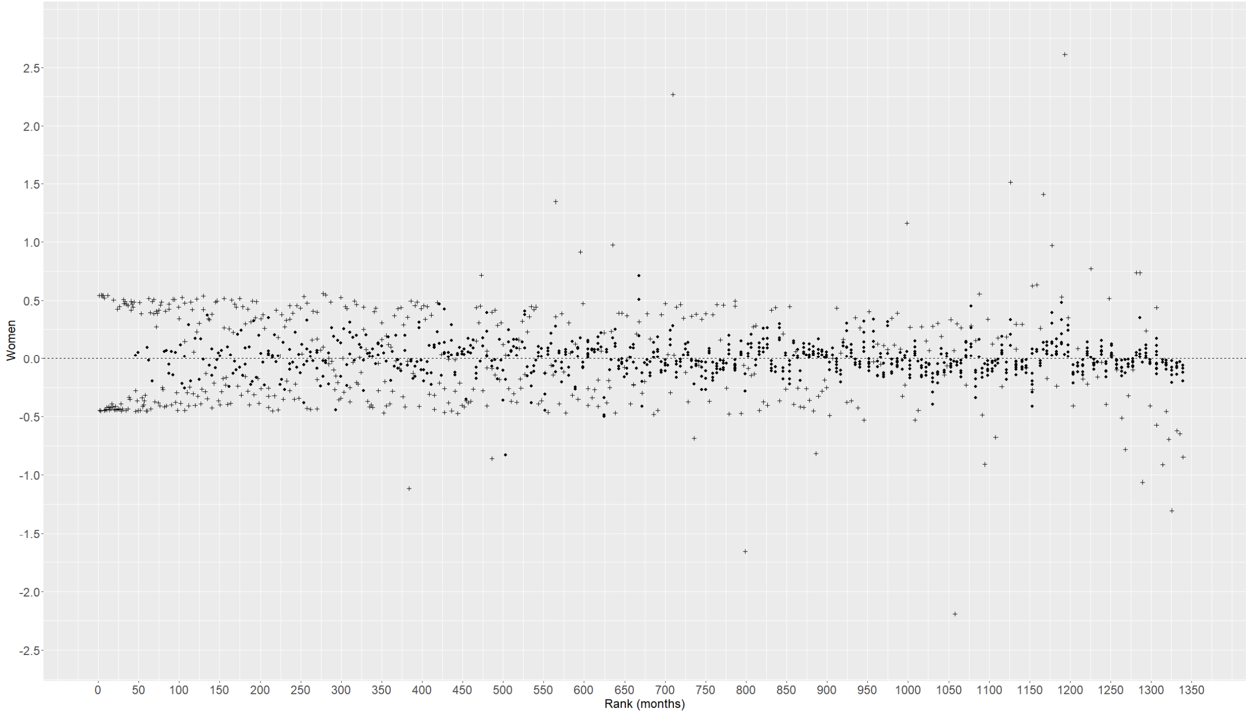

Very few extreme scores and all dispersed, so no reason to believe that a few, extreme scores are driving the estimation procedure.

Score residuals vs. sesC

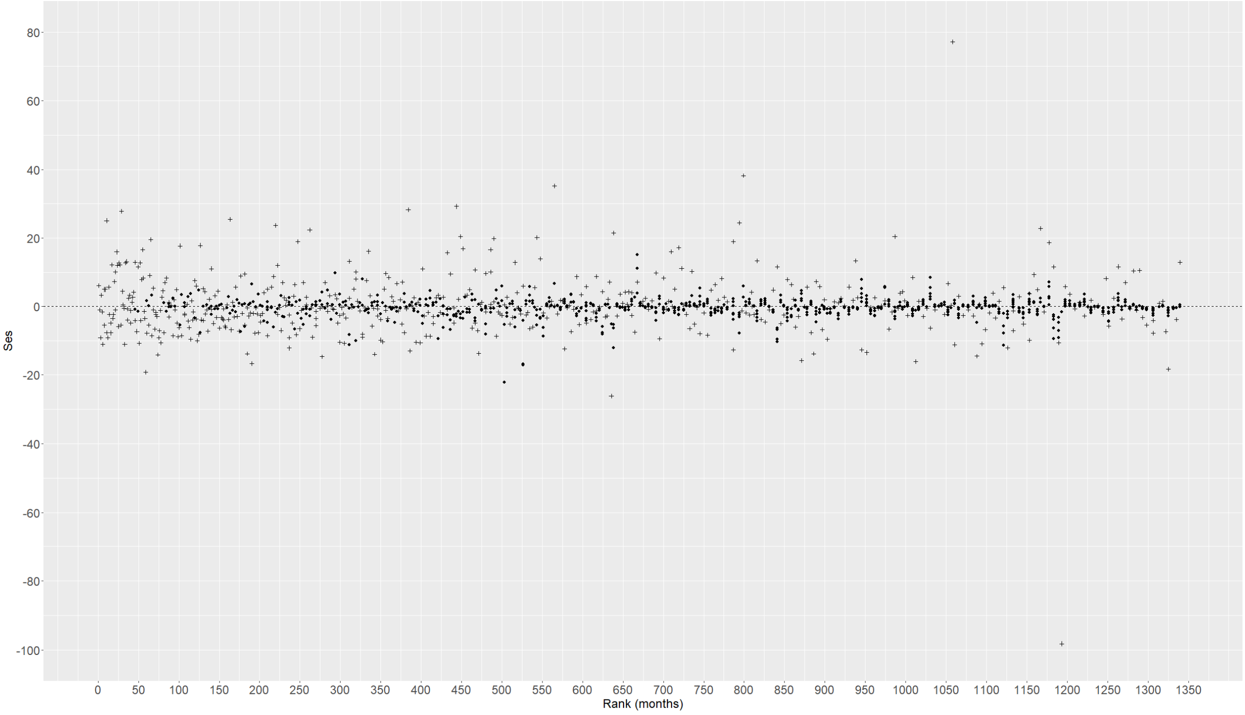

Very few extreme scores and all dispersed, so no reason to believe that a few, extreme scores are driving the estimation procedure.

Score residuals vs. dementia

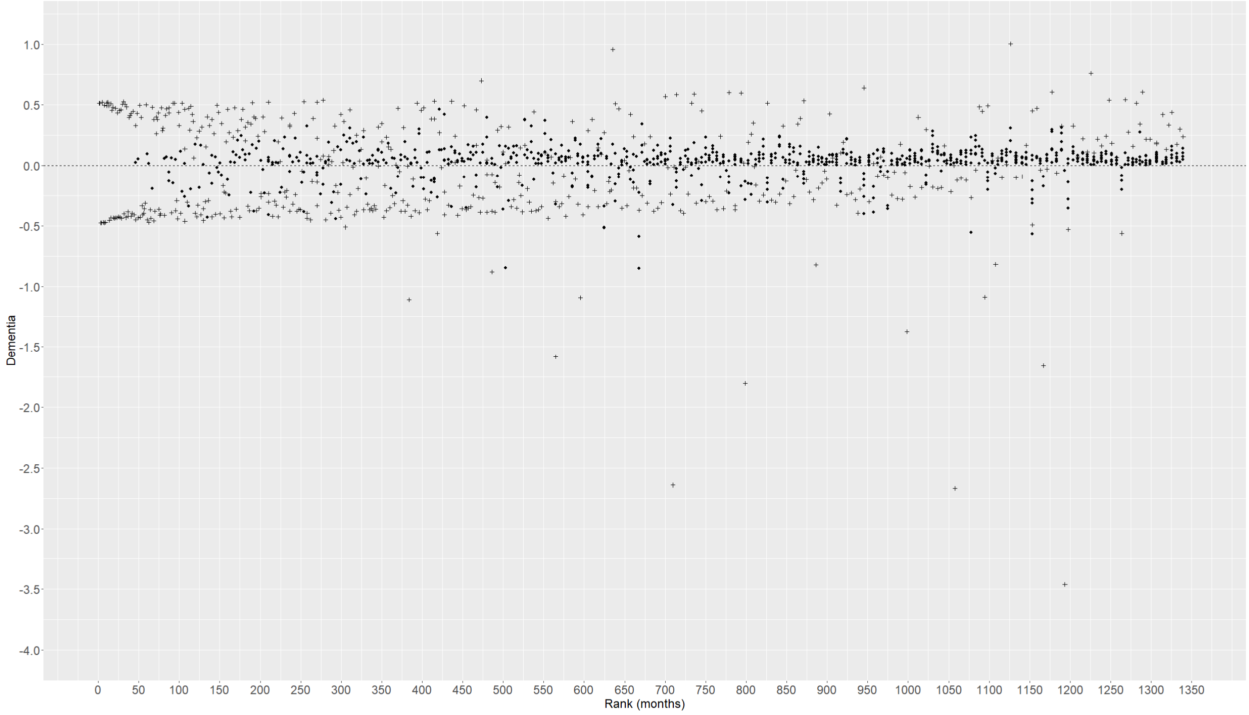

Very few extreme scores and all dispersed, so no reason to believe that a few, extreme scores are driving the estimation procedure.

Further analyses

Adding possible interactions between age1c, sesC, women, and dementia does not at all ameliorate the model's fit:

```
> anova(surv2.fit, surv2b.fit)
Analysis of Deviance Table
 Cox model: response is  Surv(tstart, tstop, zmdead)
 Model 1: ~ age1C + women + sesC + dementia
 Model 2: ~ age1C + women + sesC + dementia + agewomen + ageses + agedem + womenses
+ womendem + sesdem
   loglik  Chisq Df Pr(>|Chi|)
1 -2572.7
2 -2571.0 3.3923  6     0.7582
```

Conclusions

The full model with initial age, sex, SBS, and dementia appears appropriate to describe the survival process in this sample. We can thus employ it in the final JMLSM.

Supplementary Appendix 3

Description of results from the multivariate longitudinal submodel of the JMLSM (cf. Supplementary Table 3 and `2_JMLSM.R` *on* https://osf.io/u57gr/files/osfstorage*).*

The fixed effect estimates of the multivariate longitudinal submodel (Supplementary Table 3) show reliable decrease in time on all outcomes, with sharper estimates for the speed indicators and shallower ones for the knowledge tests. Residualized quadratic effects of time were not consistently different from zero, but when present, they indicated an acceleration of decline (i.e., they were negative). Retest effects emerged only on some tests, and on some occasions (as was the case in the univariate models; cf. Supplementary Table 1). Random effects estimates showed reliable variance in time on all indicators and non-zero variance in residualized squared time on all speed and fluency indicators. The intercorrelation matrix of the random effects across all tests is shown in the Supplementary Table 4 and correlations are predominantly positive and of moderate magnitude. The residual variance estimates were weakest for the speed and highest for the memory tests.

Supplementary Appendix 4

Diagnostics of the Markov chain Monte Carlo estimation used in the Joint Multivariate Longitudinal Survival Model (cf. `2_JMLSM.R` *on* https://osf.io/u57gr/files/osfstorage*)*

The JMbayes2 package integrates functions from the Coda package (Plummer et al., 2006), thereby allowing for detailed diagnostics about model fit and parameter estimates obtained by Markov chain Monte Carlo (MCMC) estimation. All the diagnostics about the Joint Multivariate Longitudinal Survival Model (JMLSM) indicate that the model appears to have converged very well. More precisely:

- *Potential scale reduction factor* (PSRF). This index expresses the factor by which variability in estimates across the multiple estimation chains of the MCMC reduces with additional iterations (Gelman & Rubin, 1992). The PSRF for all parameters was well below the commonly used 1.1 threshold. Their median value was 1.01 and the upper median confidence interval value was 1.04. The multivariate PSRF, which summarizes the estimation variability across the multiple chains for all parameters together, was estimated at 1.01.
- *Trace plots*. These plots show, for a given parameter, the MCMC iteration number on the abscissa and the estimated value on the ordinate. The trace plots of all survival parameter estimates (including α_1 and α_2) are presented in the Supplementary Figure 1. All trace plots assume the desired "hairy caterpillar" shape, characteristic of low serial correlations across estimated values. Also, the trace plots oscillate around a stable estimate (i.e., are not wiggly), indicating that the chains converged to the target posterior distribution. Finally, the trace plots obtained from the three estimation chains overlap consistently.
- *Posterior density plots*. These plots show, for a given parameter, the cumulated estimated values across all the iterations, for each estimation chain. The posterior density plots of all

survival parameter estimates (including α_1 and α_2) are presented in the Supplementary Figure 2 and all follow peaked symmetrical distributions around the most credible value for each parameter. Moreover, the posterior density plots obtained from the three estimation chains overlap greatly.

- *Cumulative quantile plots*. These plots show, for a given parameter, the evolution of the estimated value on the ordinate across all iterations depicted in the abscissa. The cumulative quantile plots from each estimation chain for all survival parameters (including α_1 and α_2) are presented in the Supplementary Figure 3 and all show that after at most about 4,000 quantiles, and very often much sooner, all parameter estimates were highly stable around a single value. Thus, we interpret results based on 16,000 quantiles with high confidence.

Supplementary Appendix 5

Sensitivity analyses of the JMLSM omitting predictive cognitive tasks (cf. `3_sensitivity_JMLSM.R` *on* https://osf.io/u57gr/files/osfstorage*)*

To ascertain the robustness of the results from the full JMLSM, we tested four additional specifications by omitting, in turns, the following predictors from the full model:

- Model 1: $\boldsymbol{m}_{iCA}(t)$, the current value of CA
- Model 2: $\boldsymbol{m}_{iWB}(t)$, the current value of WB
- Model 3: $\boldsymbol{m}_{iCA}(t)$ and $\boldsymbol{m}'_{iCA}(t)$, the current value and the current rate of change of CA
- Model 4: $\boldsymbol{m}_{iWB}(t)$ and $\boldsymbol{m}'_{iWB}(t)$, the current value and the current rate of change of WB

Parameter Estimates and 95% Credible Intervals from the Survival Submodel of the 4 additional JMLSM (cols. 2–5) and of the Full Model (col. 6; N = 516):

Predictor	Model 1	Model 2	Model 3	Model 4	Full JMLSM
Initial Age	0.07 [0.05;0.10]	0.07 [0.05;0.10]	0.08 [0.05;0.10]	0.07 [0.05;0.10]	0.08 [0.05;0.10]
Sex	–0.32 [–0.53;–0.10]	–0.37 [–0.59;–0.16]	–0.31 [–0.52;–0.09]	–0.37 [–0.59;–0.16]	–0.36 [–0.58;–0.15]
Socio-biographical status	*–0.008 [–0.01;0.02]*	*0.001 [–0.01;0.02]*	*0.001 [–0.01;0.02]*	*0.001 [–0.01;0.02]*	*0.01 [–0.01;0.02]*
Dementia	*0.09 [–0.15;0.33]*	*0.06 [–0.18;0.30]*	*0.14 [–0.10;0.38]*	*0.06 [–0.18;0.30]*	*0.07 [–0.17;0.31]*
α_{1CA} ($m_{iCA}(t)$)	–	–0.07 [–0.09;–0.04]	–	–0.07 [–0.09;–0.04]	–0.05 [–0.08;–0.03]
α_{2CA} ($m'_{iCA}(t)$)	*0.01 [–0.01;0.01]*	*0.01 [–0.01;0.01]*	–	*0.01 [–0.01;0.01]*	*0.01 [–0.01;0.01]*
α_{1WB} ($m_{iWB}(t)$)	–0.05 [–0.07;–0.02]	–	–0.05 [–0.08;–0.02]	–	–0.03 [–0.05;–0.01]
α_{2WB} ($m'_{iWB}(t)$)	*0.01 [–0.01;0.01]*	*0.01 [–0.01;0.01]*	*0.01 [–0.01;0.01]*	–	*0.01 [–0.01;0.01]*

Notes. Sex is coded men=0, women=1; Dementia = occasion-specific assessment of suspected dementia (0=no, 1=yes); Italicized numbers correspond to effects with a 95% credible interval that includes 0 (null effects); All 516 participants died (no right-censorship).

The outcomes of these 4 models (cols. 2–5) did not differ substantially from those of the full JMLSM reported in the text in Table 2 (col. 6). Initial age and sex were consistently predictive of survival, whereas socio-biographical status and suspected dementia were not predictive. Moreover, the parameter estimates of current rates of change (α_2 of either CA or WB) remained null despite the omission of the respective parameters for current values (α_1). Finally, when one fluency task was excluded from the model, the parameter estimates concerning the other fluency task did not change considerably compared to the full JMLSM.

Supplementary Appendix 6

Parameter estimates and 95% credible intervals from the survival submodels of the 10 Joint Univariate Longitudinal Survival Models of each cognitive task and of the composite general intelligence score G (N = 516; cf. `4_10XJLSM.R` *on https://osf.io/u57gr/files/osfstorage)*

	DS	DL	IP	PA	MT	CA	WB	VO	SW	G
α_1	–0.029	–0.033	–0.028	–0.025	–0.025	–0.053	–0.046	–0.016	*–0.011*	–0.048
	[–0.045;–0.015]	[–0.047;–0.021]	[–0.046;–0.013]	[–0.041;–0.009]	[–0.050;–0.001]	[–0.068;–0.038]	[–0.063;–0.030]	[–0.031;–0.001]	*[–0.026;0.004]*	[–0.064;–0.033]
α_2	*0.0003*	*0.001*	*0.001*	*0.001*	*–0.001*	*0.0003*	*0.001*	*–0.001*	*0.001*	*0.001*
	[–0.002;0.003]	*[–0.001;0.004]*	*[–0.001;0.003]*	*[–0.007;0.007]*	*[–0.012;0.006]*	*[–0.002;0.003]*	*[–0.001;0.004]*	*[–0.003;0.003]*	*[–0.002;0.002]*	*[–0.002;0.005]*

Note. α_1 = survival effect of current value of a given cognitive outcome; α_2 = survival effect of current rate of change value of a given cognitive outcome; DS = digit symbol; DL = digit letter; IP = identical pictures; PA = paired associates; MT = memory for text; CA = categories; WB = word beginning; VO = vocabulary; SW = spot–a–word; G = general intelligence score; Italicized numbers correspond to effects with a 95% credible interval that includes 0 (null effects); All 516 participants died.

These estimates stem from 10 joint longitudinal and survival models that consider separately each cognitive task or the composite G score as predictor of survival (controlling for initial age, sex, socio-biographical status, and suspected dementia). As such, the estimates α_1 and α_2 of each cognitive task do not take into account (i.e., are not residualized for) the effects of the other cognitive tasks. For all cognitive variables excepted SW (which had no effect), a higher current value at time *t* lowers the risk of death at that time *t*. And, for all cognitive variables, their current rate of change did not impact survival. In absolute terms, CA had the strongest effect (α_1 = –0.053), followed by G (α_1 = –0.048) and WB (α_1 = –0.046).

Supplementary Appendix 7

Parameter estimates and 95% credible intervals from the survival submodels of the 9 Joint Bivariate Longitudinal Survival Models of each cognitive Task with the composite for general intelligence score G (N = 516; cf. `5_9xJBLSM.R` *on https://osf.io/u57gr/files/osfstorage)*

		DS	DL	IP	PA	MT	CA	WB	VO	SW
	α_1	*0.004 [–0.017;0.029]*	*–0.003 [–0.025;0.019]*	*0.013 [–0.015;0.042]*	*0.016 [–0.013;0.046]*	0.053 [0.002;0.119]	–0.042 [–0.071;–0.014]	*–0.027 [–0.055;0.002]*	0.066 [0.033;0.103]	0.045 [0.017;0.076]
	α_2	*0.0002 [–0.002;0.003]*	*0.001 [–0.002;0.004]*	*0.0005 [–0.001;0.003]*	*0.0002 [–0.008;0.009]*	*–0.001 [–0.012;0.005]*	*–0.0001 [–0.002;0.002]*	*0.001 [–0.002;0.004]*	*–0.0003 [–0.003;0.003]*	*0.0002 [–0.004;0.004]*
G	α_1	–0.052 [–0.078;–0.030]	–0.048 [–0.074;–0.023]	–0.059 [–0.089;–0.031]	–0.062 [–0.093;–0.034]	–0.084 [–0.134;–0.045]	*–0.014 [–0.044;0.016]*	*–0.024 [–0.053;0.004]*	–0.105 [–0.142;–0.071]	–0.081 [–0.112;–0.053]
G	α_2	*0.001 [–0.002;0.005]*	*0.0004 [–0.003;0.004]*	*0.001 [–0.004;0.004]*	*0.001 [–0.004;0.007]*	*0.001 [–0.003;0.006]*	*0.001 [–0.002;0.005]*	*0.0002 [–0.003;0.004]*	*0.001 [–0.002;0.005]*	*0.001 [–0.002;0.005]*

Note. α_1 = survival effect of current value of a given cognitive outcome; α_2 = survival effect of current rate of change value of a given cognitive outcome; Estimates in the first two rows are for cognitive tasks (indicated in columns); estimates in third and fourth row are for the composite of general intelligence score G (indicated in the first column); DS = digit symbol; DL = digit letter; IP = identical pictures; PA = paired associates; MT = memory for text; CA = categories; WB = word beginning; VO = vocabulary; SW = spot–a–word; G = general intelligence score; Italicized numbers correspond to effects with a 95% credible interval that includes 0 (null effects); All 516 participants died.

These estimates stem from 9 joint bivariate longitudinal and survival models that consider separately each cognitive task with the composite G score as predictors of survival (controlling for initial age, sex, socio-biographical status, and suspected dementia). As such, the estimates α_1 and α_2 of each cognitive task do not take into account the effects of the other cognitive tasks, but are residualized for the effects of G (likewise, the effects for G are controlled only for the effects of one cognitive task at a time). A higher current value of G at time *t* lowers the risk of death at that time *t* when G is analyzed with any cognitive task excepted with CA or WB. The bivariate model with G and CA obtained that only a higher current value of CA (α_1 = –0.042), but not of G, predicted survival. The current value of three other cognitive tasks affected the risk of death, but

in a counterintuitive direction ($\alpha_1 > 0$): MT obtained $\alpha_1 = 0.053$, VO obtained $\alpha_1 = 0.066$, and SW obtained $\alpha_1 = 0.045$. Consequently, in these three bivariate models the estimate of current value for G obtained a stronger magnitude than usual, but in the expected negative direction (α_1 = –0.084, –0.105, and –0.081), to counteract the unexpected residualized positive effect of each of the three cognitive tasks. Across all bivariate models, the current rate of change values never appeared predictive of survival.

Supplementary Appendix 8

Two-stage analyses of cognitive change and survival (cf. `6_2-stage_cognitive_change_and_survival.R` *on https://osf.io/u57gr/files/osfstorage)*

We compared the joint estimation procedure with a more classical two-stage procedure, in which: (a) during the first stage cognitive change is studied and individual characteristics about cognitive change are estimated and outputted; and (b) during the second stage, the estimated individual characteristics are tested as covariates in the survival model. In the first stage we modeled each cognitive variable as follows:

$$Y_{iqt} = (\beta_{0q} + b_{0iq}) + (\beta_{1q} + b_{1iq}) \times time_{it} + (\beta_{2q} + b_{2iq}) \times time^2_{it} + \beta_{3q} \times I.Age_i + \beta_{4q} \times (I.Age_i \times time_{it}) + \beta_{qw} \times R_{qw} + e_{iqt},$$

where the indices i, q, and t indicate, respectively, individuals, the cognitive variable analyzed, and time; β's and b's represent fixed and random effects, respectively; $time_{it}$ indicates time in study since initial assessment 1 (in 1990-1993); $time^2_{it}$ indicates the component of time squared residualized for $time_{it}$; $I.Age_i$ indicates initial age (at initial assessment 1); β_{qw} indicates the effect of the R_{qw} retest effect of variable q on wave w; and e_{iqt} is the final error of prediction. For each individual i, we then estimated their random effects $b_{.iq}$ (with BLUPs, best linear unbiased predictors, via the `ranef` function in the `nlme` package) and combined these with their individual characteristics (initial age and time of assessment) and with the group characteristics (fixed effects $\beta_{.q}$) to obtain their predicted score $\hat{Y}_{iqt}$, for variable q at time t (where $Y_{iqt} = \hat{Y}_{iqt} + e_{iqt}$), which are equivalent to the estimated scores obtained with the `predict` function in the `nlme` package. The $\hat{Y}_{iqt}$ scores represent the

current values m_{iqt} (the true, unobserved scores of the qth outcome at time t for the ith individual). We then derived with respect to time t the mixed-effects model specified for $\hat{Y}_{iqt}$ to obtain m'_{iqt} as follows:

$$m'_{iqt} = (d\, m_{iq}(t)) / dt = (\beta_{1q} + b_{1iq}) + 2 \times (\beta_{2q} + b_{2iq}) \times time_{it} + \beta_{4q} \times (I.Age_i),$$

which represents the current rate of change (for individual i, at time t, on cognitive variable q). Thus, for each cognitive variable q, each individual i had a vector of estimated scores of current values $\boldsymbol{m}_{iqt}$ and current rate of change values $\boldsymbol{m'}_{iqt}$ (for as many times of assessment). In the second stage, both estimated cognitive scores are added as time-varying predictors in the following extended Cox model:

$$h_{it} = h_{0t} \times \exp[(\gamma_1 \times I.Age_i) + (\gamma_2 \times Sex_i) + (\gamma_3 \times SBS_i) + (\gamma_4 \times Dementia_{it}) + (\alpha_{1q} \times m_{iqt}) + (\alpha_{2q} \times m'_{iqt})],$$

where $I.Age_i$ is again initial age (at initial assessment 1), Sex_i is coded 1 for women and 0 for men, SBS_i represents socio-biographical status, and $Dementia_{it}$ is the time-varying predictor coded 1 for suspected dementia and 0 for lack of suspicion of dementia at time t.

To avoid confounds due to the estimation procedure (Markov chain Monte Carlo in the joint model vs. maximum likelihood in the two-stage approach) we used MCMC for the survival model (with the `survregbayes` function of the `spBayesSurv` package in R, version 1.1.8; Zhou et al., 2020).

Parameter Estimates and 95% Credible Intervals of the Cognitive Tasks from the 2-Stage Multivariate Longitudinal Survival Model (N = 516)

	DS	DL	IP	PA	MT	CA	WB	VO	SW
α_1	*–0.004* *[–0.023;0.014]*	*–0.002* *[–0.021;0.013]*	*–0.008* *[–0.028;0.011]*	*–0.008* *[–0.025;0.012]*	*0.001* *[–0.024;0.026]*	–0.043 [–0.063;–0.023]	*–0.009* *[–0.024;0.008]*	0.022 [0.003;0.045]	*–0.008* *[–0.023;0.011]*
α_2	*–0.050* *[–0.194;0.082]*	*–0.076* *[–0.225;0.073]*	*0.050* *[–0.138;0.230]*	*–0.005* *[–0.688;0.686]*	*0.165* *[–0.289;0.559]*	*–0.110* *[–0.251;0.039]*	*0.058* *[–0.093;0.216]*	*0.448* *[–0.353;1.515]*	*0.335* *[–0.090;0.741]*

Note. α_1 = survival effect of current value of a given cognitive outcome; α_2 = survival effect of current rate of change value of a given cognitive outcome; Estimates in the first two rows are for cognitive tasks (indicated in columns); estimates in third and fourth row are for the composite of general intelligence score G (indicated in the first column); DS = digit symbol; DL = digit letter; IP = identical pictures; PA = paired associates; MT = memory for text; CA = categories; WB = word beginning; VO = vocabulary; SW = spot–a–word; G = general intelligence score; Italicized numbers correspond to effects with a 95% credible interval that includes 0 (null effects); All 516 participants died.

Credible intervals (CIs) were more precise for the joint compared to the 2-stage estimation (mean and median range of CIs were 0.039 and 0.051 for the joint estimation vs. 0.378 and 0.163 for the 2-stage estimation). Thus, as expected, the joint estimation was more precise (i.e., efficient) than the two-stage estimation. The 2-stage estimation confirmed the predictive validity of category death (α_1 = –0.043, [–0.063;–0.023]) and strangely obtained that high vocabulary values are predictive of early death (α_1 = 0.022, [0.003;0.045]). Substantively, interpreting this counterintuitive result is very challenging, if not illusory.

Finally, we tested each cognitive task and G in separate univariate Cox survival regression models (controlling for initial age, sex, socio-biographical status, and suspected dementia) with the two-stage estimation.

Parameter Estimates and 95% Credible Intervals of the Cognitive Tasks from the 10 Univariate Longitudinal Survival Models with 2-Stage Estimation (N = 516)

	DS	DL	IP	PA	MT	CA	WB	VO	SW	G
α_1	–0.026<br>[–0.038;–0.014]	–0.026<br>[–0.036;–0.014]	–0.025<br>[–0.036;–0.013]	*–0.018*<br>*[–0.040;0.004]*	*–0.018*<br>*[–0.040;0.004]*	–0.049<br>[–0.061;–0.033]	–0.035<br>[–0.050;–0.022]	–0.014<br>[–0.028;–0.001]	NC	–0.039<br>[–0.052;–0.024]

α_2	*–0.066 [–0.205;0.086]*	*–0.079 [–0.202;0.071]*	*0.057 [–0.014;0.147]*	*–0.105 [–0.893;0.593]*	*–0.012 [–0.517;0.465]*	*–0.094 [–0.211;0.024]*	*0.041 [–0.072;0.160]*	*0.322 [–0.218;0.892]*	*–0.085 [–0.247;0.092]*

Note. α_1 = survival effect of current value of a given cognitive outcome; α_2 = survival effect of current rate of change value of a given cognitive outcome; DS = digit symbol; DL = digit letter; IP = identical pictures; PA = paired associates; MT = memory for text; CA = categories; WB = word beginning; VO = vocabulary; SW = spot–a–word; G = general intelligence score; NC = no convergence attained; Italicized numbers correspond to effects with a 95% credible interval that includes 0 (null effects); All 516 participants died.

Credible intervals (CIs) were again more precise for the joint compared to the 2-stage estimation (mean and median range of CIs were 0.039 and 0.051 vs. 0.269 and 0.281). Compared to the univariate joint estimation (cf. Supplementary Appendix 6), the effects of the two episodic memory tasks is not detected, and estimation is again less precise (i.e., wider credible intervals).

Supplementary Appendix 9

Additional survival analyses concerning socio-biographical status (cf.

`7_additional_SBS_survival.R` *on https://osf.io/u57gr/files/osfstorage)*

We analyzed the interrelationships among the 4 constituent variables that were collapsed in the SBS composite score:

```
> cor(BASEsurv[,c(6:10)], use="pairwise.complete.obs", method="pearson")
             sesC    k1zoekl    s1kon18    s1kon32    s1kon28
sesC    1.0000000 0.6346221 0.7208005 0.7529342 0.7845398
k1zoekl 0.6346221 1.0000000 0.2789321 0.2752377 0.2936978
s1kon18 0.7208005 0.2789321 1.0000000 0.4559233 0.5986493
s1kon32 0.7529342 0.2752377 0.4559233 1.0000000 0.5009417
s1kon28 0.7845398 0.2936978 0.5986493 0.5009417 1.0000000
```

The composite SBS score (`sesC`) correlates highly with each of the 4 constituent variables (`k1zoekl` for net income, `s1kon18` for occupational prestige, `s1kon32` for social class, and `s1kon28` for number of years of formal education). The 4 variables intercorrelate moderately to strongly, but not in excess to fear multicollinearity.

We estimated two additional survival analyses, both aimed at refining the role of the socio-biographical status composite score and of its 4 constituents (net income, occupational prestige, social class, and number of years of formal education).

First, removing SBS from the preliminary survival model does virtually not alter the estimated effects of the remaining predictors (initial age, sex, and suspected dementia; cf. Supplementary Table 2):

```
> surv2_noSBS.fit <- coxph(Surv(tstart, tstop, zmdead) ~ age1C + women + dementia,
data=ssBASE)
> summary(surv2_noSBS.fit)
Call:
coxph(formula = Surv(tstart, tstop, zmdead) ~ age1C + women +
    dementia, data = ssBASE)

  n= 1342, number of events= 516

              coef exp(coef)  se(coef)      z Pr(>|z|)
age1C     0.093847  1.098392  0.006555 14.316  < 2e-16 ***
```

```
women    -0.320451  0.725822  0.089604 -3.576 0.000348 ***
dementia  0.352353  1.422411  0.095909  3.674 0.000239 ***
---
Signif. codes:  0 ‘***’ 0.001 ‘**’ 0.01 ‘*’ 0.05 ‘.’ 0.1 ‘ ’ 1


         exp(coef) exp(-coef) lower .95 upper .95
age1C       1.0984     0.9104    1.0844    1.1126
women       0.7258     1.3777    0.6089    0.8652
dementia    1.4224     0.7030    1.1787    1.7166

Concordance= 0.727  (se = 0.012 )
Likelihood ratio test= 276.4  on 3 df,   p=<2e-16
Wald test            = 269.1  on 3 df,   p=<2e-16
Score (logrank) test = 291.4  on 3 df,   p=<2e-16
```

Moreover, removing SBS does not yield to a loss of fit:

```
> anova(surv2.fit, surv2_noSBS.fit)
Analysis of Deviance Table
 Cox model: response is  Surv(tstart, tstop, zmdead)
 Model 1: ~ age1C + women + sesC + dementia
 Model 2: ~ age1C + women + dementia
   loglik  Chisq Df Pr(>|Chi|)
1 -2572.7
2 -2572.8 0.2637  1     0.6076
```

Thus, the effect of SBS is null.

Second, a survival model with the 4 constituent variables instead of the SBS composite score does not alter the predictive effect of the other predictors:

```
> summary(surv3.fit)
Call:
coxph(formula = Surv(tstart, tstop, zmdead) ~ age1C + women +
    k1zoekl + s1kon18 + s1kon32 + s1kon28 + dementia, data = ssBASE)

  n= 1150, number of events= 428
   (192 observations deleted due to missingness)

              coef exp(coef)  se(coef)      z Pr(>|z|)
age1C     0.093771  1.098308  0.007141 13.131  < 2e-16 ***
women    -0.402871  0.668398  0.104985 -3.837 0.000124 ***
k1zoekl  -0.096019  0.908446  0.054718 -1.755 0.079290 .
s1kon18   0.089302  1.093411  0.063291  1.411 0.158253
s1kon32  -0.044084  0.956874  0.060433 -0.729 0.465716
s1kon28   0.036471  1.037144  0.065053  0.561 0.575047
dementia  0.376664  1.457414  0.110905  3.396 0.000683 ***
---
Signif. codes:  0 ‘***’ 0.001 ‘**’ 0.01 ‘*’ 0.05 ‘.’ 0.1 ‘ ’ 1

         exp(coef) exp(-coef) lower .95 upper .95
age1C       1.0983     0.9105    1.0830    1.1138
women       0.6684     1.4961    0.5441    0.8211
k1zoekl     0.9084     1.1008    0.8161    1.0113
s1kon18     1.0934     0.9146    0.9659    1.2378
s1kon32     0.9569     1.0451    0.8500    1.0772
s1kon28     1.0371     0.9642    0.9130    1.1782
dementia    1.4574     0.6861    1.1727    1.8113

Concordance= 0.727  (se = 0.013 )
Likelihood ratio test= 236.2  on 7 df,   p=<2e-16
Wald test            = 233.6  on 7 df,   p=<2e-16
Score (logrank) test = 254.6  on 7 df,   p=<2e-16
```

Hence, neither the overall composite, nor any of its constituent variables contribute to predict the hazard of dying above and beyond initial age, sex, and suspected dementia. This, however, begs the questions of whether socio-biographical status is related to survival by itself, without controlling for age, sex, and suspected dementia.

Third, we ran a survival model with only the 4 socio-biographical status variables and initial age (without which the model would be grossly misspecified):

```
> summary(surv3SBSage.fit)
Call:
coxph(formula = Surv(tstart, tstop, zmdead) ~ age1C + k1zoekl +
    s1kon18 + s1kon32 + s1kon28, data = ssBASE)

  n= 1150, number of events= 428
   (192 observations deleted due to missingness)

             coef exp(coef)  se(coef)      z Pr(>|z|)
age1C    0.097185  1.102065  0.006942 14.000   <2e-16 ***
k1zoekl -0.117602  0.889050  0.054188 -2.170   0.0300 *
s1kon18  0.146507  1.157783  0.061103  2.398   0.0165 *
s1kon32 -0.096060  0.908409  0.059623 -1.611   0.1072
s1kon28  0.051796  1.053161  0.065618  0.789   0.4299
---
Signif. codes:  0 ‘***’ 0.001 ‘**’ 0.01 ‘*’ 0.05 ‘.’ 0.1 ‘ ’ 1

        exp(coef) exp(-coef) lower .95 upper .95
age1C      1.1021     0.9074    1.0872    1.1172
k1zoekl    0.8891     1.1248    0.7995    0.9887
s1kon18    1.1578     0.8637    1.0271    1.3051
s1kon32    0.9084     1.1008    0.8082    1.0210
s1kon28    1.0532     0.9495    0.9261    1.1977

Concordance= 0.719  (se = 0.013 )
Likelihood ratio test= 209.8  on 5 df,   p=<2e-16
Wald test            = 210.1  on 5 df,   p=<2e-16
Score (logrank) test = 228.2  on 5 df,   p=<2e-16
```

This model shows that, even after controlling for initial age, net income (`k1zoekl`) and occupational prestige (`s1kon18`) predict survival. These effects appeared to be clouded by sex and suspected dementia in a more complex model (cf. the `surv3.fit`). Finally, given the importance of sex and suspected dementia revealed in predicting death, the simpler model without sex and suspected dementia provides a worse fit to the survival data:

```
> anova(surv3.fit, surv3SBSage.fit)
Analysis of Deviance Table
 Cox model: response is  Surv(tstart, tstop, zmdead)
 Model 1: ~ age1C + women + k1zoekl + s1kon18 + s1kon32 + s1kon28 + dementia
 Model 2: ~ age1C + k1zoekl + s1kon18 + s1kon32 + s1kon28
   loglik  Chisq Df Pr(>|Chi|)
1 -2051.1
2 -2064.4 26.477  2  1.781e-06 ***
```

```
---
Signif. codes:  0 ‘***’ 0.001 ‘**’ 0.01 ‘*’ 0.05 ‘.’ 0.1 ‘ ’ 1
```

References

Cox, D. R. (1972). Regression Models and Life-Tables. *Journal of the Royal Statistical Society. Series B (Methodological), 34*(2), 187–220.

Gelman, A., & Rubin, D. B. (1992). Inference from Iterative Simulation Using Multiple Sequences. *Statistical Science, 7*(4), 457–472. https://doi.org/10.1214/ss/1177011136

Kalbfleisch, J. D., & Prentice, R. L. (2002). *The statistical analysis of failure time data* (2nd ed.). Wiley-Interscience. https://doi.org/10.1002/9781118032985

Plummer, M., Best, N., Cowles, M. K., & Vines, K. (2006). CODA: Convergence Diagnosis and Output Analysis for MCMC. *R News, 6*, 7–11.

Singer, J. D., & Willett, J. B. (2003). *Applied longitudinal data analysis*. Oxford University Press. https://doi.org/10.1093/acprof:oso/9780195152968.001.0001

Therneau, T. M., Crowson, C., & Atkinson, E. (2023). Using Time Dependent Covariates and Time Dependent Coefficients in the Cox Model. Retrieved from: https://cran.r-project.org/web/packages/survival/vignettes/timedep.pdf

Widaman, K. F., & Revelle, W. (2023). Thinking thrice about sum scores, and then some more about measurement and analysis. Behavior Research Methods, 55(2), 788–806. https://doi.org/10.3758/s13428-022-01849-w

Yamaguchi, K. (1991). *Event history analysis*. Sage Publications.

Zhou, H., Hanson, T., & Zhang, J. (2020). spBayesSurv: Fitting Bayesian Spatial Survival Models Using R. Journal of Statistical Software, 92, 1–33. https://doi.org/10.18637/jss.v092.i09

Supplementary Table 1

Parameter Estimates (and Standard Errors) from Preliminary Univariate Mixed-Effect Models (N = 516)

	DS	DL	IP	PA	MT	CA	WB	VO	SW	G
Fixed Effects										
Intercept	49.22 (0.57)	49.53 (0.40)	51.28 (0.74)	50.10 (0.42)	49.86 (0.42)	50.37 (0.42)	50.40 (0.45)	49.73 (0.41)	50.84 (0.54)	50.35 (0.39)
Time in study	-0.65 (0.09)	-1.48 (0.10)	-1.48 (0.36)	-0.37 (0.09)	*-0.19 (0.10)*	-0.84 (0.10)	-0.40 (0.09)	-0.22 (0.06)	-0.81 (0.18)	-0.92 (0.10)
Time in study^2	*0.02 (0.03)*	-0.05 (0.01)	-0.12 (0.04)	*-0.01 (0.01)*	*0.01 (0.02)*	*-0.02 (0.01)*	*-0.03 (0.02)*	*0.02 (0.01)*	-0.07 (0.02)	*-0.02 (0.01)*
Initial Age	-0.62 (0.05)	-0.63 (0.04)	-0.66 (0.05)	-0.45 (0.05)	-0.42 (0.04)	-0.57 (0.42)	-0.41 (0.05)	-0.39 (0.05)	-0.26 (0.05)	-0.62 (0.04)
Initial Age x Time in study		-0.07 (0.01)	-0.05 (0.01)			-0.03 (0.01)			-0.03 (0.01)	-0.03 (0.01)
Retest$_{IPr1}$		0.55 (0.18)								
Retest$_{IA2}$						1.71 (0.33)				
Retest$_{IA3}$						1.78 (0.39)				
Retest$_{IPr3}$										
Retest$_{IA4}$						2.36 (0.46)				
Retest$_{IPr4}$							*0.86 (0.69)*	3.01 (0.59)		0.98 (0.40)
Retest$_{IA5}$										
Retest$_{IPr5}$			5.21 (1.59)	2.52 (0.82)	2.81 (0.90)			4.13 (0.72)		1.74 (0.49)
Retest$_{IA6}$						2.34 (0.81)				
Retest$_{IPr6}$			*8.26 (4.45)*						6.92 (2.06)	
Retest$_{IA7}$							*2.15 (1.39)*			
Retest$_{IPr7}$	1.87 (0.64)	2.86 (0.60)					2.85 (1.40)			
Random Effects										
Intercept	77.63	66.60	55.47	53.80	42.43	61.34	55.90	65.41	63.43	62.60
time	0.12	0.71	0.32	0.21	0.28	0.32	0.18	0.08	0.06	0.31
time^2	0.01	0.01	0.01			0.01	0.01			0.01
Residual variance	6.37	8.05	22.76	33.82	41.66	23.58	34.58	21.28	30.86	8.98
Observations	527	2236	871	963	957	1957	1046	978	899	981

Note. Time in study is in years; Time in study^2 = time in study squared, residualized from time in study, in years; Initial age is in years (at study inception); IPr = intensive protocol; IA = initial assessment; DS = digit symbol; DL = digit letter; IP = identical pictures; PA = paired associates; MT = memory for text; CA = categories; WB = word beginning; VO = vocabulary; SW = spot-a-word; G = general intelligence score; Italicized numbers correspond to statistically null effects.

Supplementary Table 2

Parameter Estimates [and 95% Confidence Intervals] from the Preliminary Survival Model

(N = 516; cf. `8_preliminary_survival.R` *on https://osf.io/u57gr/files/osfstorage)*

Predictor	Estimate	Hazard ratio
Initial Age	0.09<br>[0.08;0.11]	1.10<br>[1.08;1.11]
Sex	-0.33<br>[-0.51;-0.15]	0.72<br>[0.60;0.86]
SBS	*-0.003*<br>*[-0.01;0.01]*	*1.00*<br>*[0.99;1.01]*
Dementia	0.34<br>[0.14;0.53]	1.40<br>[1.15;1.70]

Note. Sex is coded men = 0, women = 1; SBS = Socio-biographical status; Dementia = occasion-specific assessment of suspected dementia (0 = no, 1 = yes); Hazard ratio = Estimate exponentiated; Italicized numbers correspond to rejected null hypotheses for effects at the $\alpha = .05$ level; All 516 participants died (no right-censoring).

Supplementary Table 3

Parameter Estimates [and 95% Credible Intervals] from the Multivariate Longitudinal Submodel of the final JMLSM (N = 516; cf. `2_JMLSM.R` *on https://osf.io/u57gr/files/osfstorage)*

	Multivariate Longitudinal Submodel								
	DS	DL	IP	PA	MT	CA	WB	VO	SW
Fixed Effects									
Intercept	49.00<br>[47.87;50.32]	49.49<br>[48.74;50.24]	51.02<br>[49.79;52.29]	50.14<br>[49.30;50.99]	49.78<br>[48.96;50.61]	50.59<br>[49.78;51.39]	50.44<br>[49.56;51.30]	49.77<br>[48.99;50.56]	50.46<br>[49.45;51.4]6
Time in study	–0.73<br>[–0.94;–0.51]	–1.61<br>[–1.83;–1.37]	–1.79<br>[–2.39;–1.16]	–0.57<br>[–0.78;–0.35]	–0.36<br>[–0.59;–0.15]	–1.11<br>[–1.36;–0.87]	–0.66<br>[–0.90;–0.43]	–0.35<br>[–0.49;–0.21]	–0.91<br>[–1.25;–0.54]
Time in study^2	*–0.02*<br>*[–0.10;0.05]*	–0.06<br>[–0.08;–0.04]	–0.13<br>[–0.21;–0.06]	*–0.01*<br>*[–0.04;0.02]*	*0.01*<br>*[–0.02;0.04]*	–0.03<br>[–0.06;–0.01]	*–0.03*<br>*[–0.06;0.01]*	*0.02*<br>*[–0.01;0.04]*	–0.06<br>[–0.10;–0.01]
Initial age	–0.67<br>[–0.76;–0.59]	–0.64<br>[–0.72;–0.56]	–0.69<br>[–0.78;–0.60]	–0.46<br>[–0.55;–0.37]	–0.43<br>[–0.52;–0.35]	–0.56<br>[–0.64;–0.47]	–0.43<br>[–0.52;–0.33]	–0.40<br>[–0.49;–0.31]	–0.28<br>[–0.38;–0.18]
Initial age x Time in study		–0.07<br>[–0.09;–0.05]	–0.06<br>[–0.09;–0.03]			–0.03<br>[–0.06;–0.01]			–0.04<br>[–0.06;–0.01]
Retest$_{IPr1}$		0.57<br>[0.23;0.91]							
Retest$_{IA2}$						1.64<br>[1.00;2.27]			
Retest$_{IA3}$						1.69<br>[0.93;2.45]			
Retest$_{IPr3}$									
Retest$_{IA4}$						2.33<br>[1.45;3.20]			
Retest$_{IPr4}$							*0.99*<br>*[–0.30;2.27]*	2.96<br>[1.84;4.09]	
Retest$_{IA5}$									
Retest$_{IPr5}$			4.95<br>[2.53;7.74]	2.51<br>[0.96;4.06]	2.76<br>[1.00;4.53]			4.14<br>[2.79;5.50]	
Retest$_{IA6}$						2.31<br>[0.77;3.87]			
Retest$_{IPr6}$			7.63<br>[1.14;15.65]						5.84<br>[1.74;9.71]
Retest$_{IA7}$							*1.71*<br>*[–0.78;4.22]*		

Retest$_{IPr7}$	1.83 [0.51;3.16]	2.76 [1.57;3.95]					*2.36 [–0.13;4.91]*		
Random Effects									
Intercept	58.50 [44.76;76.39]	56.74 [49.61;63.69]	49.73 [39.13;60.58]	51.73 [43.82;60.74]	38.40 [31.05;47.02]	53.94 [46.94;61.67]	53.81 [45.45;62.68]	60.56 [52.78;69.30]	59.03 [49.10;69.60]
Time in study	0.12 [0.08;0.17]	0.58 [0.40;0.82]	0.37 [0.14;0.80]	0.27 [0.14;0.51]	0.27 [0.13;0.47]	0.45 [0.31;0.63]	0.40 [0.23;0.65]	0.12 [0.05;0.22]	0.13 [0.04;0.29]
Time in study^2	0.01 [0.01;0.03]	0.01 [0.01;0.01]	0.01 [0.01;0.01]			0.01 [0.01;0.01]	0.01 [0.01;0.01]		
Residual variance	6.92 [4.73;10.12]	8.05 [7.47;8.67]	18.71 [15.77;21.96]	32.27 [28.25;36.94]	41.08 [35.90;46.85]	22.66 [20.99;24.50]	31.52 [27.81;35.65]	19.91 [17.42;22.72]	29.36 [25.42;33.82]
Observations	527	2236	871	963	957	1957	1046	978	899

Note. Time in study is in years; Time in study^2 = time in study squared, residualized from time in study, in years; Initial age is in years (at study inception); IPr = intensive protocol; IA = initial assessment; DS = digit symbol; DL = digit letter; IP = identical pictures; PA = paired associates; MT = memory for text; CA = categories; WB = word beginning; VO = vocabulary; SW = spot–a–word; Italicized numbers correspond to effects with a 95% credible interval that includes 0 (null effects).

Supplementary Table 4

Intercorrelations of Random Effects from the Multivariate Longitudinal Submodel of the final JMLSM (N = 516; cf. `2_JMLSM.R` *on https://osf.io/u57gr/files/osfstorage)*

		Digit symbol			Digit letter			Identical pictures			Paired associates		Memory for text		Category			Word beginning			Vocabulary		Spot-a-word
		Int.	t	rt2	Int.	t	rt2	Int.	t	rt2	Int.	t	Int.	t	Int.	t	rt2	Int.	t	rt2	Int.	t	Int.
DS	t	.08																					
	rt2	-.09	-.18																				
DL	Int.	.82	-.01	.16																			
	t	.01	.29	.03	-.06																		
	rt2	-.05	.09	-.06	-.18	-.08																	
IP	Int.	.73	.05	.11	.78	.10	-.21																
	t	-.01	.27	-.01	-.07	.62	.08	-.11															
	rt2	-.04	-.05	.03	-.09	-.34	.41	-.27	.08														
PA	Int.	.47	.01	.01	.43	.10	-.16	.44	.06	-.06													
	t	.13	.17	.06	.10	.37	.04	.13	.21	-.08	.14												
MT	Int.	.40	-.05	.09	.41	.05	.04	.42	.07	.08	.66	.10											
	t	-.02	.10	.09	.00	.23	-.06	-.03	.16	.02	-.08	.13	-.22										
CA	Int.	.54	.02	.18	.57	.12	-.14	.58	.06	.01	.50	.17	.56	.05									
	t	.09	.12	.03	.06	.59	-.04	.05	.34	-.10	.06	.32	.01	.38	.03								
	rt2	-.16	.02	-.05	-.24	-.11	.33	-.16	-.05	.15	-.22	-.02	-.10	-.07	-.35	.04							
WB	Int.	.60	.05	.14	.60	.07	-.15	.55	.02	.03	.51	.13	.50	-.02	.76	.03	-.20						
	t	-.11	.28	.03	-.15	.54	-.03	-.12	.38	-.10	-.02	.36	-.07	.30	-.02	.48	.05	-.04					
	rt2	.11	.10	-.09	-.02	.01	.21	.05	.06	.04	-.08	.07	.00	-.11	-.10	-.07	.29	-.09	.03				
VO	Int.	.53	.10	.10	.49	-.01	-.05	.54	-.08	.01	.50	.17	.53	-.03	.64	-.01	-.12	.61	-.09	.08			
	t	-.02	.13	.10	.01	.44	-.01	.09	.26	-.10	.12	.31	.20	.20	.19	.31	-.05	.12	.31	-.01	.08		
SW	Int.	.46	.08	.11	.45	.04	.05	.48	-.03	-.01	.39	.16	.41	-.01	.41	.05	-.01	.49	-.07	.08	.77	.15	
	t	.14	.08	-.02	.13	.42	-.09	.14	.33	-.22	.15	.16	.06	.09	.03	.23	-.07	.00	.20	.07	-.13	.21	-.13

Note. DS = digit symbol; DL = digit letter; IP = identical pictures; PA = paired associates; MT = memory for text; CA = categories; WB = word beginning; VO = vocabulary; SW = spot-a-word; Int. = Intercept; t = time in study, in years; rt2 = time in study squared, residualized from time in study, in years.

Supplementary Figure 1

Trace Plots of Survival Parameters (cf. `2_JMLSM.R` *on https://osf.io/u57gr/files/osfstorage)*

Initial Age

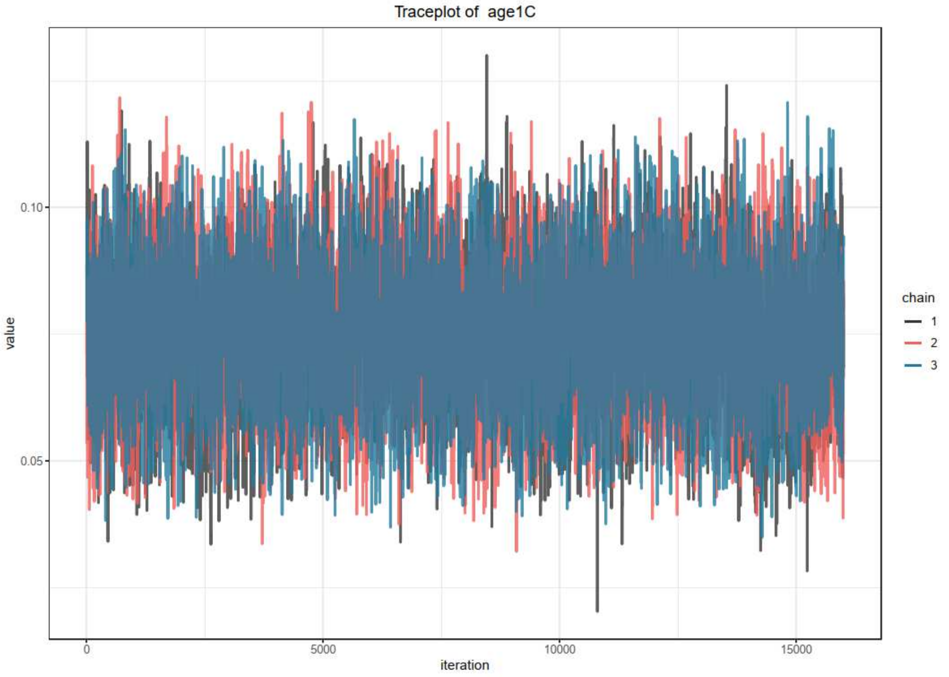

Sex

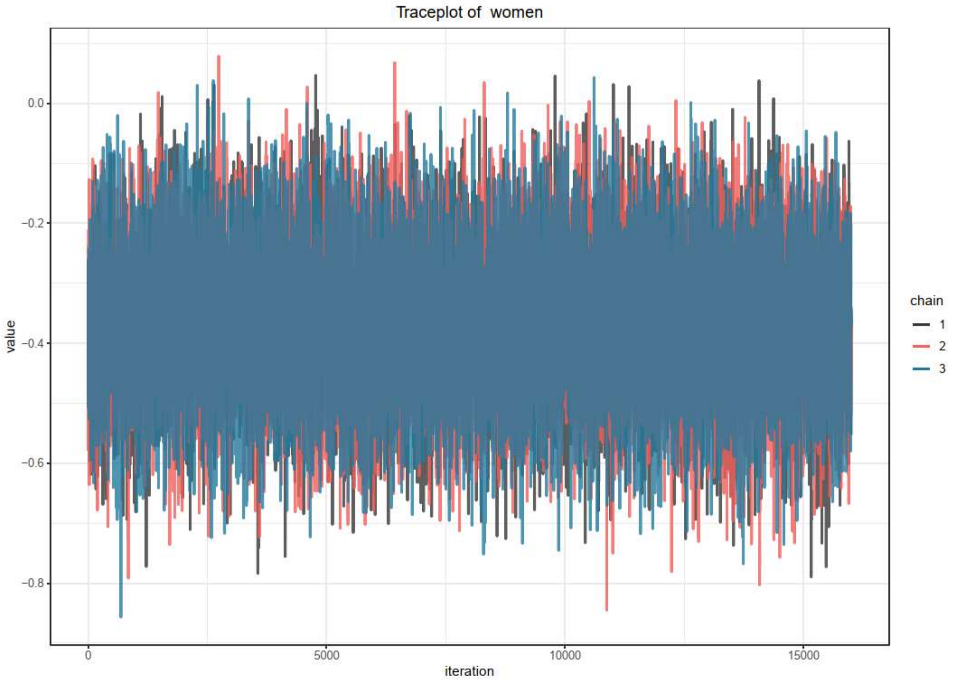

Socio-Biographical Status

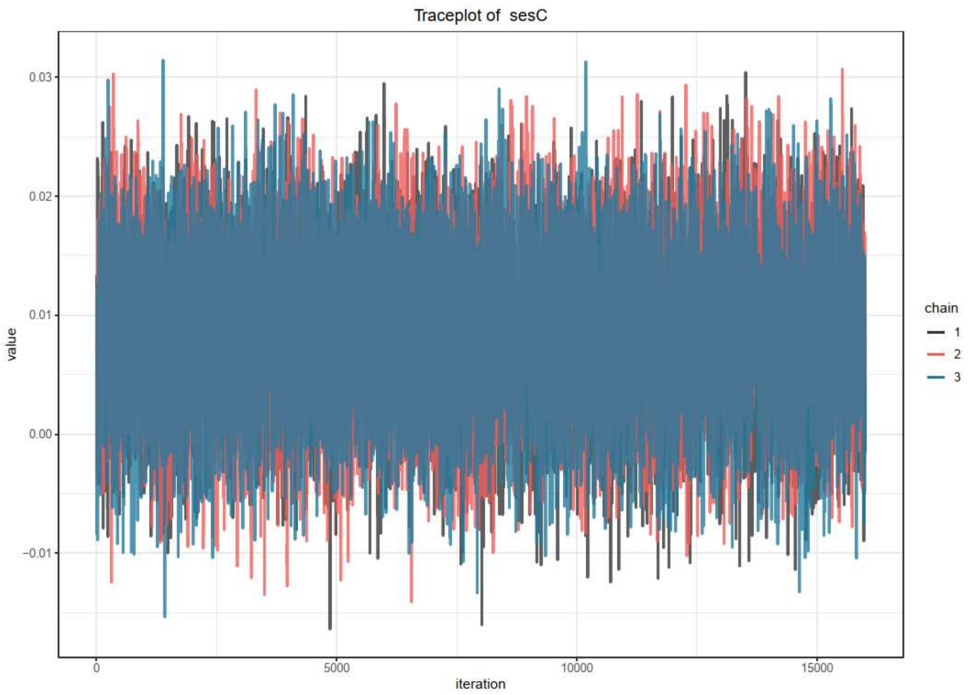

Suspected Dementia

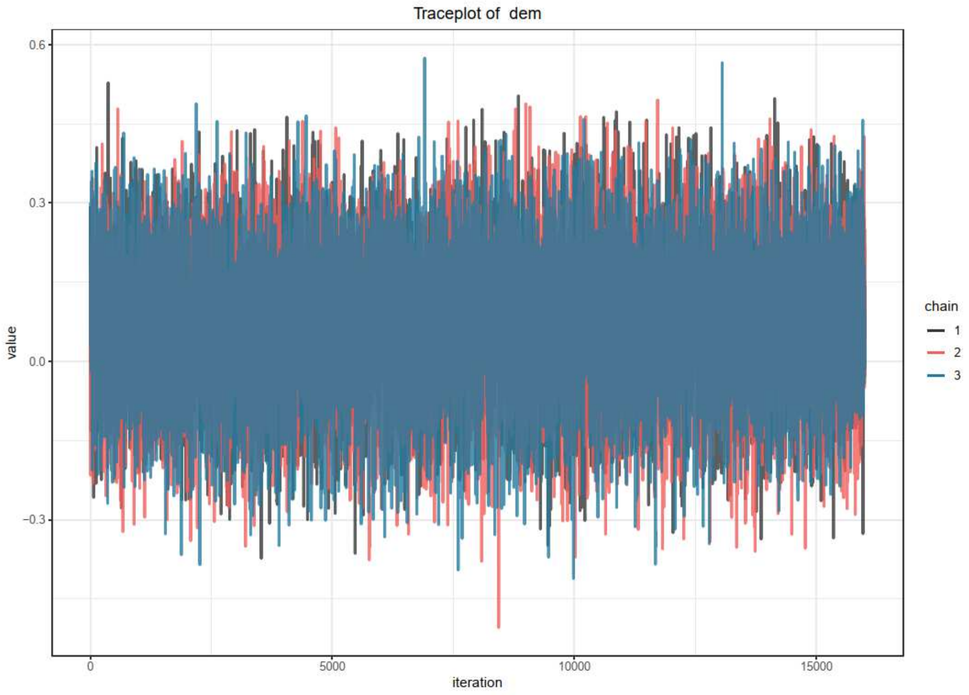

Association Parameters (α_1 and α_2) for Digit Symbol

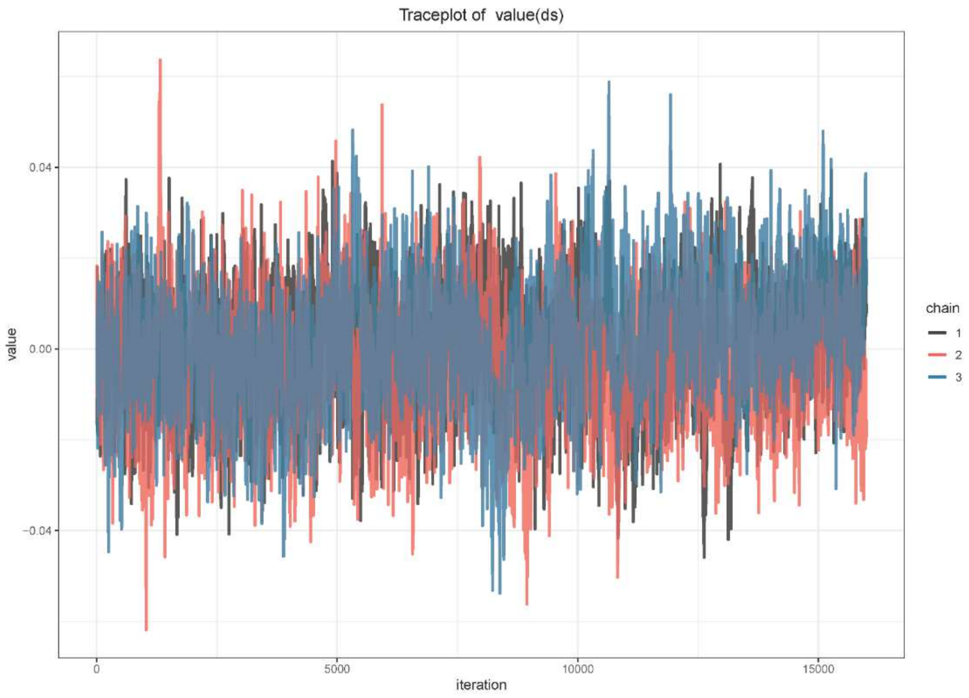

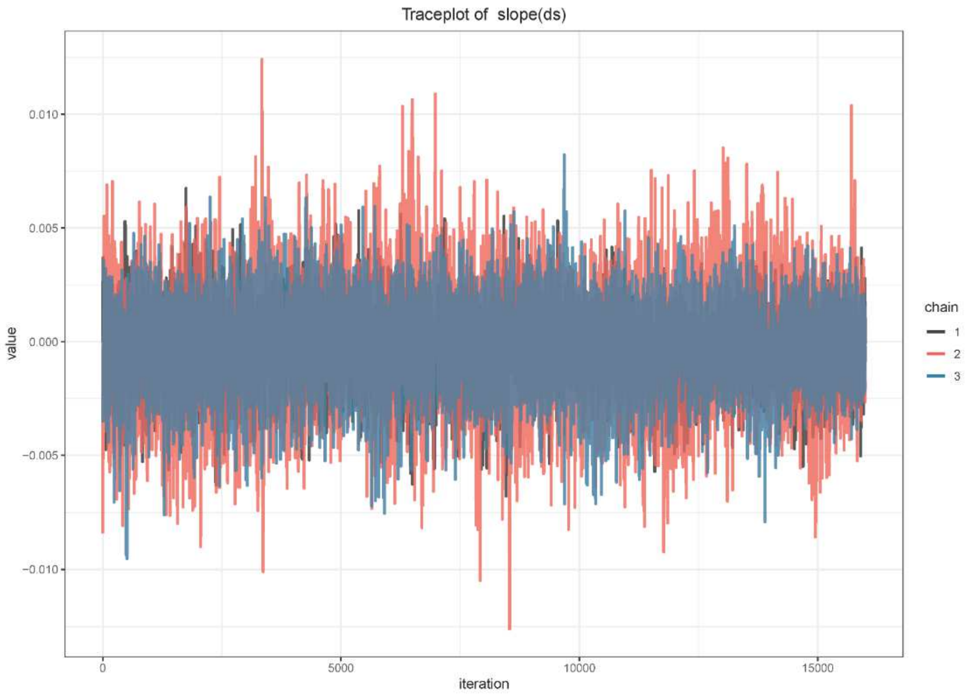

Association Parameters (α_1 and α_2) for Digit Letter

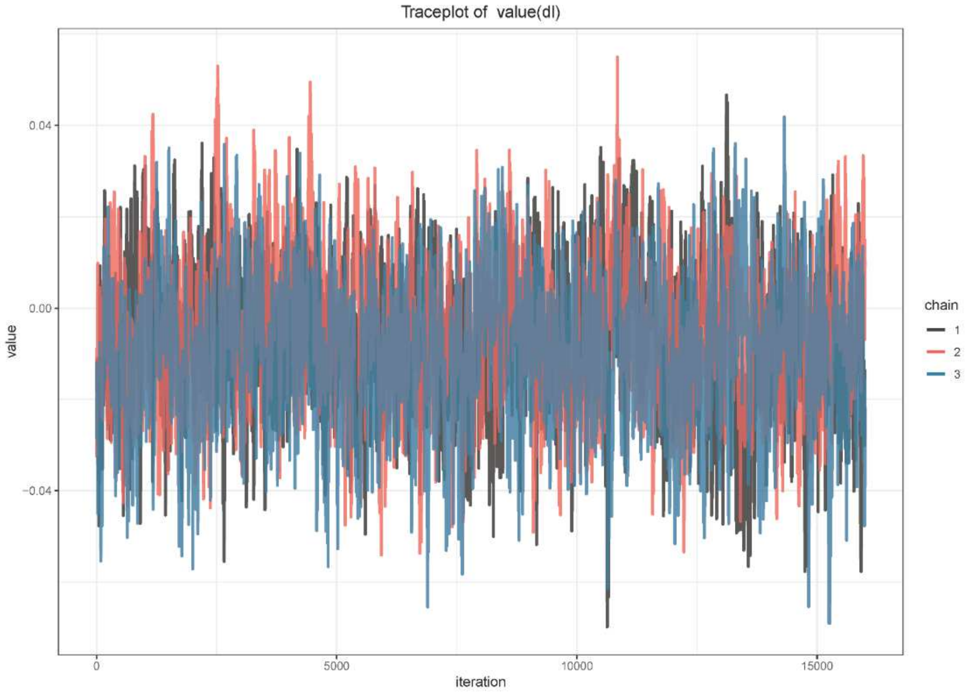

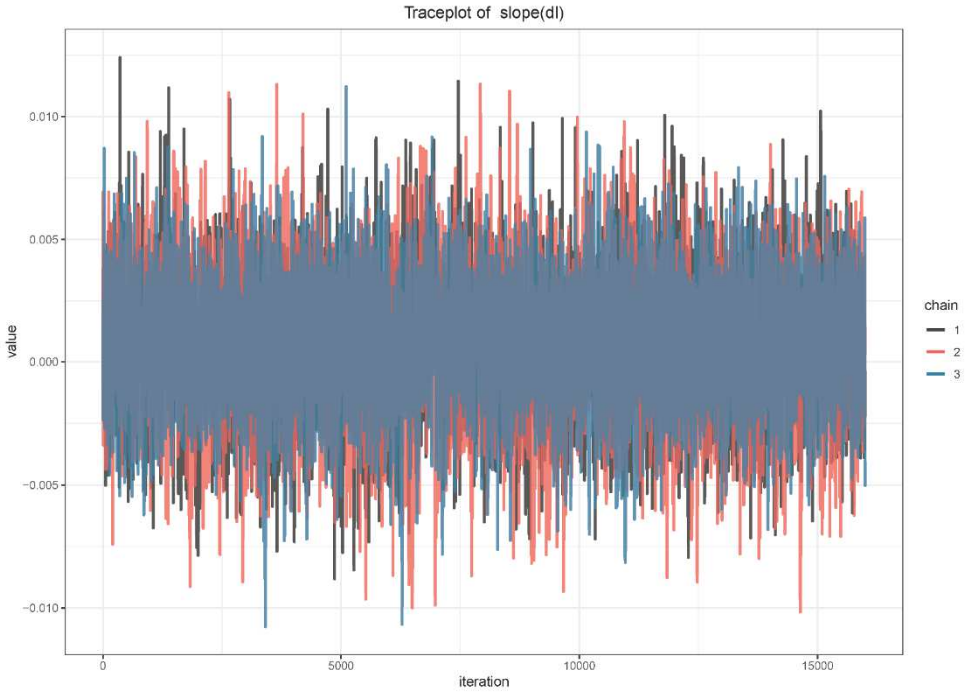

Association Parameters (α_1 and α_2) for Identical Pictures

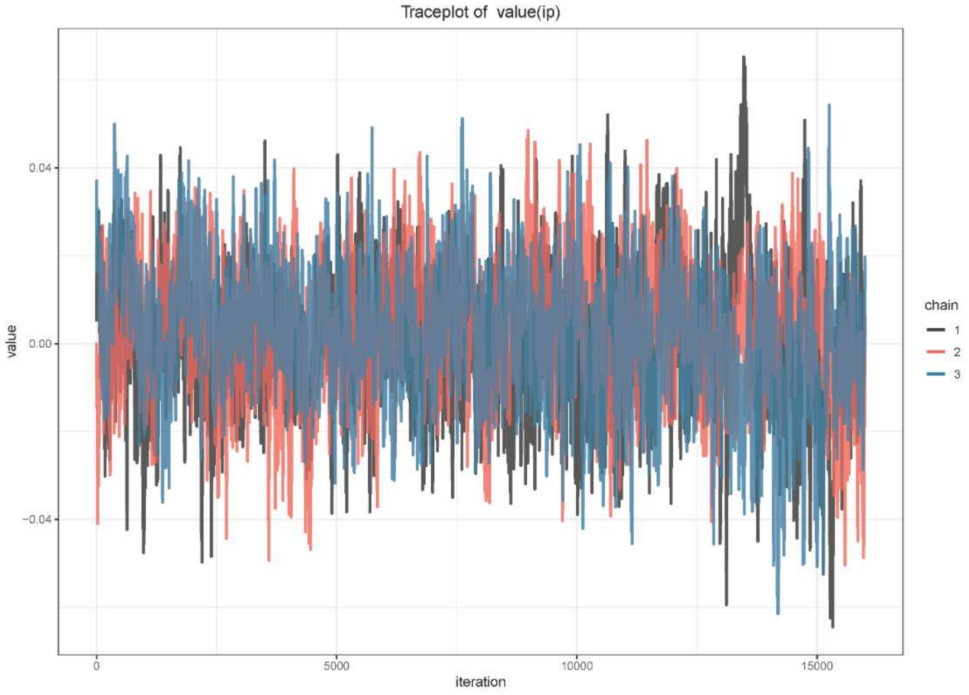

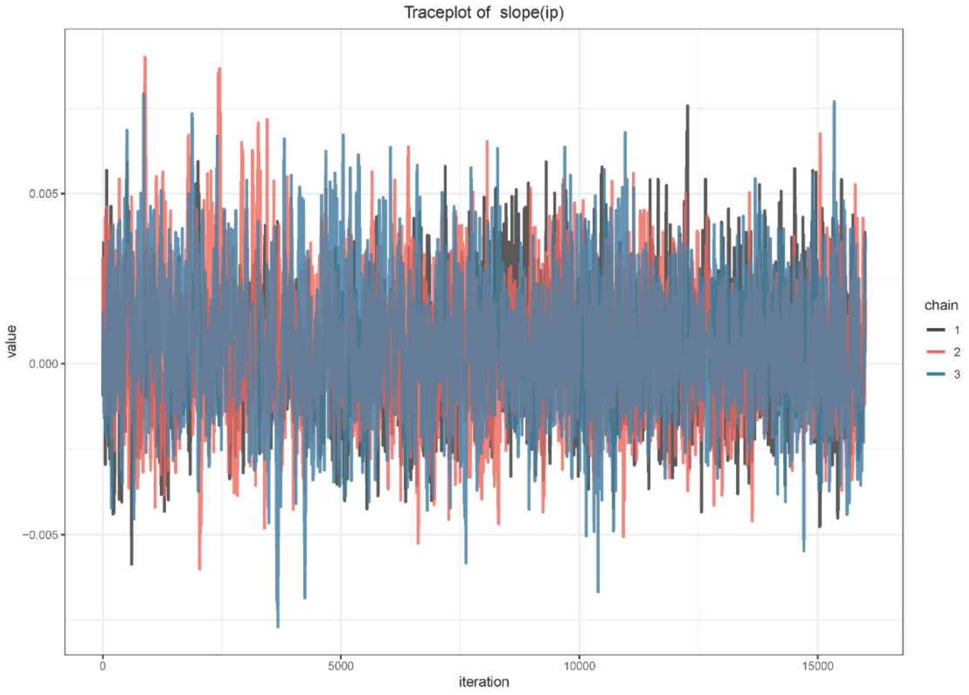

Association Parameters (α_1 and α_2) for Paired Associates

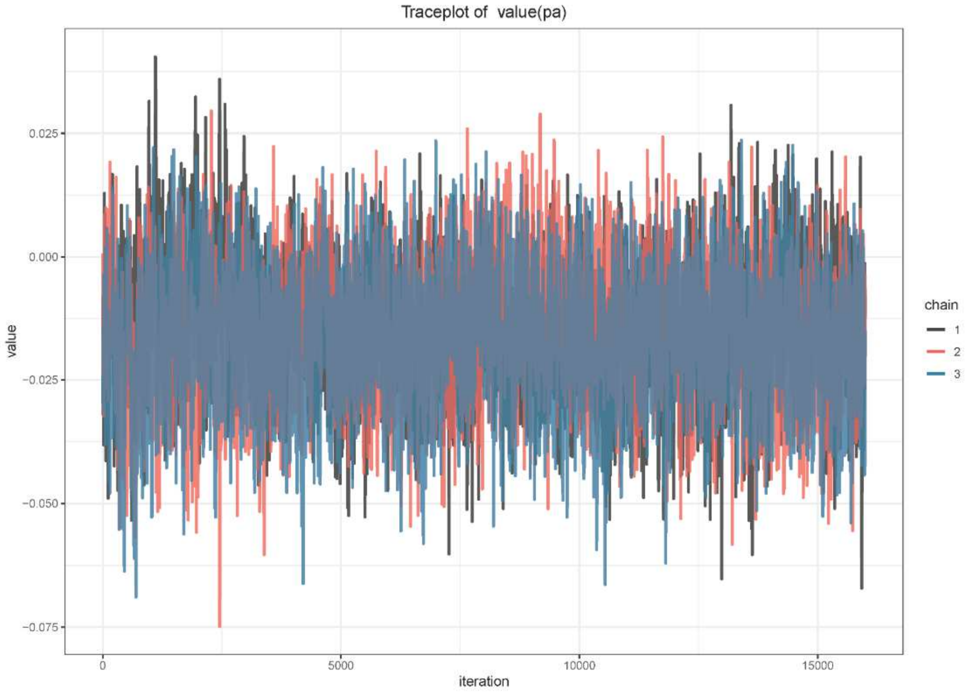

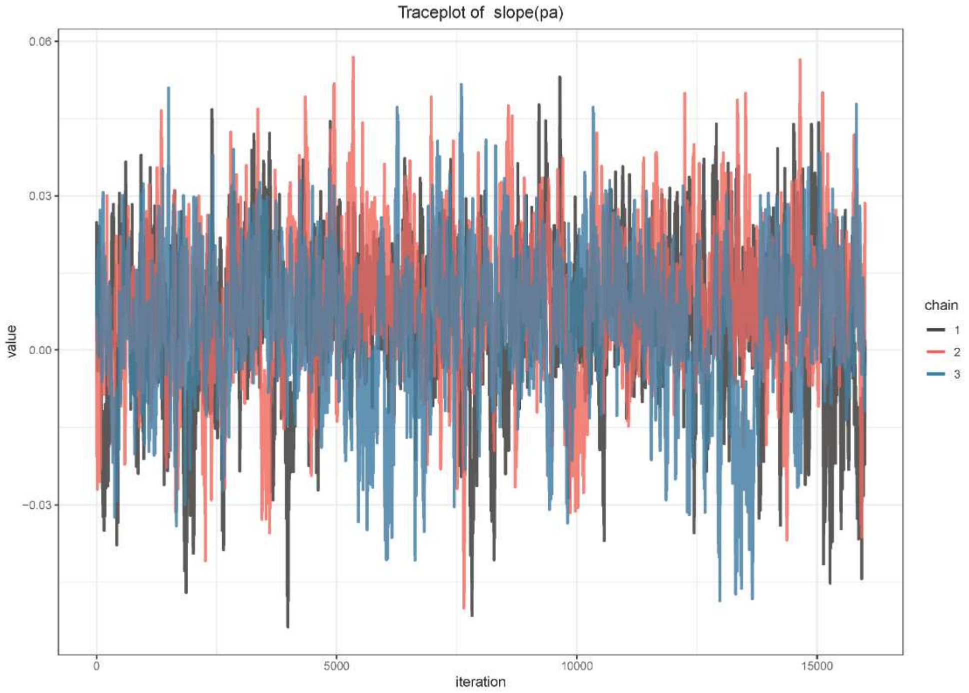

Association Parameters (α_1 and α_2) for Memory for Text

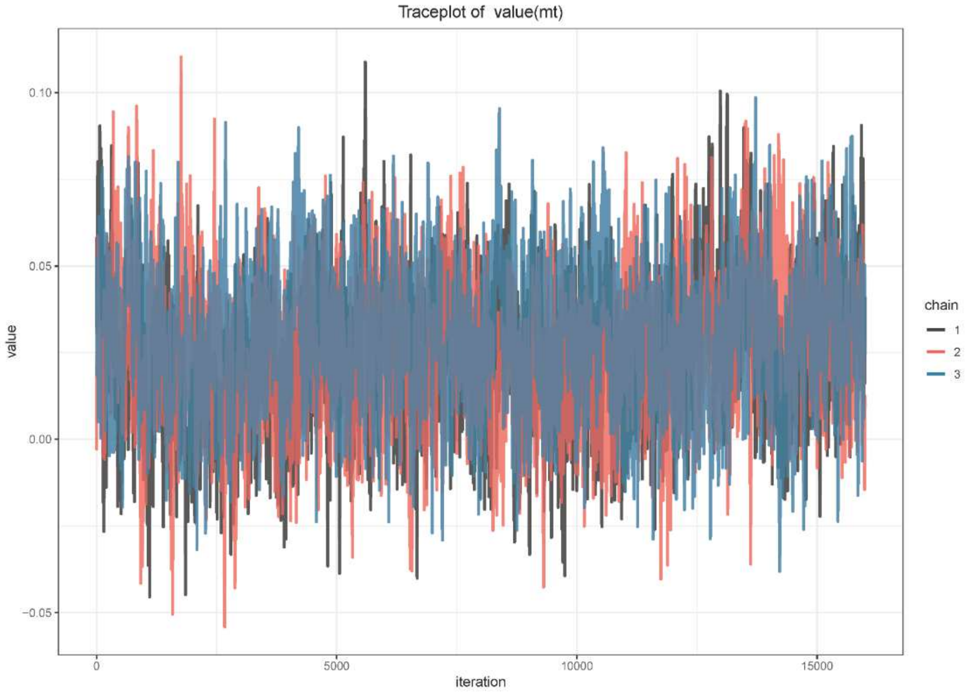

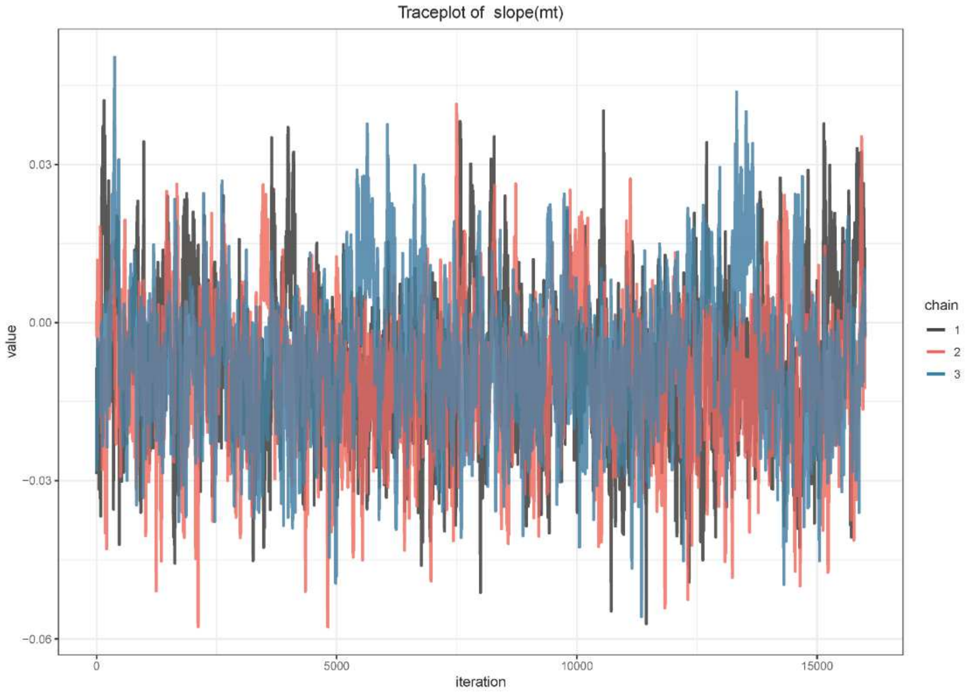

Association Parameters (α_1 and α_2) for Categories

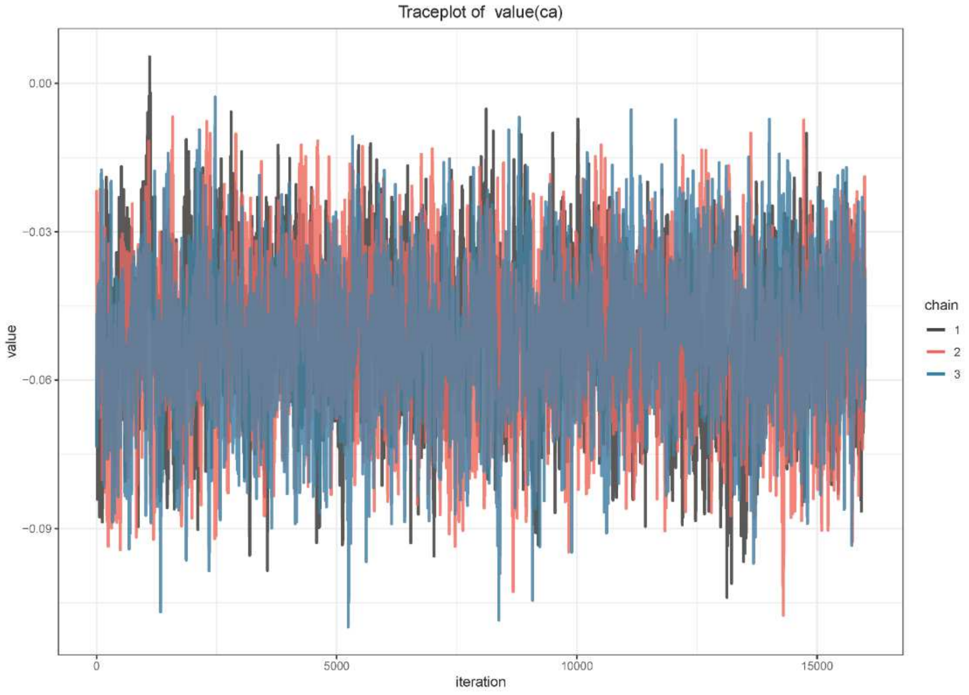

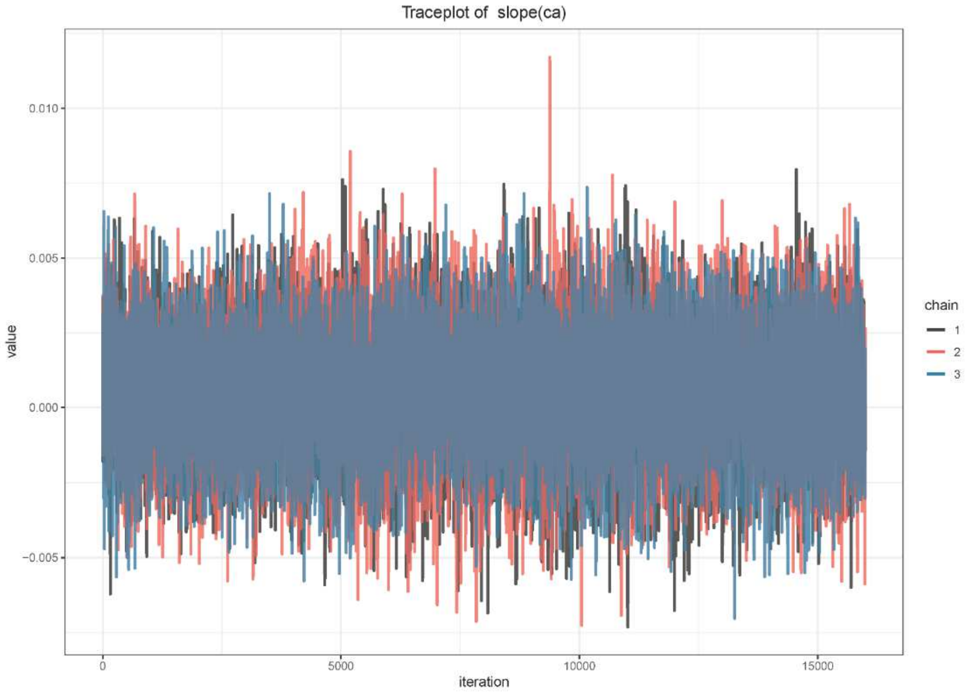

Association Parameters (α_1 and α_2) for Word Beginning

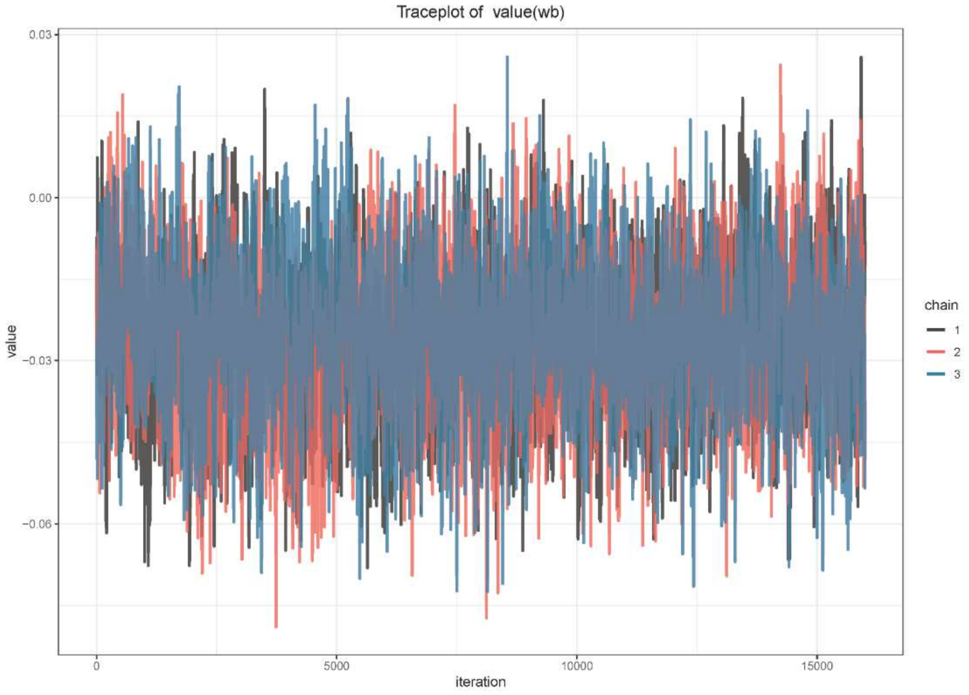

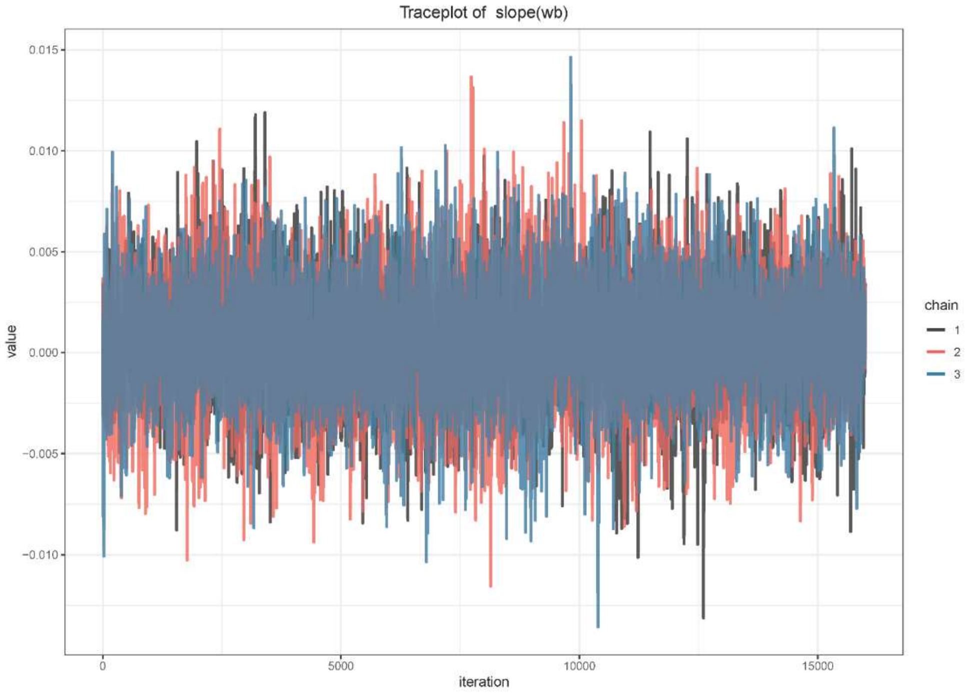

Association Parameters (α_1 and α_2) for Vocabulary

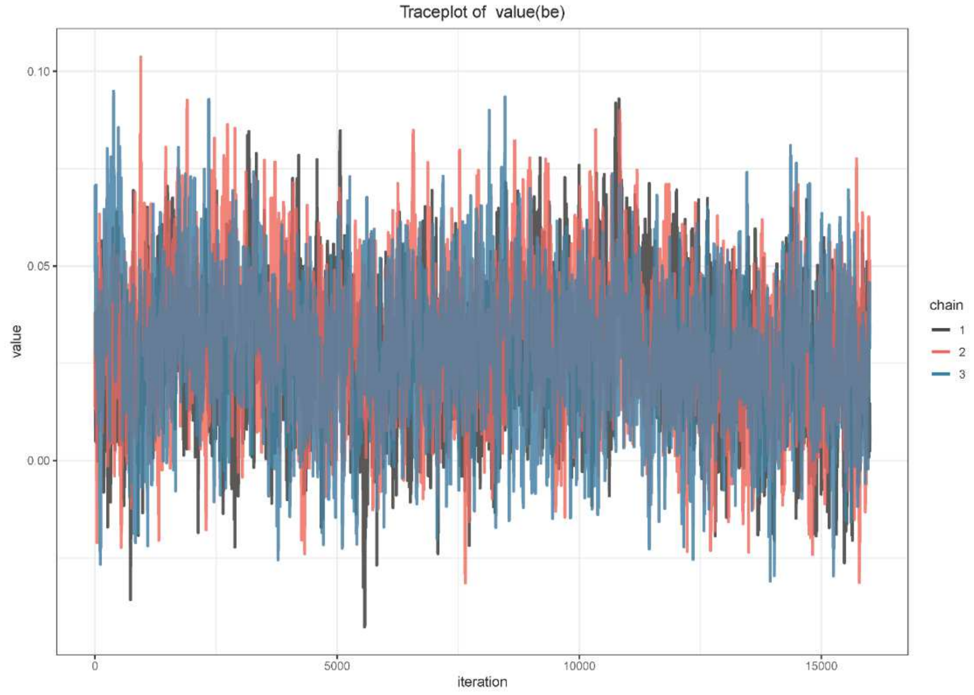

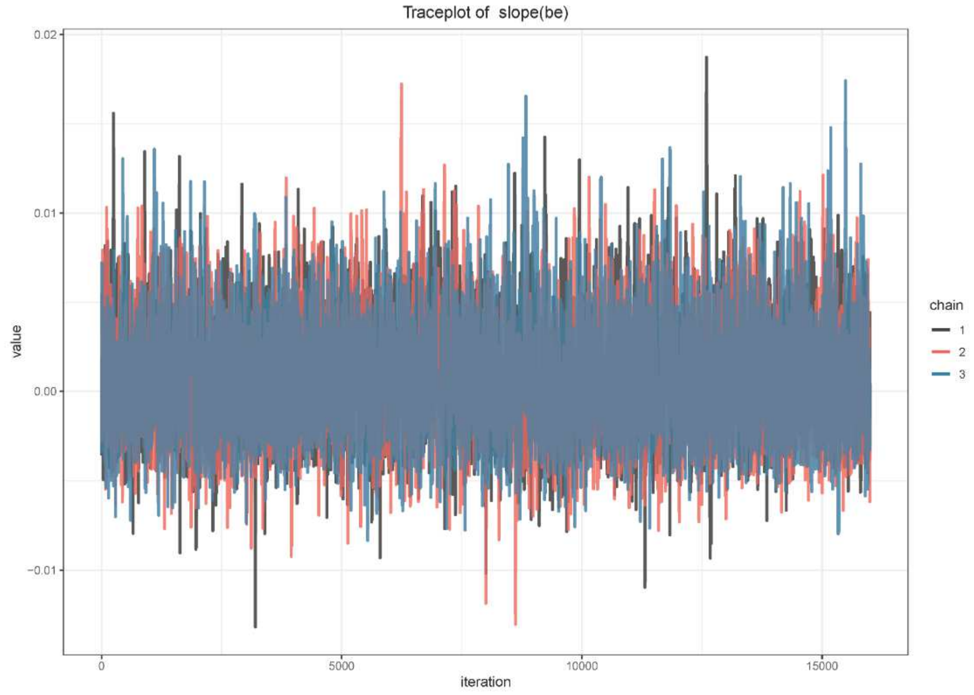

Association Parameters (α_1 and α_2) for Spot-a-Word

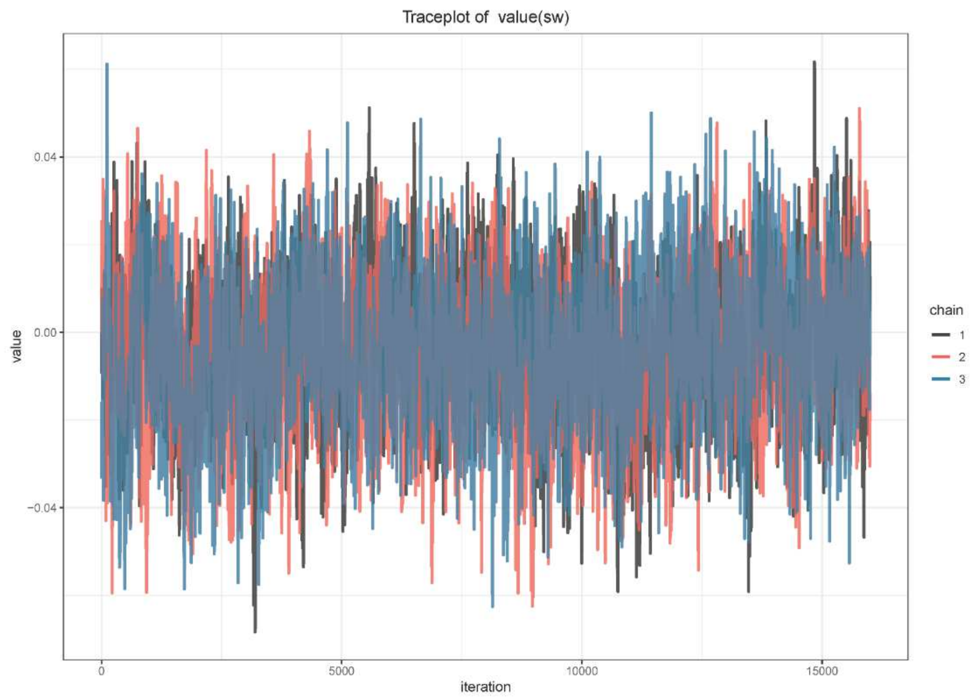

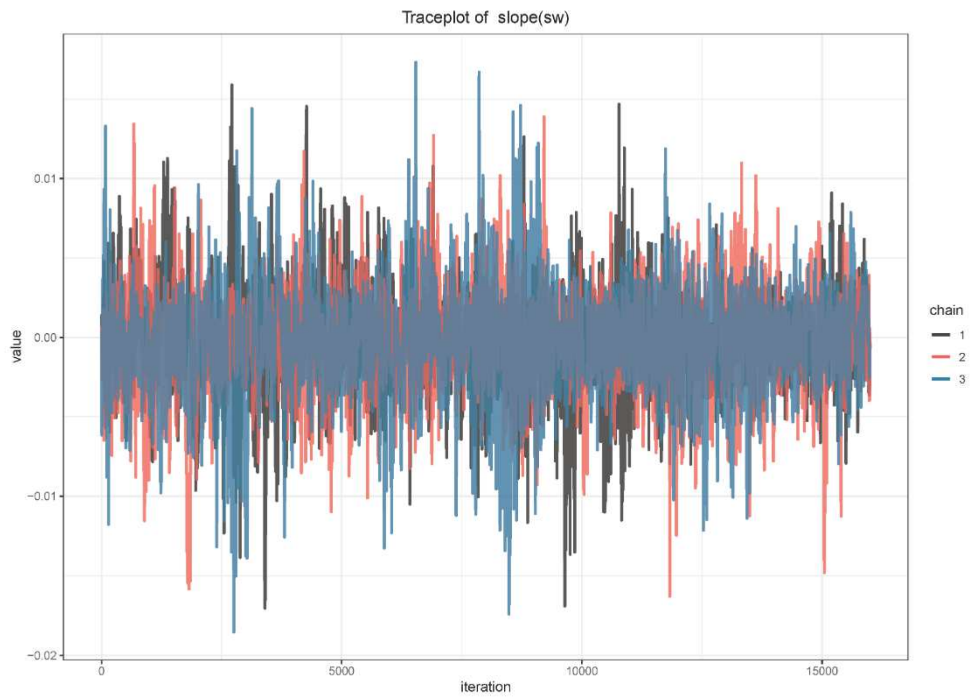

Supplementary Figure 2

Posterior Density Plots of Survival Parameters (cf. `2_JMLSM.R` *on* *https://osf.io/u57gr/files/osfstorage*)

Initial Age

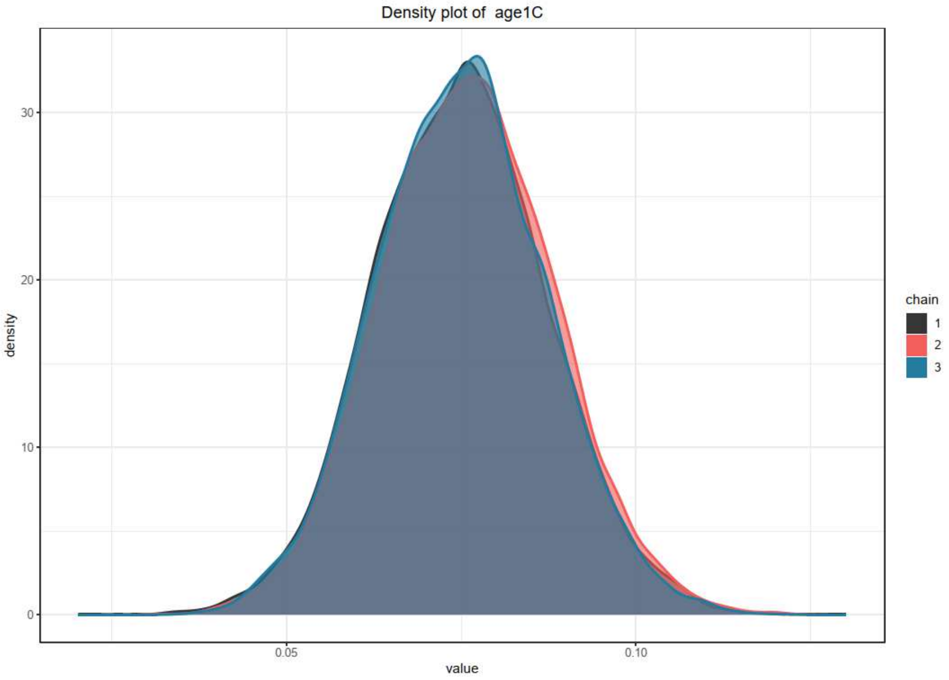

Sex

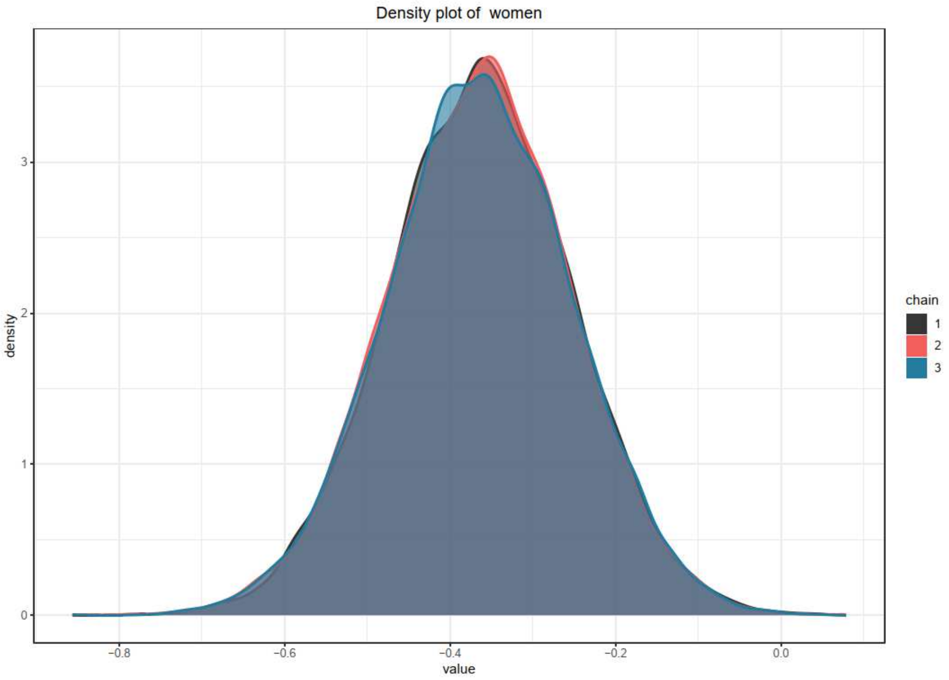

Socio-Biographical Status

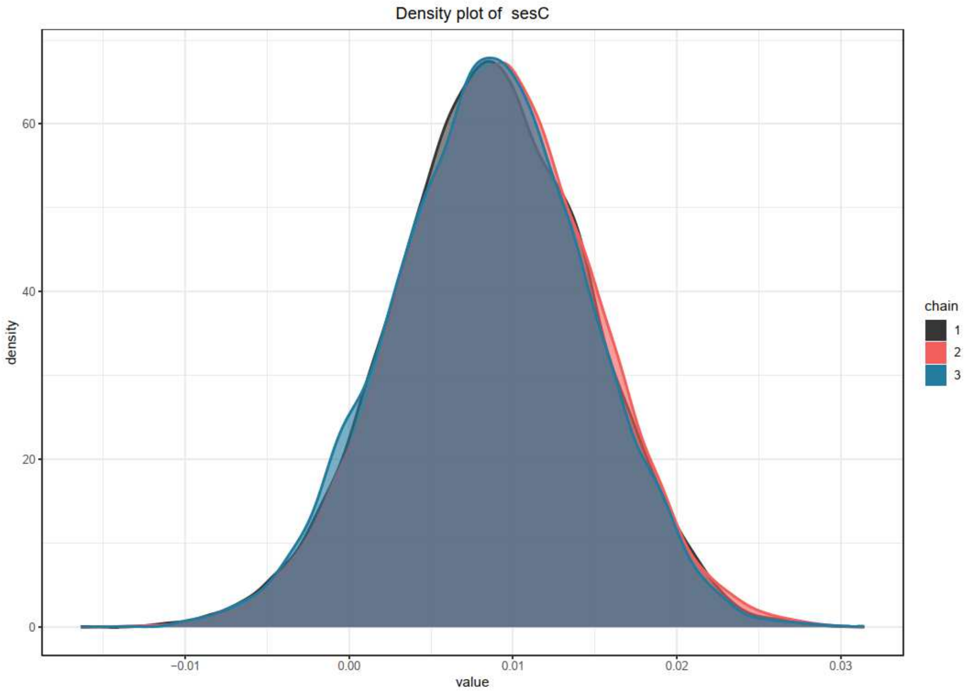

Suspected Dementia

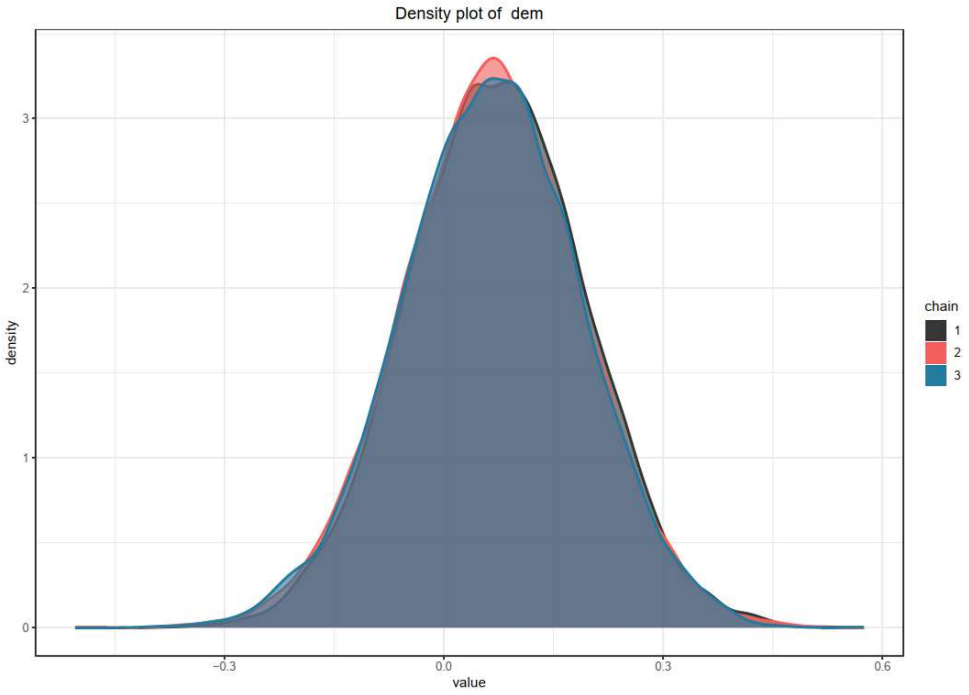

Association Parameters (α_1 and α_2) for Digit Symbol

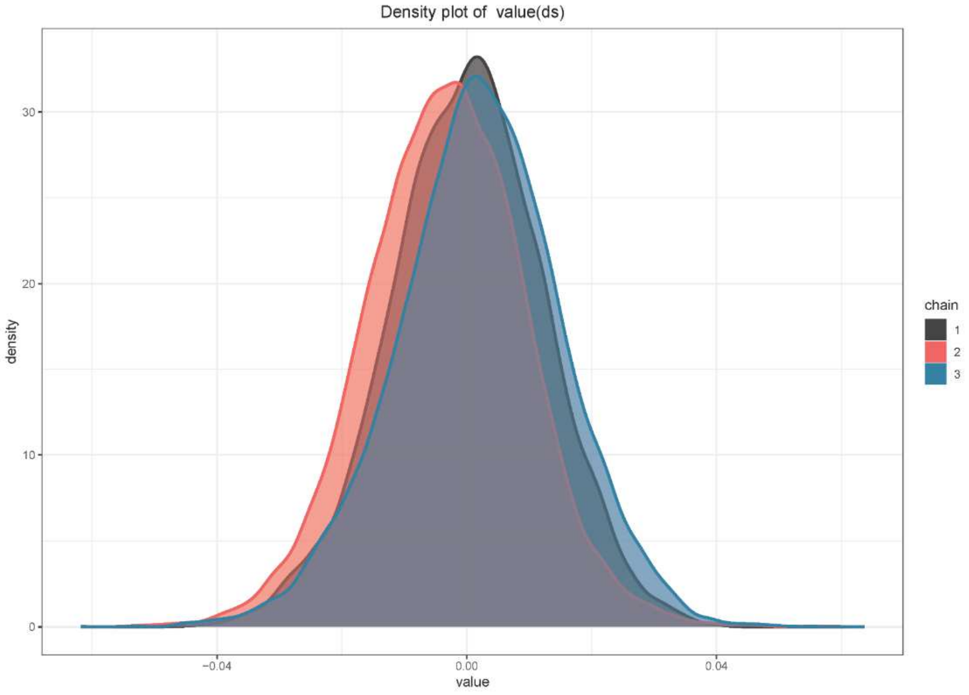

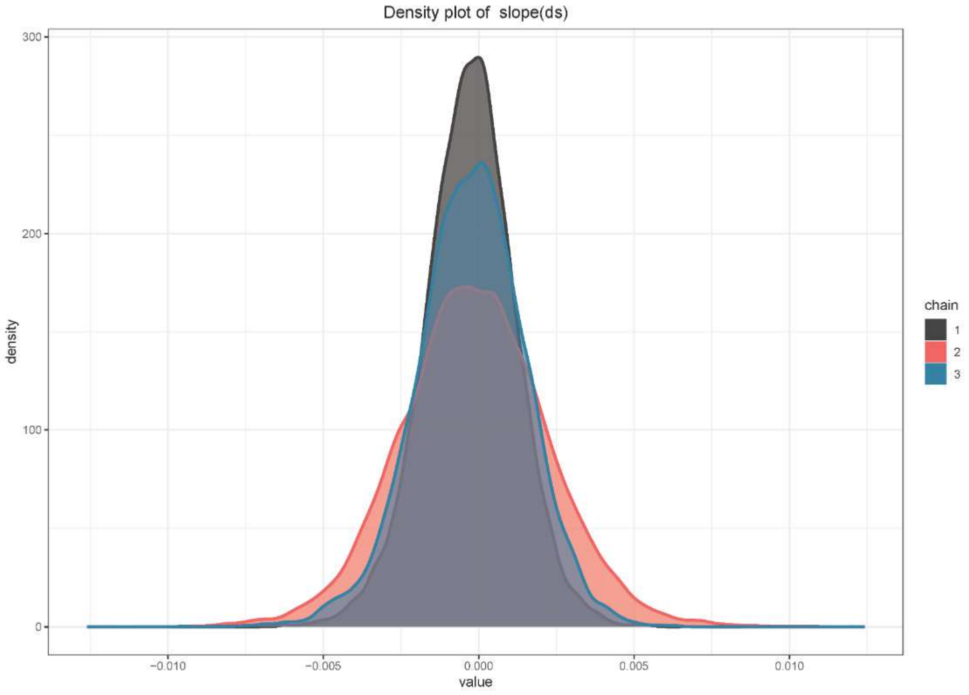

Association Parameters (α_1 and α_2) for Digit Letter

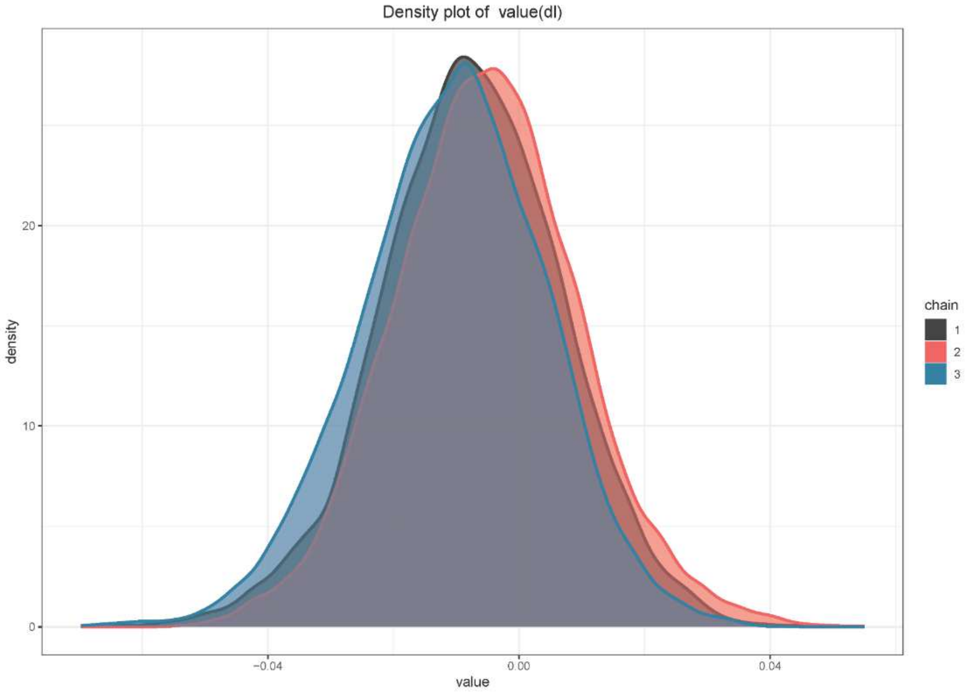

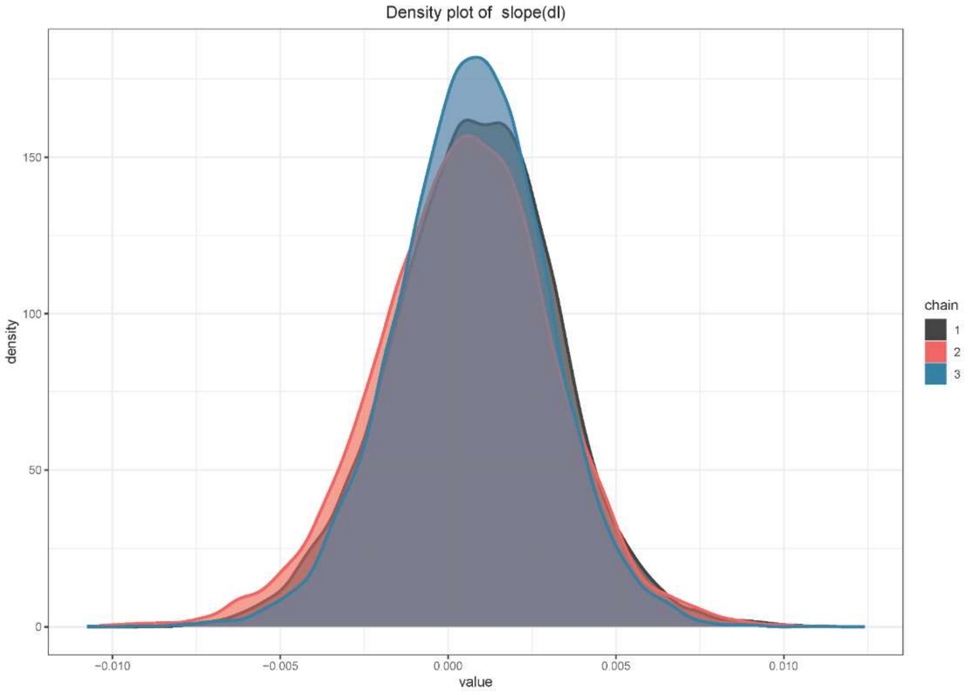

Association Parameters (α_1 and α_2) for Identical Pictures

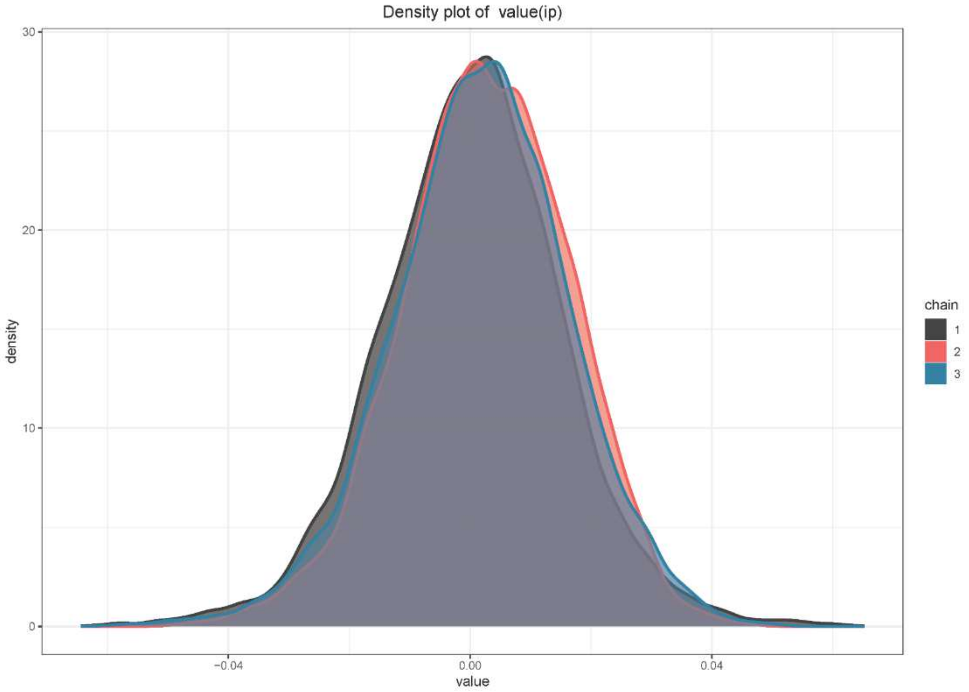

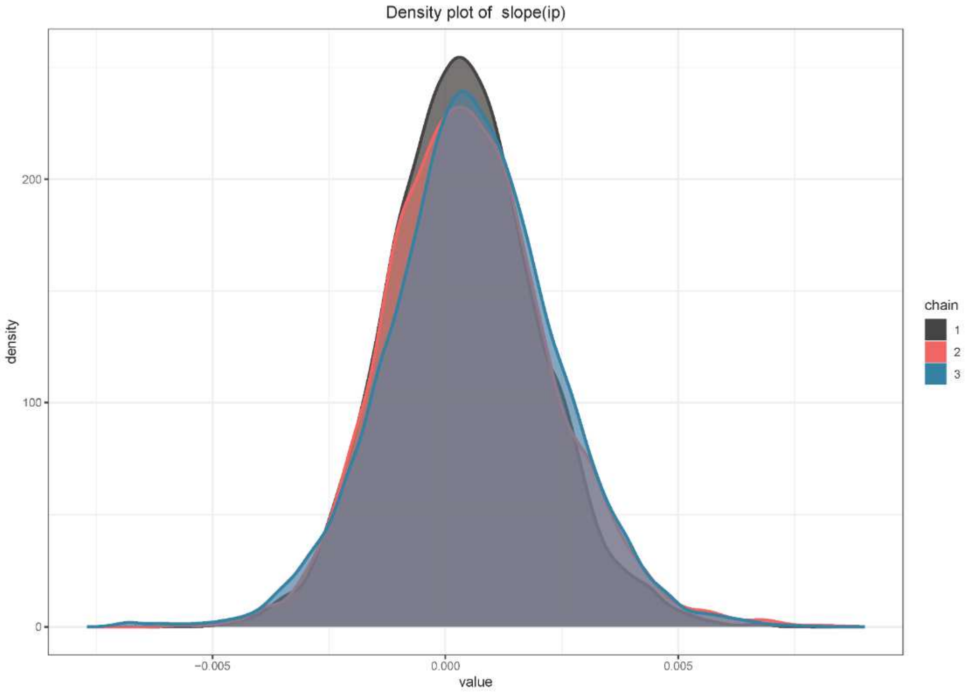

Association Parameters (α_1 and α_2) for Paired Associates

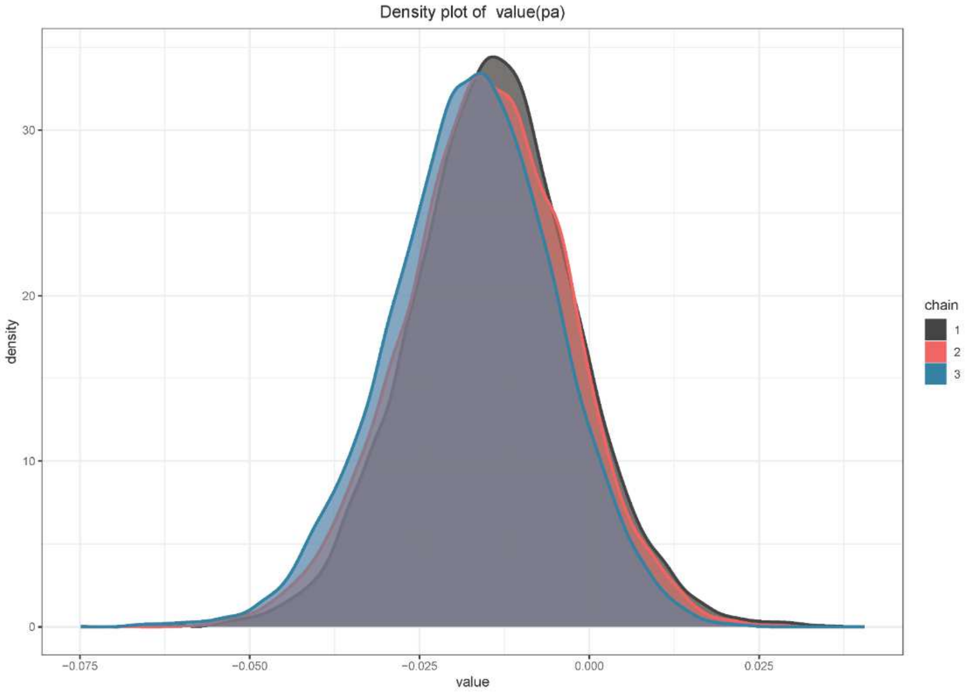

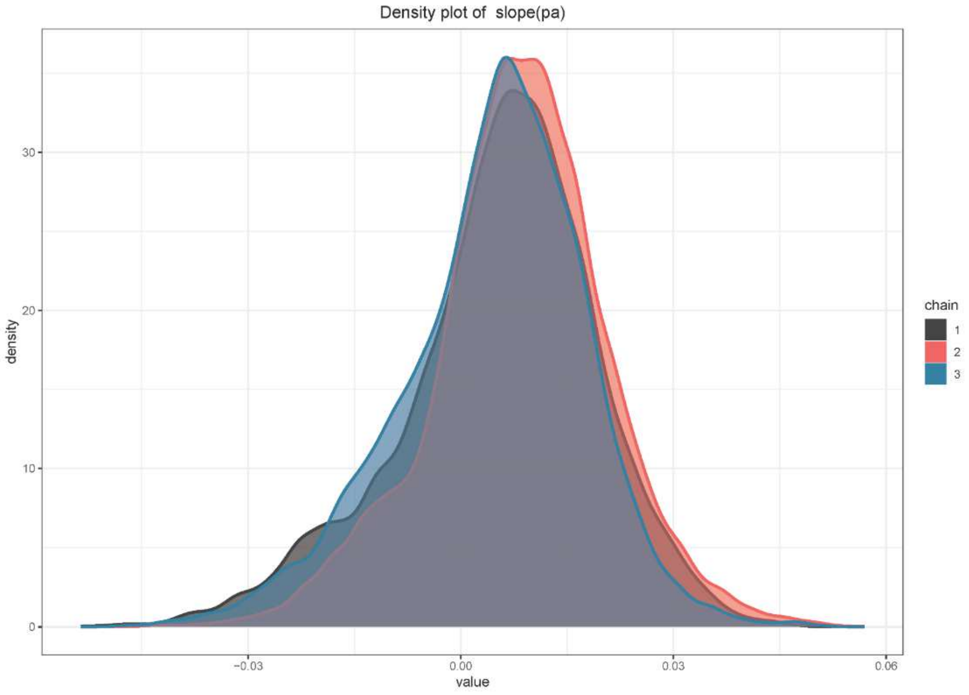

Association Parameters (α_1 *and* α_2*) for Memory for Text*

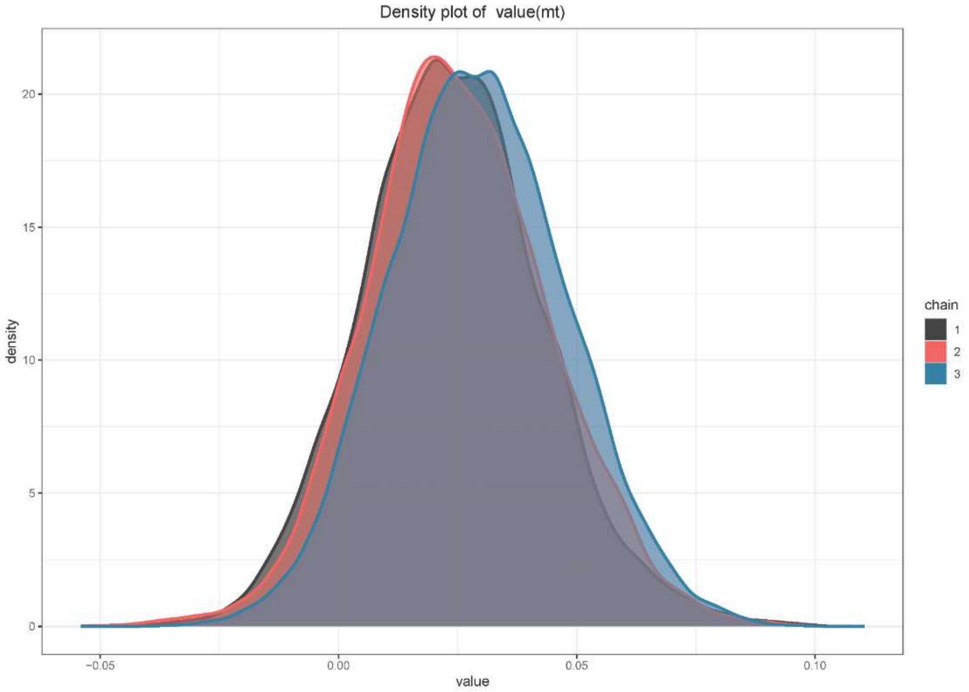

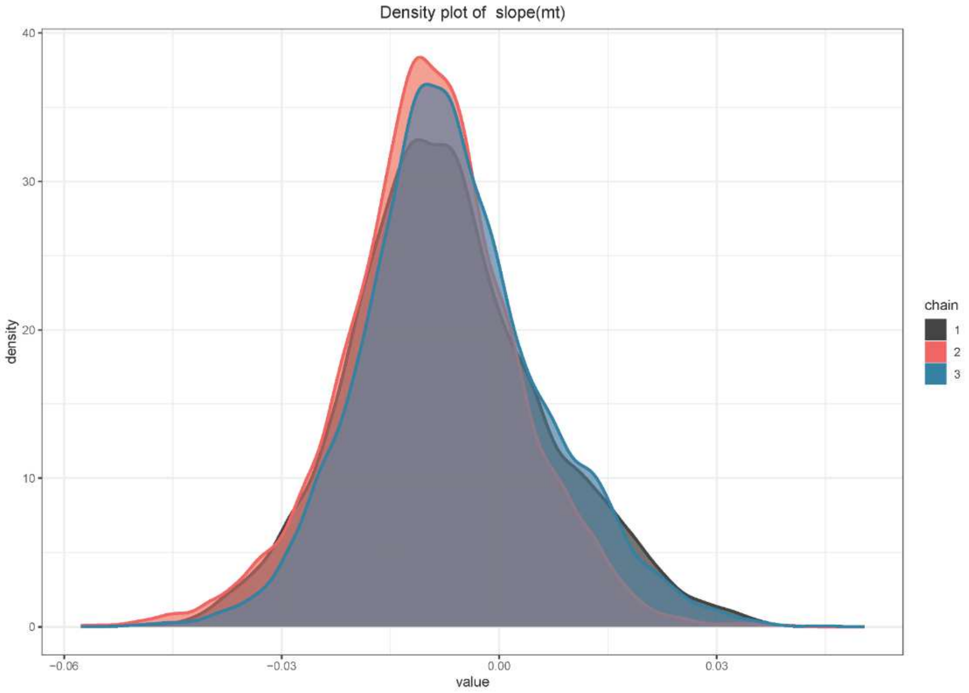

Association Parameters (α_1 and α_2) for Categories

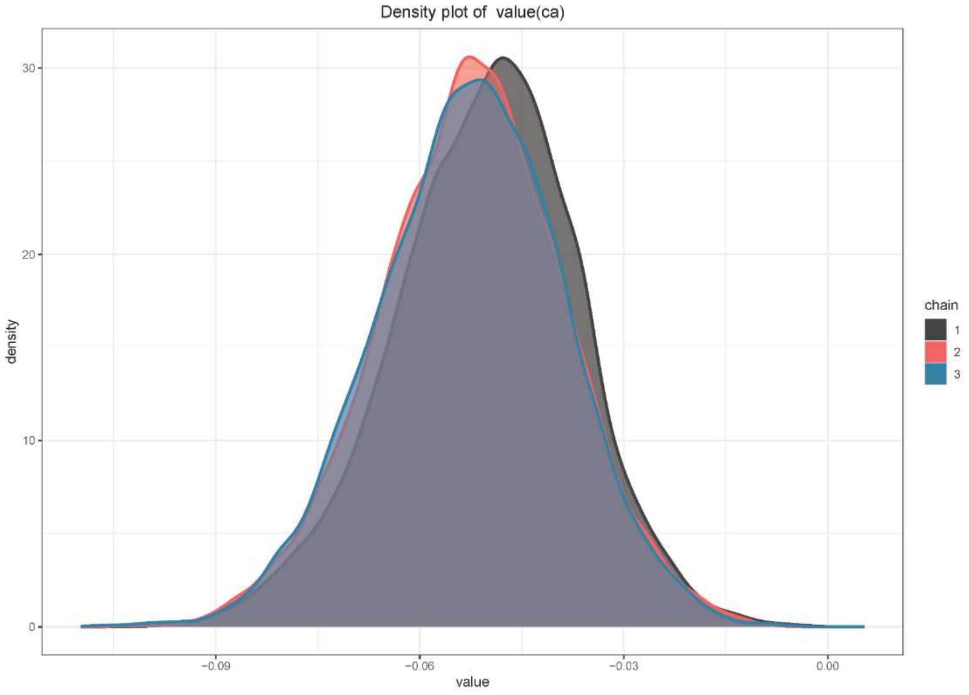

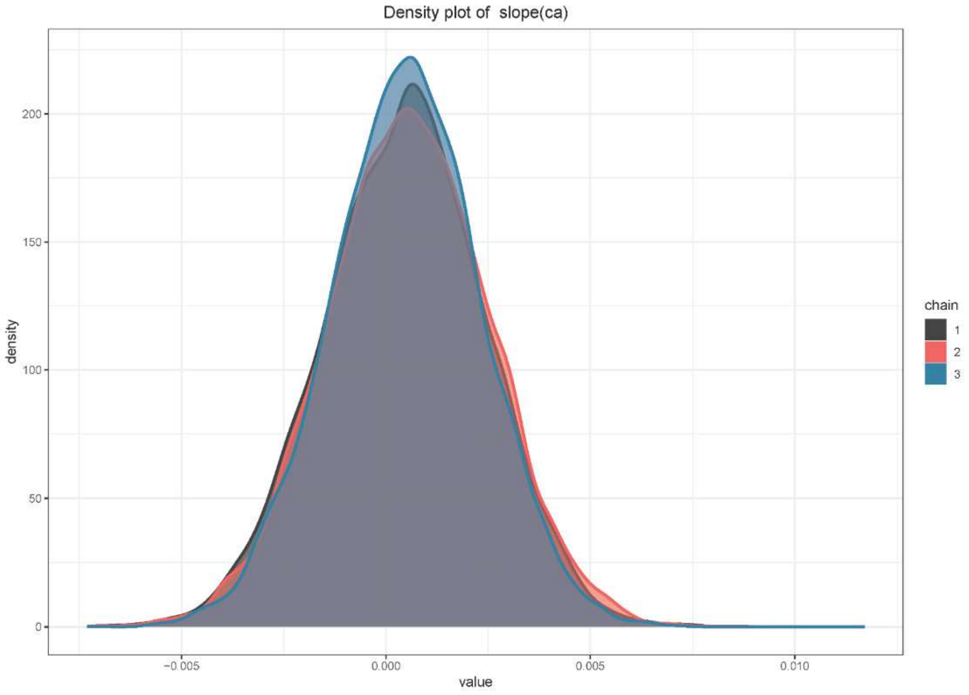

Association Parameters (α_1 and α_2) for Word Beginning

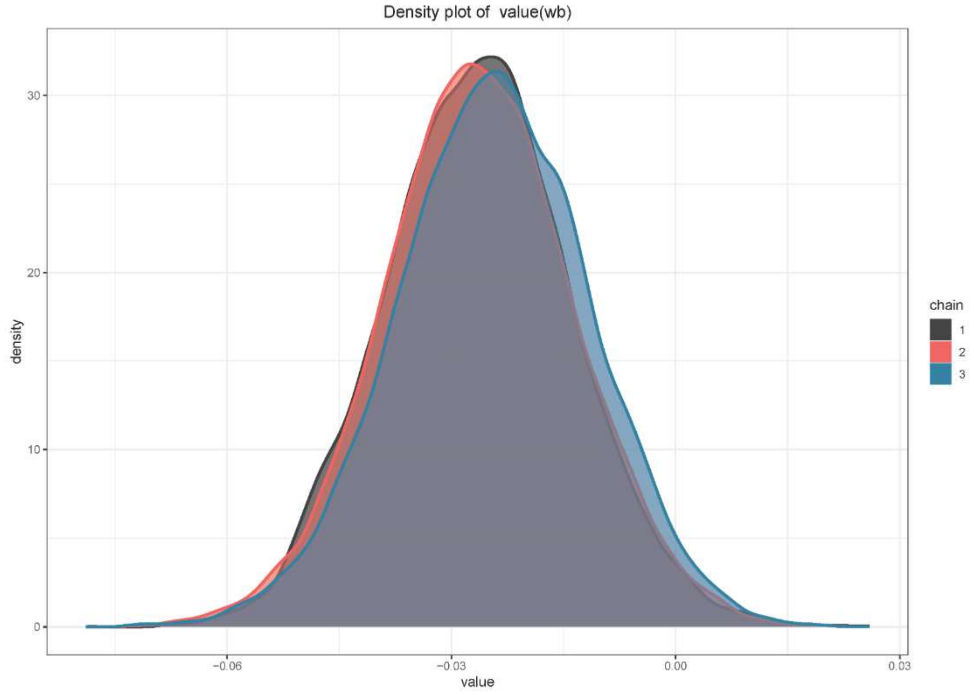

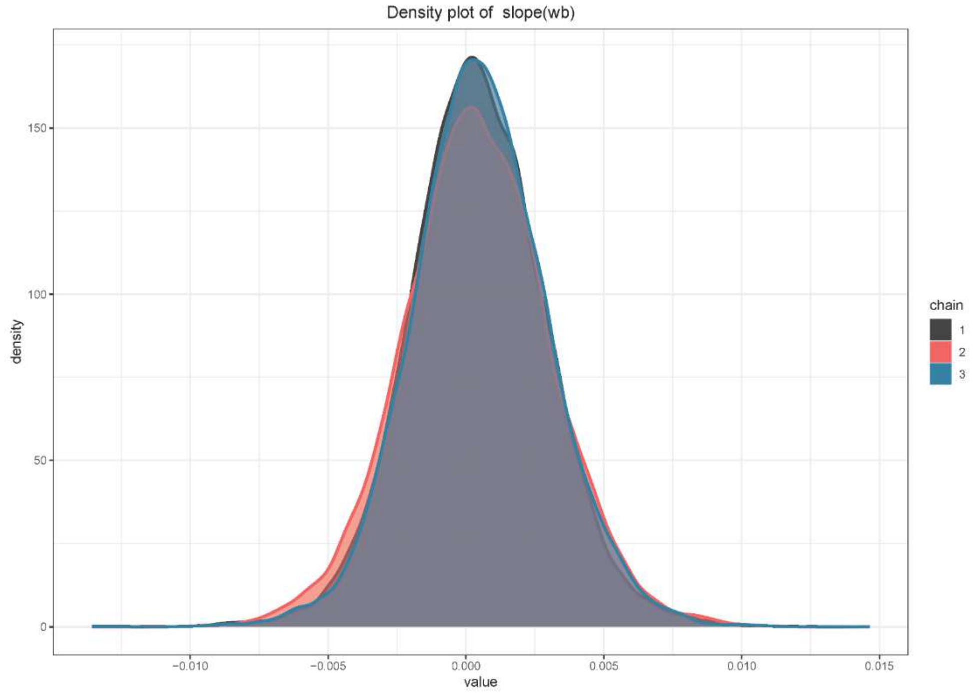

Association Parameters (α_1 and α_2) for Vocabulary

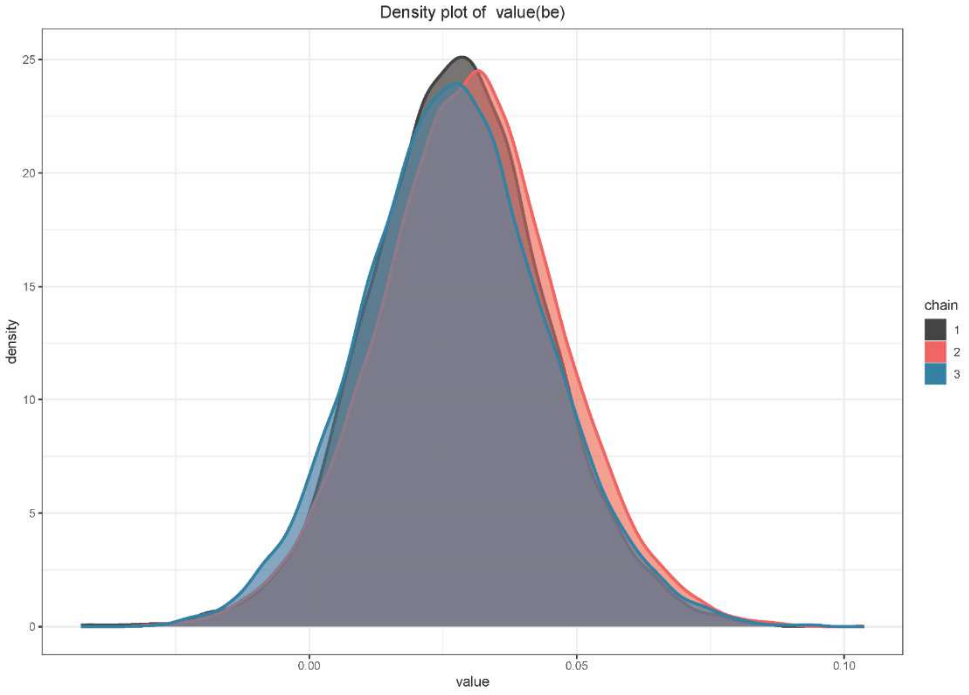

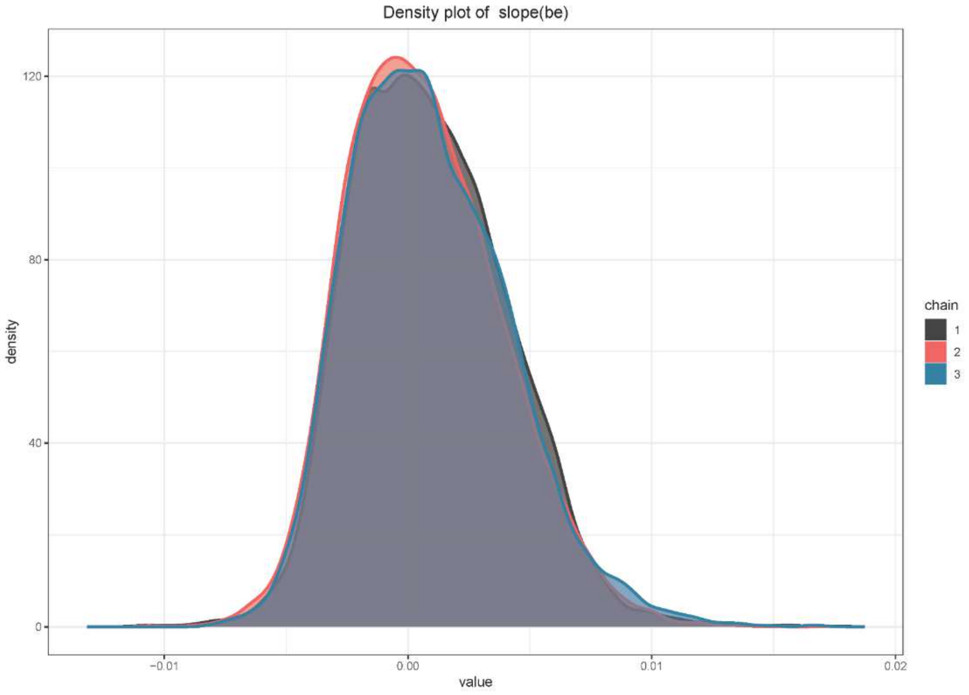

Association Parameters (α_1 and α_2) for Spot-a-Word

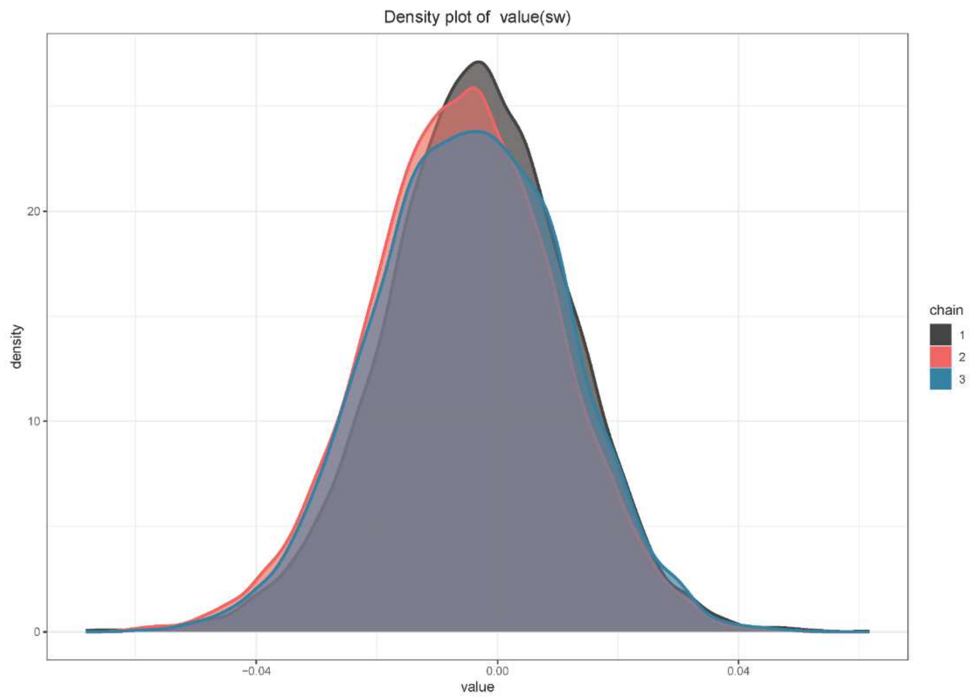

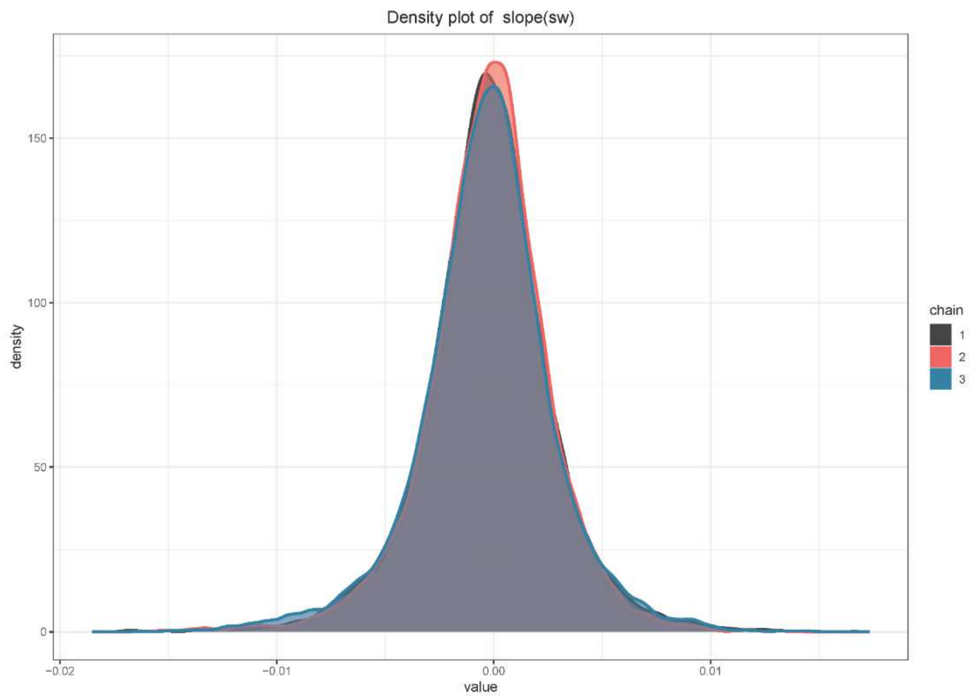

Supplementary Figure 3

Cumulative Quantile Plots of Survival Parameters (cf. `2_JMLSM.R` *on* https://osf.io/u57gr/files/osfstorage)

Initial Age, Sex, Socio-Biographical Status, and Suspected Dementia for First Chain

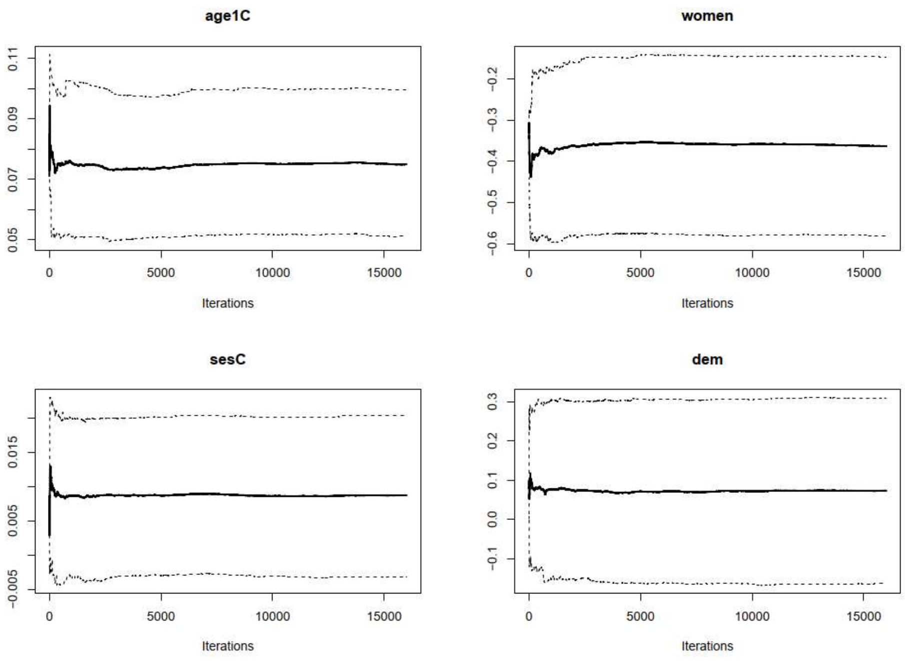

Initial Age, Sex, Socio-Biographical Status, and Suspected Dementia for Second Chain

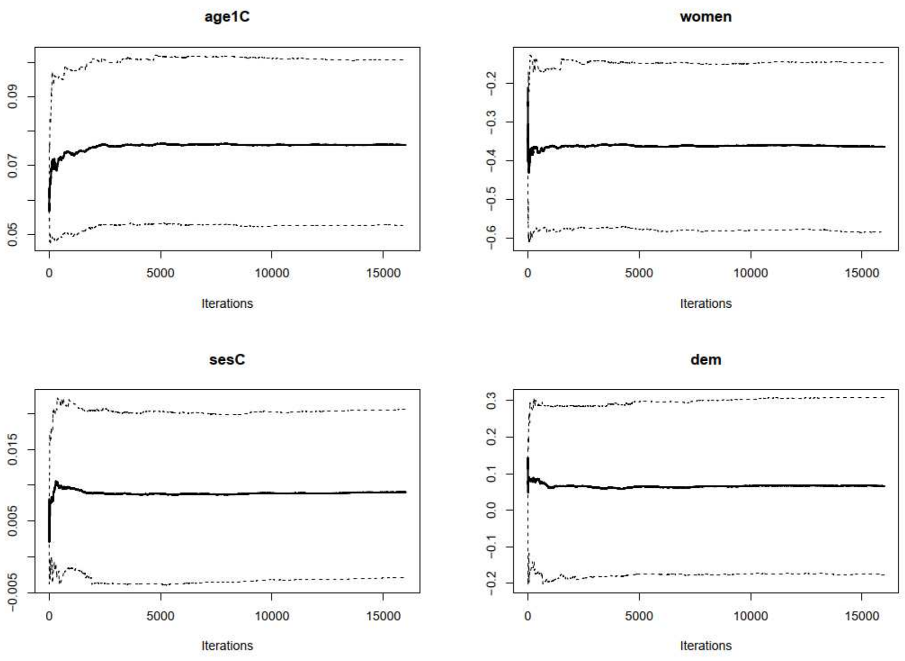

Initial Age, Sex, Socio-Biographical Status, and Suspected Dementia for Third Chain

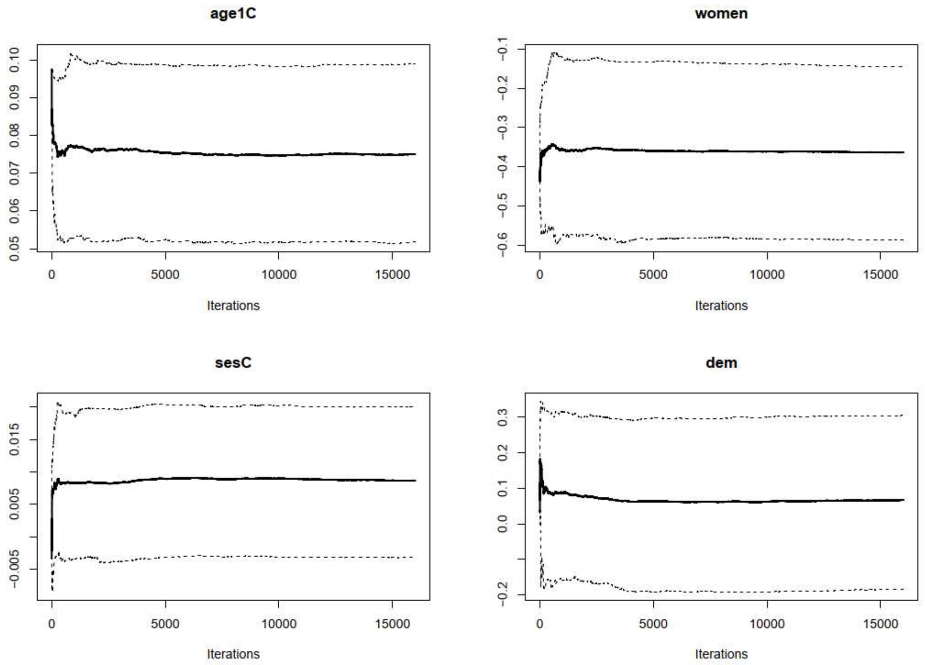

Association Parameters (α_1 and α_2) for First Chain

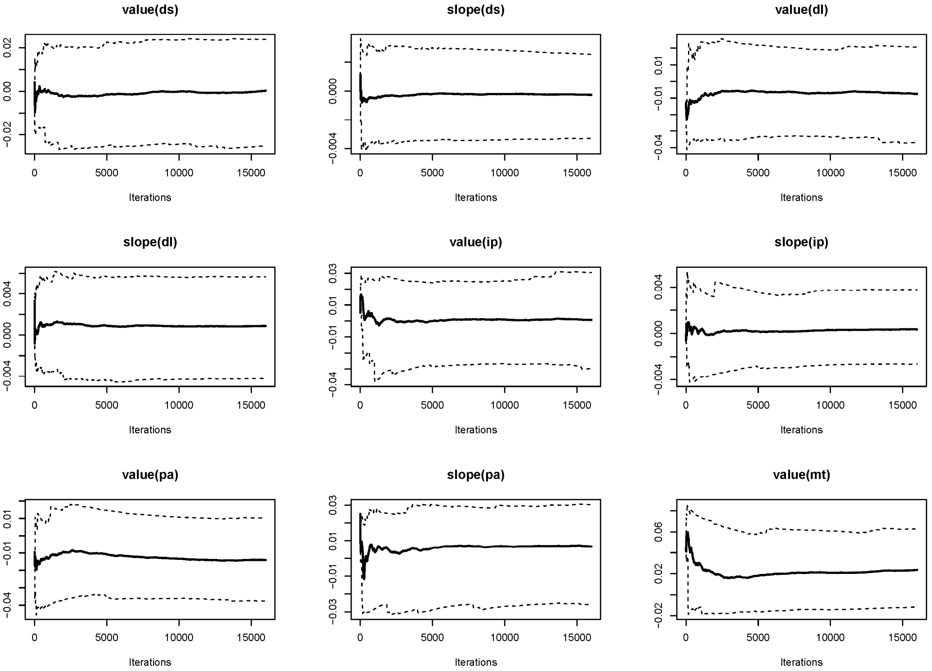

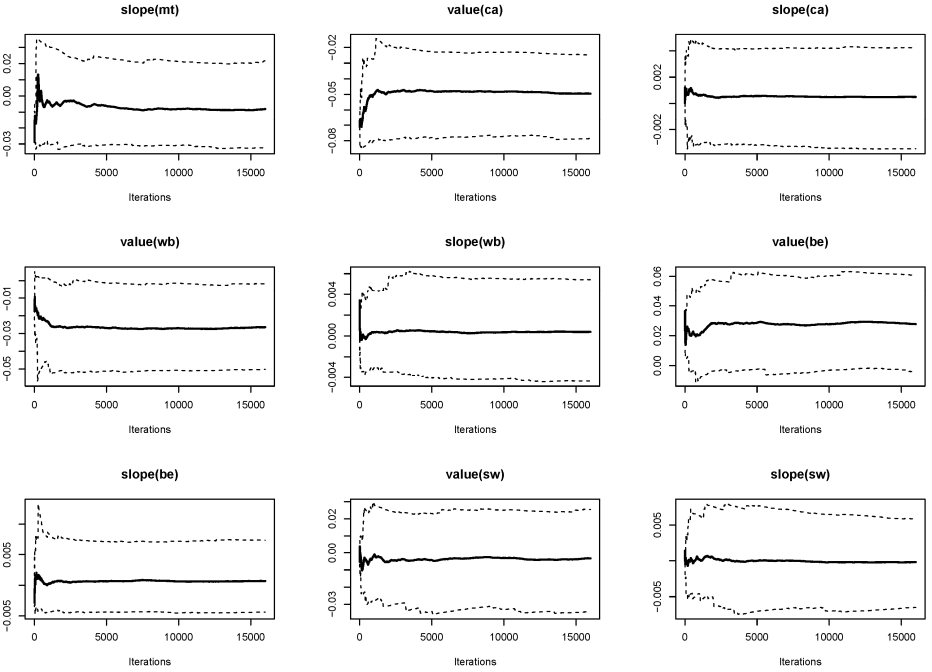

Association Parameters (α_1 and α_2) for Second Chain

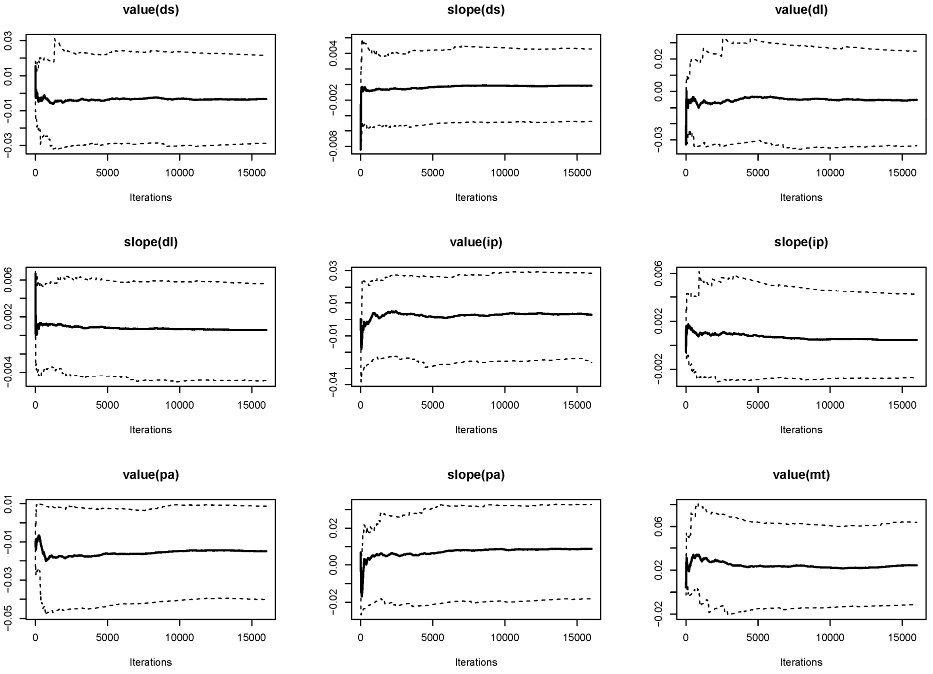

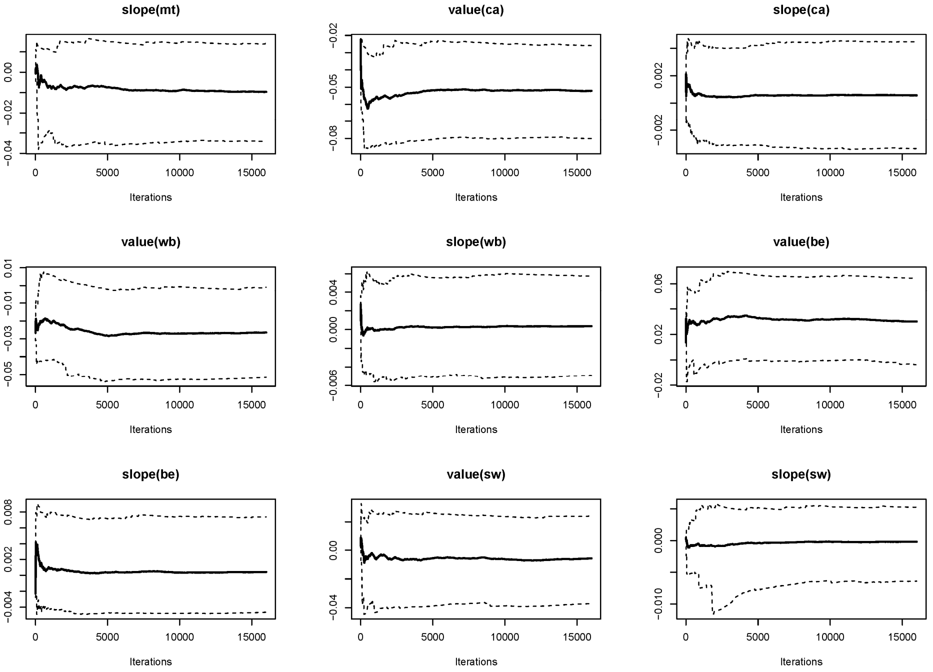

Association Parameters (α_1 *and* α_2*) for Third Chain*

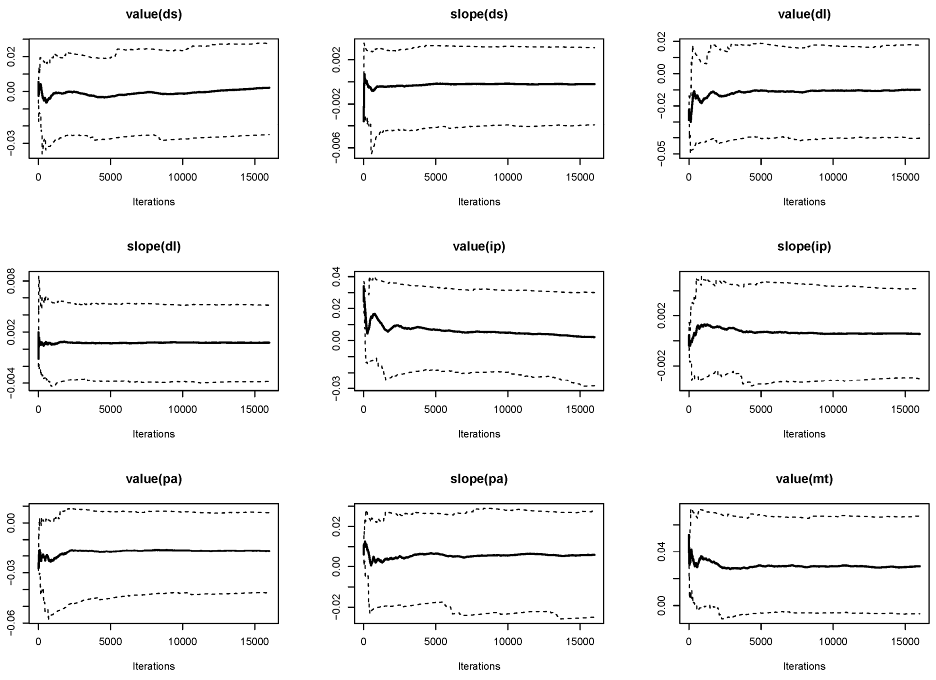

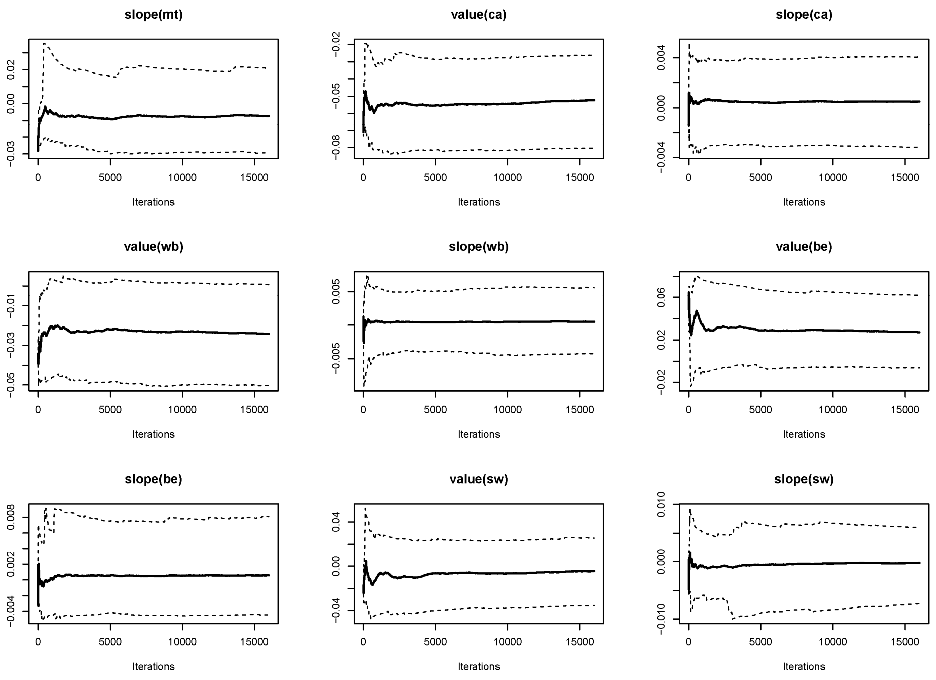

Supplement: sj-pdf-1-pss-10.1177_09567976241311923 – Supplemental material for Verbal Fluency Selectively Predicts Survival in Old and Very Old Age [file sj-pdf-1-pss-10.1177_09567976241311923.pdf]
